# Pockets to products: a data-driven approach for classification of monoterpene synthases

**DOI:** 10.1039/d6dd00312e

**Published:** 2026-09-18

**Authors:** Cathal Ó Raghallaigh, Nigel S. Scrutton, Sam Hay

**Affiliations:** a Manchester Institute of Biotechnology, Department of Chemistry, The University of Manchester 131 Princess Street Manchester M1 7DN UK Sam.Hay@manchester.ac.uk

## Abstract

Monoterpene synthases (mTSs) are a large family of enzymes, which have promising industrial applications, yet remain difficult to engineer due to complex and poorly understood sequence–function relationships. Here, we present a structure-based machine learning (ML) framework that accurately predicts whether a mTS is likely to produce linalool or limonene, chosen as canonical examples of linear and cyclic monoterpene products, respectively. Our approach identifies active site properties, which are used as ML features, enabling functional predictions that go beyond sequence alone. As residue positioning is important for monoterpene synthesis, we created an algorithm to identify structurally conserved residues in the active sites of mTSs, identifying new and existing motifs essential for both general catalysis and specific cyclisation steps. This integrated workflow thus provides insights into terpene synthases that were previously inaccessible and may offer a generalizable strategy for probing and engineering other poorly understood enzyme families. Future work could see this approach used to guide the rational design of mTSs and other hard-to-engineer enzyme families.

## Abstract 2

An interpretable structure-based ML framework uses active-site properties to predict linalool or limonene (linear/cyclic) production by monoterpene synthases identifying conserved motifs linked to cyclisation to inform future rational engineering.

## Graphical abstract

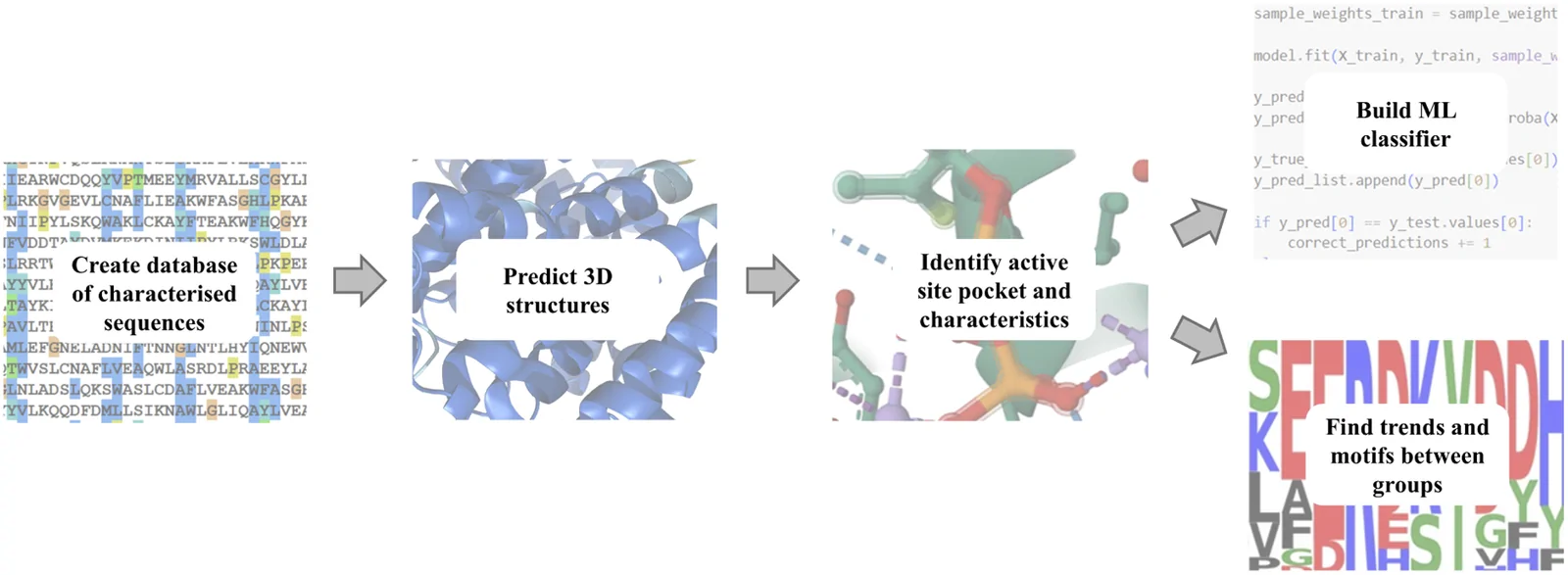

## Introduction

Deep learning models have led to an increased understanding of biological systems and have applications in drug discovery, fundamental structural biology and protein function/annotation.^1–3^ General ML approaches have been used in biology for decades. However, due to the limited availability of data, ML models were mainly represented in sequence-based approaches, with only a few examples using structural information, typically to supplement a sequence-based model.^4–8^ Thus, these approaches were less applicable with enzyme families which exhibit poor sequence–function relationships, and those that rely on structural information for engineering. One such example is cytochrome P450 enzymes,^9–11^ which require exploration of very large sequence spaces because of poor sequence–function mapping, and where ML methods have been developed to address this challenge.^12^ Another example is β-lactamases,^13,14^ where structural features account for the largest variation in functional data,^15^ and serine proteases,^16,17^ which share neither sequence homology nor overall fold, yet perform the same proteolytic function through conservation of catalytic residues within their active sites.^18^ Recent sequence-based enzyme-function models have continued to improve functional annotation from sequence alone, but such methods may not be suitable for enzymes with poor-sequence function relationships.^19–21^ However, the recent advancements in deep learning structure prediction offer the possibility of applying ML approaches to complex structural systems, due to the availability of fairly accurate structures and libraries like the AlphaFold database.^22^ Structural information has recently been used to build supervised learning models to understand enzyme specificity in other enzyme families with poor sequence–function relationships, including the use of AlphaFold-derived structural features to predict donor specificity in GT-B glycosyltransferases.^23^ Transfer learning and protein language models have also been applied to enzyme-function prediction, including substrate prediction in RiPP biosynthetic enzymes^24^ and the prediction of enzyme-catalysed reactions, enzyme functions and EC classifications.^25^

**Fig. 1:**
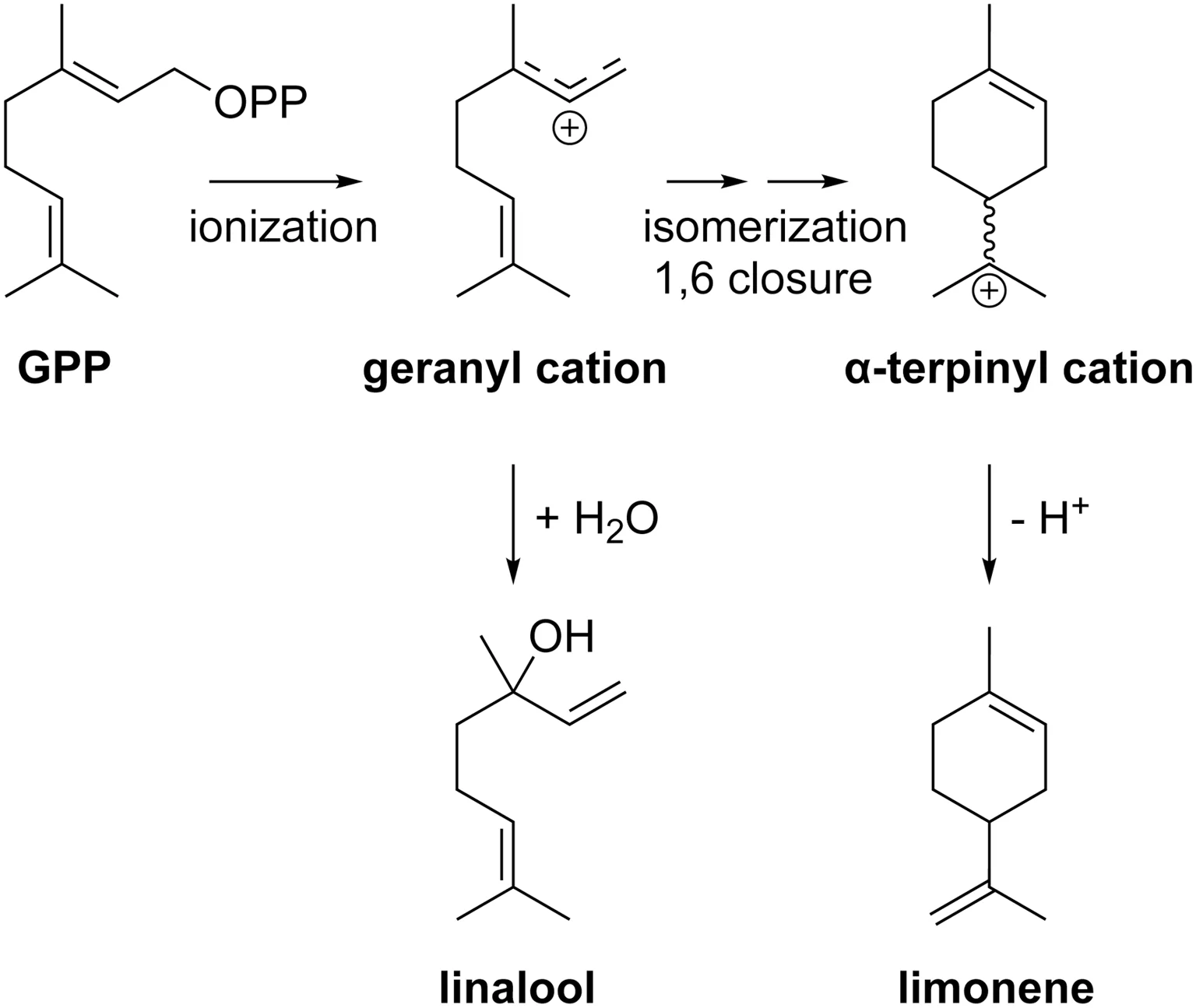
Simplified reaction scheme showing the mTS-catalysed formation of linalool and limonene from GPP. Adapted from ref. 35.

Terpene synthases (TS) are generally considered to be difficult to engineer, marked by weak sequence–function relationships and high sequence plasticity, where even minor amino acid changes can cause major shifts in product profile.^26,27^ Structural insights are further hampered by the scarcity of X-ray crystal structures, limiting understanding of product preferences and complicating rational engineering efforts.^26–28^ Although investigation into single, double and more additive mutations has been performed previously, rational design is limited as the complexity of interactions increases exponentially with each additional mutation.^29–31^ Despite this, the monoterpene synthase (mTS) family of enzymes remains an attractive engineering target as they typically catalyse the conversion of a single substrate (geranyl pyrophosphate; GPP) into a wide range of monoterpene products (Fig. 1). These products have widespread applications in fragrances, pharmaceuticals, and biofuels, including jet biofuel.^32^ Traditional monoterpene production methods face significant drawbacks, such as high environmental costs, water use, pesticide dependency, and the complexity of resulting product mixtures requiring extensive purification.^33,34^ Enzymatic synthesis potentially offers a cleaner, more selective alternative. This, together with the fact that terpene synthases catalyse some of nature's most complex chemistry,^35^ makes them an important system for both fundamental study and engineering.

While mTSs exhibit weak sequence–function relationships, mechanistic details are known. Key conserved motifs, such as the DDxxD metal-binding motif, are known to coordinate divalent metal ions and the phosphate moiety of the substrate which facilitates carbocation formation.^36^ Steric effects and pocket geometry can influence cyclisation.^35,36^ Specific active site histidine residues have been shown to promote α-terpinyl cation formation,^29,37^ and cysteine is linked to differences in cyclisation type rather than the initial 1,6-ring closure.^31,38^ Polar residues have been shown to affect the number of cyclisation events,^31,39^ and charged residues have been shown to affect product specificity.^40–42^ Hydrophobic residues have also been linked to controlling water during the formation of hydroxylated monoterpenes (such as linalool),^35,42,43^ and the role of active site aromatic residues has been well studied and mutational studies have shown that removing aromatic residues often results in the loss of cyclisation.^29,35,36,39,42^ For example, in a study on (4*S*)-limonene synthase, the W324A active site mutation led to a significant decrease in enzyme activity and altered product profile from (cyclic) limonene to linear products, but replacing the W324 residue with other aromatic residues did not rescue activity.^29^ Histidine residues have also been shown to affect linear product formation^29^ and phenylalanine has also been shown to play an important role in linalool production where the addition of phenylalanine to the active site of a bicyclic (1,8-cineole)-producing mTS causes loss of cyclisation and the production of linalool. However, when a separate phenylalanine was removed it also lost cyclic production capability.^42^ Overall, aromatic residues have been shown to prevent early quenching of the carbocation by stabilising the positive charge through cation–π interaction,^36^ which is thought to be important in formation of cyclic monoterpenes (Fig. 1).^42^ However, these mechanistic insights are typically derived from individual enzymes or small sets of variants and do not readily translate into more general predictive rules. In particular, small changes in active site composition or residue positioning can lead to large shifts in product profile, making it difficult to infer function directly from sequence.^26,31,39^ As a result, predicting mTS product specificity remains a non-trivial problem, even in the presence of established mechanistic knowledge.

ML methods present a promising solution to elucidate complex, non-linear relationships between enzyme structure, sequence, and product profiles, thereby enabling more precise and scalable engineering strategies. Given their complexity of understanding mTSs, ML is particularly attractive for terpene synthase research, and various ML/AI methods have been used in preliminary analysis,^44^ molecular replacement,^45^ and as the basis of screening platforms.^46–48^ In fact, previous work on sesquiterpene synthases showed that homology modelling data could enhance sequence-based approaches and reduce the species-related bias in an XGBoost based model.^8^ This model used whole-structural data. However, active site information plays a more important role in terpene synthase catalysis, given the lack of sequence–functional relationship and that mutational ‘hot-spots’ are around the active site.^26,27,31,36^ It follows that a model based on these features would be more accurate as it would have less ‘noise’. There has also been pre-print published which aims to use ML to understand TSs.^49^

A particular challenge in engineering mTS enzymes involves the generation and control of the α-terpinyl cation, which is necessary for the cyclisation step that unlocks increased chemical diversity of mTSs (Fig. 1).^50^ In this study, we investigate features of the mTS structure–function relationship that give rise to linear *vs.* cyclic product formation, selecting linalool and limonene as canonical examples of linear and cyclic monoterpenes, respectively due to their chemical similarity and the availability of characterized data. We developed an interpretable workflow that uses active-site structural information to distinguish linalool- and limonene-producing mTSs. Unlike uninterpretable protein language models,^24^ models targeting broader enzyme classes,^25^ whole-structure,^23^ broader terpene classifiers,^49^ and sequence-focused sesquiterpene models,^8^ our workflow predicts a specific difference in terpene end-product formation: cyclisation. To do this we use AlphaFold2,^51^ to predict structures of mTSs with experimentally characterized product profile data showing linalool or limonene as a major product,^52–105^ Fpocket^106^ to identify the active site, statistical methods to explore functional motifs, and we build a predictive ML model that can discriminate between mTSs that catalyse the formation of linalool or limonene as a proof of principle that links mTS sequence to a specific function (cyclisation).

## Methods

All code, AlphaFold structures, Fpocket output and ML training and evaluation data used in this paper is freely available at https://github.com/cathaloraghallaigh/ATC. The dataset was started using data provided by Dr Nicole Leferink (University of Manchester), which had 18 suitable sequences. It was expanded by searching through all ‘linalool’ and ‘limonene’ annotated sequences on UniProt,^107^ to find those with published GC-MS data confirming their classification. An exhaustive manual literature search was also carried out to find any mention of linalool or limonene synthases, and the papers were then checked to find if the enzymes had been experimentally characterized. A0A8H4VHP2 and O22340 were not included, as their inclusion reduced model performance. This dataset of sequences, their host organism and the relevant papers associated with them can be found in Table S1.

AlphaFold2.1.1 (ref. 51) was used for all structure predictions. The ‘best’ relaxed model (highest pLDDT) was chosen in each case. Active site information was generated using Fpocket^106^ with standard settings, which provides pocket-level descriptors and identifies the residues lining each predicted pocket. These outputs were used to derive active-site features describing pocket geometry, physicochemical properties and amino acid composition; the full feature list is provided in Table S2.^108^ This workflow (from original protein sequence to active site characteristics in a dataset) is mostly automated. The only manual section of this process is identifying which ‘pocket’ is the active site by visual inspection, as Fpocket typically identifies multiple pockets. This was performed based on the conserved architecture of monoterpene synthase active sites, including conserved active site motifs such as the DDxxD metal binding motif, and by comparison with the active site location in X-ray crystal structures through structural alignment. Fpocket^106^ was selected because it is an open-source and widely used cavity detection algorithm,^109–111^ which can be easily integrated into automated workflows, as its outputs are provided directly as PDB files. This allowed the entire pipeline from structure prediction to feature extraction to be as automated and reproducible as possible. In addition, Fpocket provides a relatively large number of quantitative pocket descriptors, and the algorithm also identifies the residues lining each pocket, enabling extraction of active-site amino acid composition for downstream analysis and model training. Structural sensitivity was assessed by repeating pocket identification and feature extraction using the second- and third-ranked AlphaFold2 models. Structures with pocket residue-count similarity below 75% of the original model were excluded to remove pockets affected by pinching or extension beyond the active site. Remaining structures were compared using feature similarity and leave-one-protein-out classification with the original model hyperparameters (Table S9).

Computational analyses were carried out in Python. Statistical analyses were performed using SciPy, pandas, and the built-in collections module. Data visualization and sequence logo generation were conducted with Matplotlib, seaborn, Logomaker, and Biopython. Machine-learning models were implemented with NumPy, scikit-learn, and XGBoost. Aligned protein structures (using the cealign PyMOL command) were analysed by extracting Cα coordinates from multi-model CIF files. Reference positions were defined as the Cα coordinates of active-site residues identified by Fpocket in the reference structure. For each reference position, the nearest Cα atom in every other protein was identified within a distance cutoff (4.0 Å). To maintain one-to-one correspondence, an assignment procedure resolved conflicts: if two positions competed for the same residue, the closer match was retained, and the displaced position was reassigned to its next best candidate, if there is one. This prevented residues from being reused across targets within a protein. Proteins were then grouped according to functional labels, and residue frequencies were calculated at each reference position for every group. Positions showing residues at ≥15% frequency were summarized with their group differences (Table S10), and sequence logos were generated to visualize residue distributions across groups. In addition, the script outputs per-protein tables listing the nearest residues to each reference position together with their distances, so individual proteins of interest can be investigated.

ML models were built to use active site features for binary classification (linear *vs.* cyclic products) using the XGBoost algorithm.^112^ XGBoost classifiers were trained with largely default hyperparameters: log-loss evaluation metric, histogram-based tree construction, L1 regularization *α* = 0.1, L2 regularization *λ* = 1.0 and learning rate of 0.1. Computations were accelerated on CUDA-enabled GPUs for the Bayesian optimization XGBoost models but CPUs were used instead for the rest, to ensure reproducibility. Feature importance was assessed using the gain metric in XGBoost, which measures how much each feature helps improve the model's predictions. Class imbalance was addressed by applying inverse class-frequency sample weights during training. Model selection targeted leave-one-out cross-validated (LOO-CV) accuracy while using scikit-optimize. Training used inverse-frequency sample weighting, where each observation was weighted by the reciprocal of its class prevalence to balance contributions from the linear and cyclic classes. Accuracy was optimized using leave-one-out cross-validation (LOO-CV) accuracy as the objective within a scikit-optimize Bayesian optimization (gp_minimize) framework over categorical feature-group choices. The acquisition function was set to Lower Confidence Bound (acq_func = “LCB”) with an exploration parameter (kappa = 10.0) to promote exploration of uncertain regions. Early stopping was applied when improvements in LOO-CV accuracy fell below 1 × 10^−4^ for 100 successive evaluations. Although the search allowed up to 500 evaluations, peak performance was reached within the first 100 iterations. Following feature selection, the FS1 features and hyperparameters were fixed. The model was trained on the 80% test/train set and evaluated on the independent holdout set, with performance assessed using accuracy, balanced accuracy, MCC, and per-class precision, recall, specificity and *F*1 score. Given the small dataset, model robustness was further assessed across the complete dataset using repeated stratified five-fold cross-validation with 10 repeats. To provide insight into feature importance and the direction of individual feature contributions across full dataset, fold-level XGBoost gain and out-of-fold SHAP^113^ values were also calculated.

Further model generalisation was tested using enzymes selected from a published sesquiterpene synthase dataset.^8,114^ Enzymes classified as linear or producing exclusively monocyclic products through a single 1,6-cyclisation were selected. AlphaFold structures were generated as described above and analysed using Fpocket. Only complete, structurally plausible models for which Fpocket identified a coherent pocket at the expected active-site location were retained, giving 66 enzymes comprising 39 linear and 27 cyclic synthases. The 12 features comprising FS1 and the XGBoost hyperparameters established using the mTS dataset were retained without further feature selection or optimisation. Balanced sample weighting was used to account for class imbalance. Performance was assessed using repeated stratified five-fold cross-validation with 10 repeats.

## Results and discussion

**Fig. 2:**
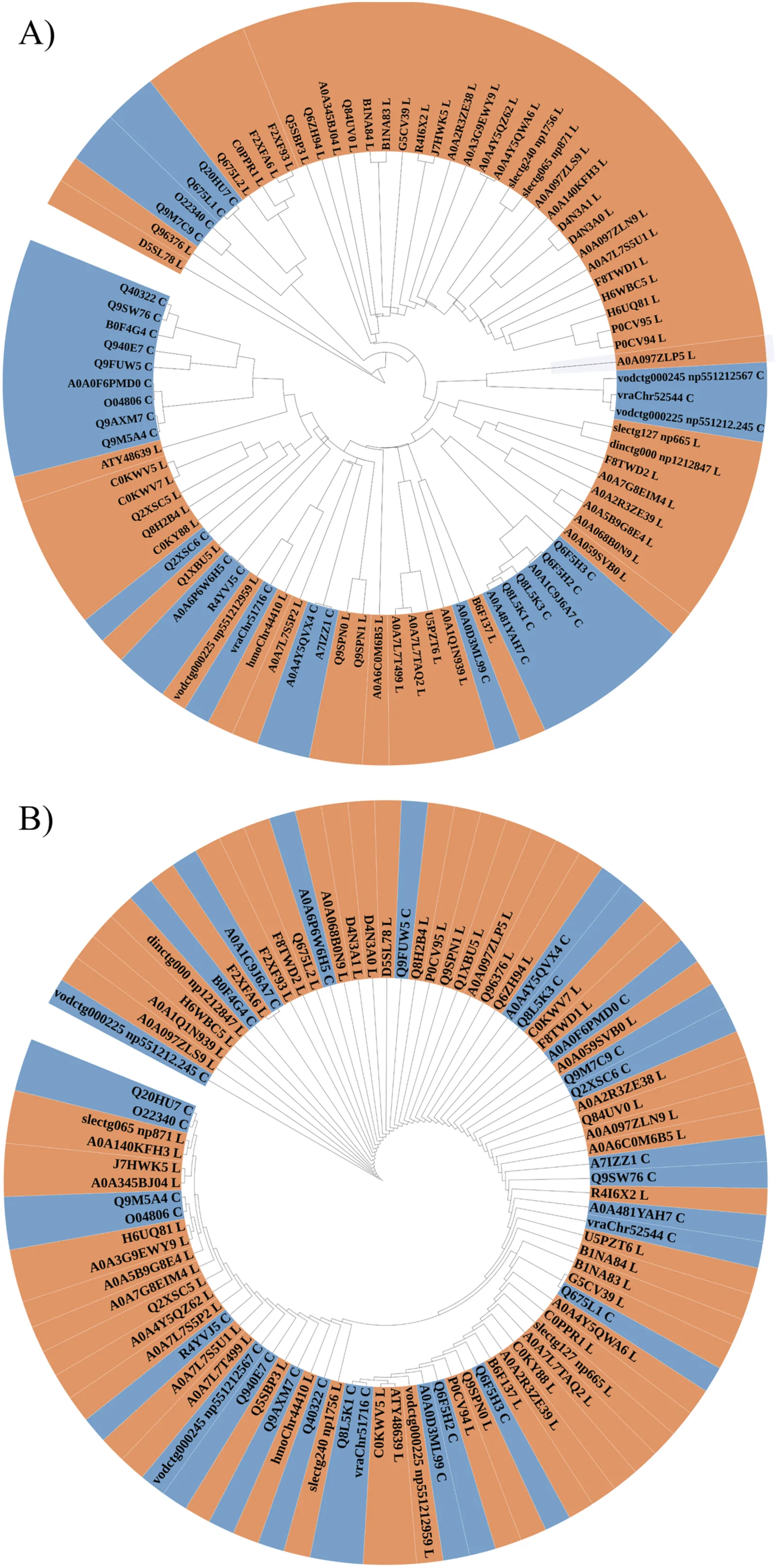
Phylogenetic analysis of the 88 monoterpene synthases (mTSs) in our curated library, colored by product preference: linalool (linear, orange) and limonene (cyclic, blue). (A) Sequence-based tree generated from a multiple sequence alignment using Clustal Omega,^118^ representing evolutionary relationships inferred from protein-sequence similarity. (B) Structure-based tree constructed from pairwise structural alignments obtained with ColabAlign,^119^ grouping enzymes by similarity in their overall three-dimensional structures. Both trees were visualized using the interactive tree of life (iTOL) software.^120^ Enlarged versions of both trees, labelled with UniProt accessions and scientific species names, are provided in Fig. S1.

We first compiled a library of 88 experimentally characterized mTSs, containing 29 cyclic and 59 linear producing enzymes (Table S1); each enzyme has a published product profile determined using GC-MS.^52–105^ A UniProt search of any proteins labelled linalool or limonene synthase was combined with a literature search of limonene and linalool producing enzymes, and all enzymes in this library have been reported to produce either linalool or limonene as a major product, which we define here as where ≥70% of the reported products belong to either the linear or cyclic class. This threshold was selected because the reported product distributions clustered above 70% for either the linear or cyclic class. We graphed the structural and phylogenetic relationship between the enzymes in tree format (Fig. 2). The sequence-based tree reflects evolutionary relationships inferred from protein sequence, whereas the structure-based tree groups enzymes by similarity in their overall protein structures. There seems to be some clustering between the groups. However, clear discrimination between the groups is not apparent, which is understandable, as previously stated, the sequences are ‘plastic’ and only small residue changes (mostly within the active site) cause a change in product profile. This is further exemplified when we carried out homology-aware evaluation of sequence-based nearest-neighbour classification, which achieved 66.7% accuracy with BLAST^115^ and 61.6% with phmmer,^116^ whereas structure-based classification using Foldseek^117^ achieved 78.2% accuracy (Table S18).

Due to the lack of availability of experimental structures for most enzymes in the library (available X-ray crystal structures found in Table S3), structures were predicted for all enzymes using AlphaFold2.^51^ As predicted structures are increasingly used as inputs to downstream ML pipelines, benchmarking structure-prediction performance on curated experimental datasets remains an important prerequisite for reliable biological inference.^121^ Where X-ray crystal structures were available, comparison with predicted structures generally showed good agreement, including when structures were predicted when the corresponding X-ray crystal structure(s) were excluded (“blacklisted”) from the template selection in AlphaFold, and also when the enzymes were predicted to be homodimers (we have modelled them all as monomers here; Tables S4–S8). Analysis of one X-ray crystal structure, PDB: 8GY0 (enzyme: A0A8H4VHP2) showed poor agreement between the predicted and experimental structures (Table S7) and inclusion of this enzyme in subsequent analysis resulted in a decrease in model accuracy. Consequently, we identified this as an outlier and have not included it in our dataset.

**Fig. 3:**
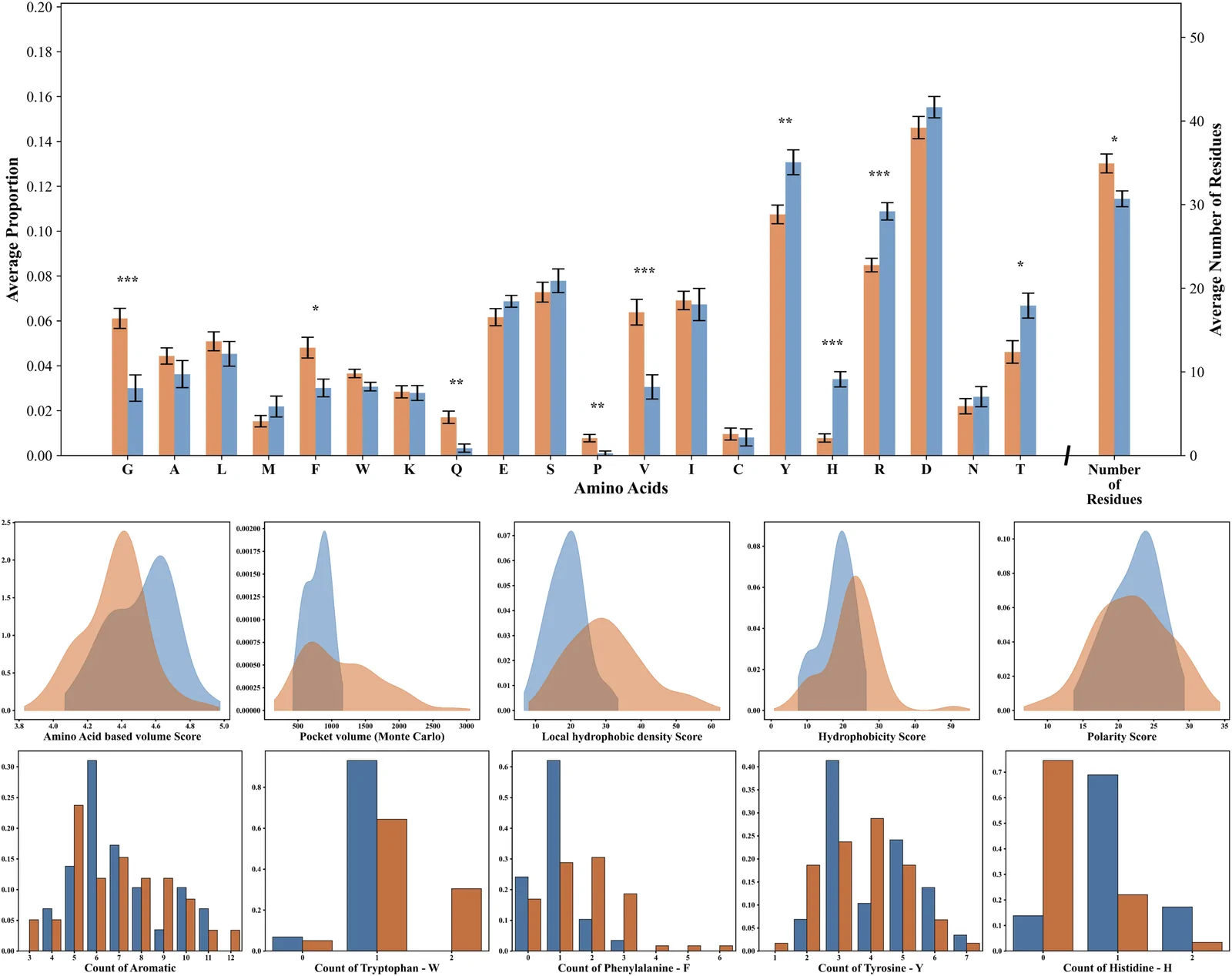
Statistical analysis of the active sites of the linear (orange) and cyclic (blue) mTS groups. Shown are the amino acid frequency and number of residues (top), density plots of amino acid and pocket based volume scores, hydrophobicity and polarity (middle, measured in density) and aromatic residue statistics (bottom, measured in proportion per protein). A larger version of this figure is found in the supporting information (Fig. S5).

Previous structural analysis has shown that mTS enzymes can undergo an open to closed conformational change upon substrate binding, which involves the inward movement of the H-α1 helix and repositioning of a conserved active-site tyrosine.^76^ Comparison of the position of this residue in the AlphaFold models used in this study shows that they almost all (85/88) adopt closed-like conformations similar to PDB: 2ONG (Fig. S3B). The tendency of AlphaFold2 to predict ‘closed’ protein structures has been reported previously.^122^ Using the predicted structures, we next found active site information using the Fpocket^106^ cavity detection algorithm, which is based on Voronoi tessellation to identify protein cavities. Along with active site volume, charge and scores of other properties, this algorithm identifies residues within the active site pocket (Fig. 3).

Fpocket identifies residues that line the active site/cavity. Structural comparison of the active site residues identified by Fpocket highlighted relatively larger differences between AlphaFold models and X-ray crystal structures (Tables S4–S8). The worst agreement was observed for A0A1C9J6A7, where >4 Å RMSD in these residues was observed between the X-ray crystal structure 5UV0 and the AlphaFold model (Table S5). This X-ray crystal structure is in an open conformation^76^ and further inspection of these data showed that much of the variance comes from specific residues that are found within disordered regions that are not well resolved in the other A0A1C9J6A7 X-ray crystal structures. When excluding these residues, the active site heavy-atom RMSD falls to ≈2.6 Å. Crucially, Fpocket identifies most residues within the mTS active site despite considerable uncertainty in their positions.

The volume of the active site has been shown to play an important part in product profile, mostly for substrate specificity.^27^ However, the volume of amino acids within the active site have also been shown to be correlated to end product due to the contour causing different steric effects on the carbocation.^123^ As expected, the amino acid-based volume score significantly differs between the active sites of the cyclic *vs.* linear producing enzymes in our library (Fig. 3). The average van der Waals volume contributions of the residues lining the pocket is higher in the cyclic (limonene) group: mean cyclic scores 4.50 Å^3^ (SEM of 0.04), mean linear scores 4.36 Å^3^ (SEM of 0.02), *p* = 0.004. The overall size of the active site pocket appears to be more rigidly conserved within the cyclic group of enzymes, with the active site of the linear group on average being larger and more variable: mean cyclic scores 802 Å^3^ (SEM of 35 Å^3^), mean linear scores 1134 Å^3^ (SEM of 71 Å^3^), *p* = 0.0001. More generally, most active site features were found to be more variable in the linalool producing enzymes, but this may be due to the larger size of this dataset. Alternatively, it may be that mutations in cyclic mTSs are more likely to cause loss-of-function, as the α-terpinyl cation (Fig. 1) must be stabilised for longer and it must perform more complex chemistry, thus there is a stronger evolutionary pressure for cyclic monoterpene synthesis.^35,36^

The role of aromatic residues in the active site of TS has been well studied.^35,36,39,42^ On average, tryptophan was found with equal frequency in the active sites of limonene and linalool producing enzymes (Fig. 3). Conversely, there are significant differences between the frequency of other aromatic residues identified in the active sites of these two enzyme groups. However, when normalized by the number of residues identified in each active site, there is only a marginally higher proportion of aromatic residues in the limonene producing enzymes: mean cyclic ratio 0.225 (SEM of 0.008), mean linear ratio 0.200 (SEM of 0.006), *p* = 0.095. Hydrophobic residues have also been linked to controlling water during the formation of hydroxylated monoterpenes (such as linalool),^35,42^ which may explain why there is not a clearer difference between the hydrophobicity scores for two enzyme groups (Fig. 3). More generally, the hydrophobicity of TS active sites has been stated to play an important role in cyclisation, where, along with a more aromatic pocket, a more hydrophobic pocket would prevent premature quenching of the cation intermediate(s).^35^ However, our results here show that the hydrophobicity score (mean of hydrophobicity of active site residues^124^) is higher for the linear group: mean cyclic score 18.2 (SEM of 0.845), mean linear score 22.5 (SEM of 0.930), *p* = 0.001.

**Fig. 4:**
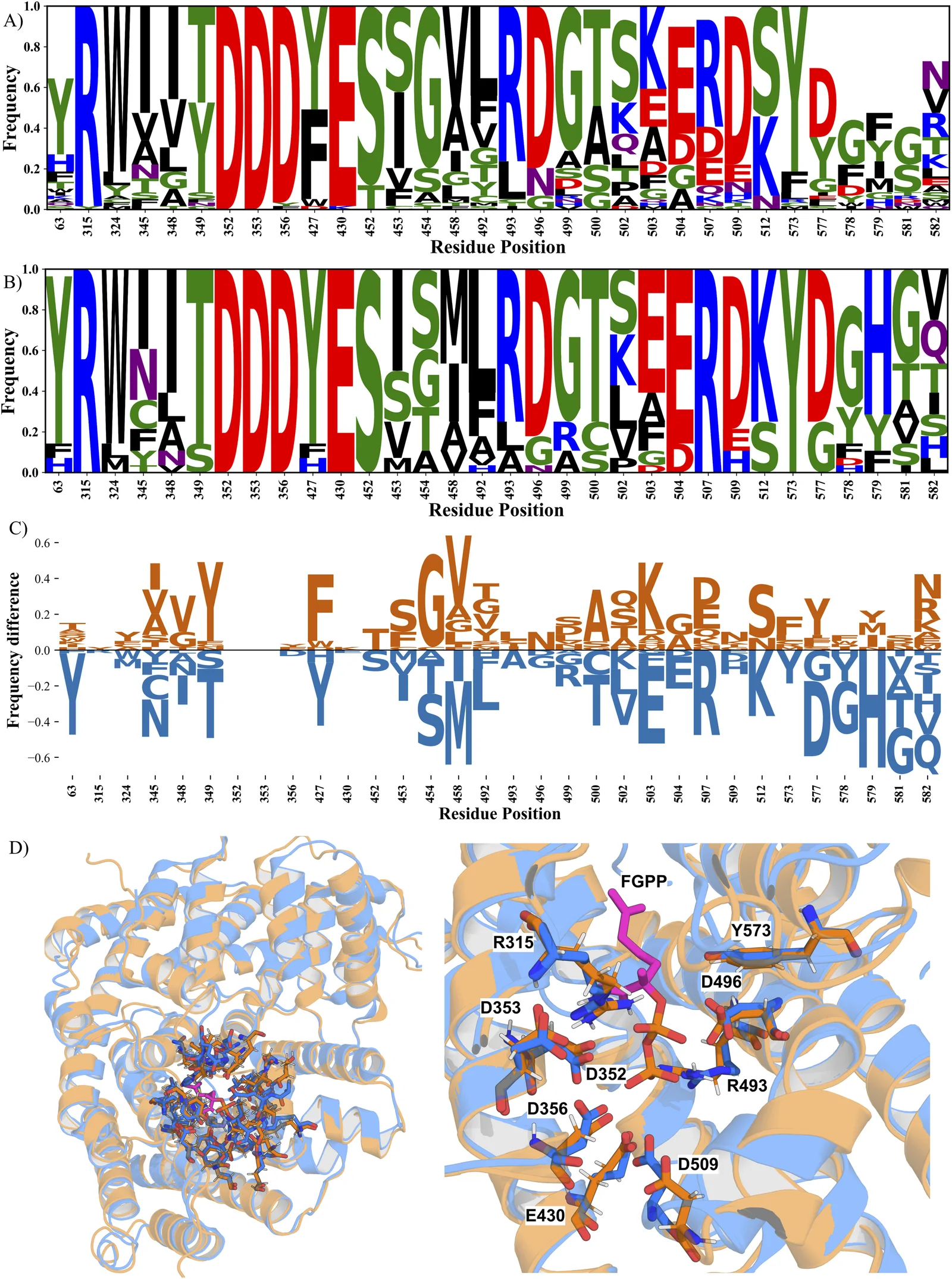
(A) and (B) Active site sequence conservation in the linear (A) and cyclic (B) members of the mTS library. Each active site sequence was determined using PDB ID: 2ONG as reference and an additional analysis using PDB ID: 5NX5 is shown in Fig. S6. Total letter height at each position indicates occupancy, where heights below 1 reflect enzymes for which no residue was identified within 4.0 Å of the reference position. Enlarged logos with separate occupancy plots are shown in Fig. S7. (C) Difference sequence logo showing residue-frequency differences between the linear and cyclic groups. Positive and negative values indicate greater residue frequency in the linear and cyclic groups, respectively. (D) Alignment of the AlphaFold2 model of F8TWD2 (member of the linear group; orange) with an X-ray crystal structure of a limonene synthase (PDB ID: 2ONG; blue). The all-atom RMSD is 1.08 Å. Conserved residues within the active site are highlighted right. The 2-fluorogeranyl diphosphate (FGPP) substrate analog and three Mn^2+^ found in the X-ray crystal structure are shown in magenta and purple, respectively. Fig. S2 shows an overlay of all active site residues in each enzyme.

Whilst the amino acid composition of the active site is important, the spatial arrangement of residues within the active site is expected to play an important role in enzyme catalysis.^125^ To investigate structural conservation of the active site, we aligned the structure of each enzyme in the library with the X-ray crystal structure of *Mentha spicata* limonene synthase (PDB ID: 2ONG), which we use as a reference structure. Alternative analysis using a linalool synthase gave similar results (Fig. S6 and Table S11). For each specified residue within the reference structure, the algorithm finds the closest corresponding residue for each of the proteins, with a 4.0 Å cut-off (Table S10). This effectively aligns the sequences of each active site, which is shown in sequence logo format in Fig. 4. A more detailed structural analysis was not attempted due to limitations of the structural prediction, which we do not expect to predict metal binding or fully reproduce loop rearrangement, correct side chain packing and H-bonding within the active site (Fig. S2 and S3).

The active site alignments identified established highly conserved residues and motifs across both groups of enzymes, including W324,^29^ and the metal binding D352/353/356 (part of the DDxxD domain),^36^ and Y573, which, despite being conserved highly in both groups, it is always conserved in the cyclic group. This conservation has previously been shown to be essential for cyclic monoterpene synthases.^39^ There are also conserved motifs which have not yet been shown, such as, R315, E430 and S452 which are likely to play important roles in monoterpene catalysis as they are conserved across both groups. In fact, serine can be involved in metal binding, but also has been shown to indirectly impact catalysis of terpene synthases in TSs.^31,126^ Mutating serine residues in the active site of mTSs has been shown to lead to slight change in product profile,^29^ or inactivation.^127^

There are some residues which are more commonly conserved in the linear group of enzymes, most noticeably, G454 (2ONG numbering). There are many residues which are more frequently conserved in the cyclic group of enzymes which suggests they play a role in the formation or stabilisation of the α-terpinyl cation. These include Y63, T349, Y427 (which are typically aromatic residues), E504, R507, K512, D577, and H579 (Fig. 4). Consistent with this, previous work has shown that removal of H579 results in reduced cyclisation capacity, as well as T349 and R507, to a lesser extent.^29^ Additionally, for the linear group, there also appears to be up to 5 residues missing from the ‘end’ of the active site sequence (frequency < 100% at a given position, with no corresponding residues within a 4.0 Å radius), which is due to some enzymes in this group having fewer residues associated with the active site. These ‘missing’ residues are at the surface of the protein, and in 2ONG are found in the J loop, which forms part of the ‘cap’ that could protect the cations from premature quenching,^128^ (Fig. S4). While our analysis in Fig. 3 and 4 provides insight into difference in mTS enzymes that produce cyclic *vs.* linear monoterpenes, we do not identify any discriminatory features (*i.e.* differences found only in one group). We reasoned that a ML model based on active site characteristics may be able to perform this classification (workflow diagram seen in Fig. S9).

Extreme gradient boosting (XGBoost)^112^ is a decision tree-based supervised ML algorithm that has been shown to build accurate classification models and can perform well with relatively small datasets.^129–131^ Additionally, as these models are more interpretable than black-box models, we can understand and learn from which features influence the ability of the model to distinguish between the groups.^132^ Due to the modest size of the dataset, leave-one-out (LOO) cross-validation was also used.^133,134^ This method of cross-validation leaves one dataset entry (*i.e.* enzyme) out of the training dataset for each round, only testing the one missing observation. This is then repeated for each entry within the dataset. This approach is more computationally expensive than other cross-validation methods, but it has been shown to minimize the chance of overfitting^135^ and has been used successfully in ML models for protein/enzymology before.^136–138^ Bayesian optimization (BO) has also been shown to be useful for feature selection for high-dimensional data used in training molecular biology XGBoost models.^139,140^

Whilst the spatial arrangement of active site residues plays an important role in enzyme catalysis, explicitly capturing this typically requires more complex models, such as geometric or graph-based approaches.^141^ Given the modest size of the dataset used in this study and the use of structure prediction (*cf.* experimental structures), such approaches are expected to lead to overfitting and may not provide additional value. We therefore opted to use Fpocket-derived active site properties, which captures key characteristics of the active site without requiring high-resolution structural detail or large ML models. Fpocket can generate up to 46 features describing the active site, including features describing the occurrence of each of the 20 individual amino acids. Using all Fpocket features (Table S2) would result in ML models with large search space, so to reduce the size of the feature space, non-amino acid features were grouped based on redundancy between the features. Prior to any feature-space exploration, the dataset was randomly split in a stratified manner, to a ∼80% test/train (69 members; 47 linear and 22 cyclic). All Bayesian optimization and feature selection steps were performed exclusively on the test/train dataset with the ∼20% validation set (18 members, 12 linear and 6 cyclic) reserved for final model evaluation.

**Fig. 5:**
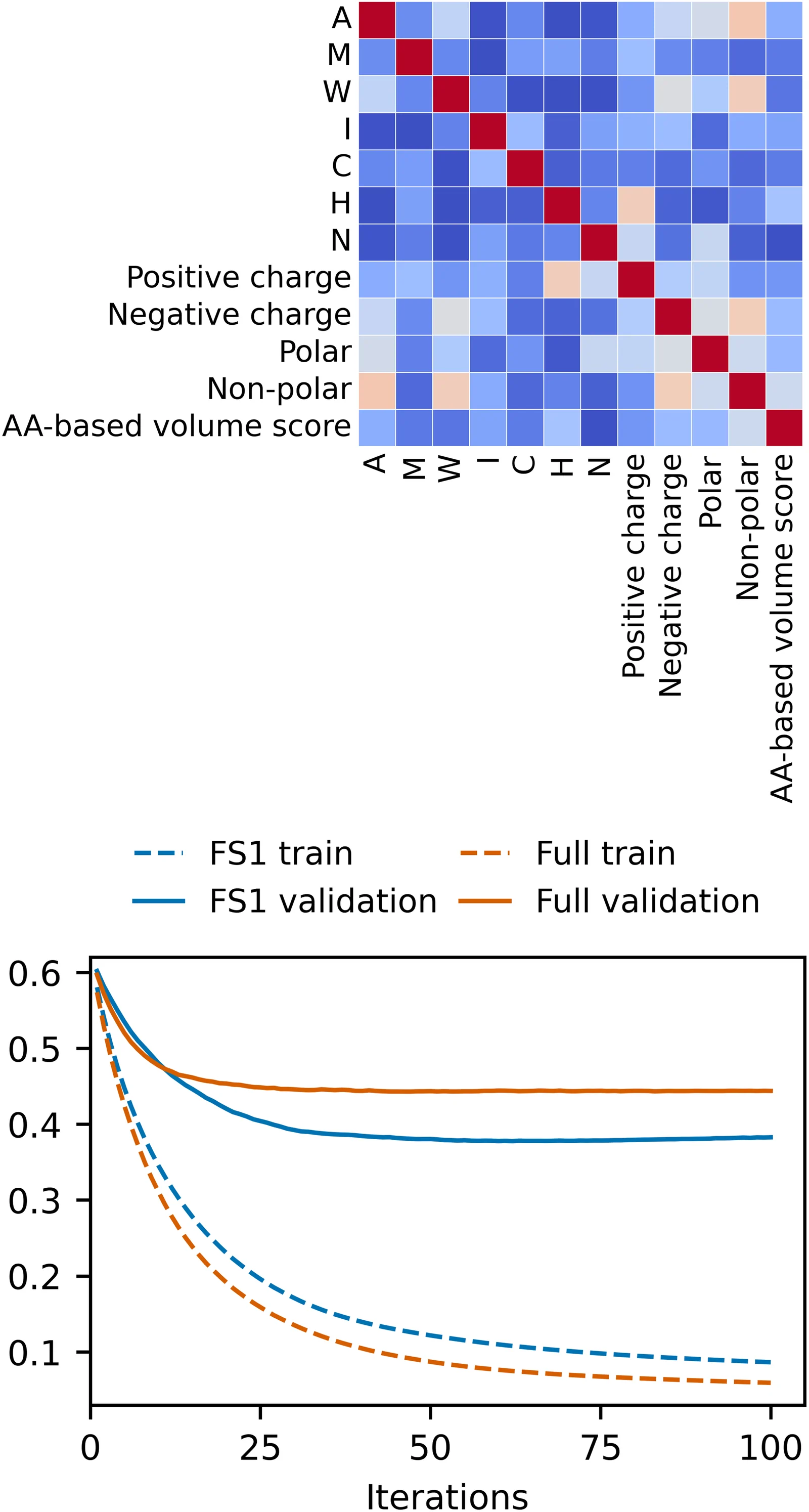
Evaluation of the 12-feature FS1 XGBoost model. (Top) Correlation matrix of the final FS1 features selected using leave-one-out cross-validation, showing low correlation between features. (Bottom) Mean training and validation log-loss curves obtained using repeated stratified five-fold cross-validation of the test/train set (69 observations; 10 repeats). The full feature set was included for comparison (final mean validation log loss: 0.442), with FS1 achieving a lower final mean validation log loss of 0.384. The final 12-feature FS1 XGBoost model was trained on the entire test/train set and evaluated on the independent holdout set (18 observations), achieving an overall accuracy of 94.4%, balanced accuracy of 95.8% and MCC of 0.886. For the linear class, precision was 100%, recall was 91.7%, specificity was 100% and *F*1 score was 95.7%. For the cyclic class, precision was 85.7%, recall was 100%, specificity was 91.7% and *F*1 score was 92.3%.

To elucidate the best combination of features for ML model training, BO was performed (Table S12). Features were grouped, allowing the reduction of the number of features whilst reducing the testing space needed. Combining BO with the LOO-XGBoost ML model resulted in a model with accuracy of 92.0% (Fig. S10–S12 and Tables S13, S14). The features identified in this model are denoted FS1. Using these features, log loss was calculated using stratified k-fold (5 folds, 10 repeats, Fig. 5). Here, the Bayesian-optimized FS1 model performed with higher mean accuracy (83.5 ± 8.4%) and lower mean validation log-loss (0.379) than the model trained with the full feature set (80.7 ± 8.2% accuracy, log-loss = 0.441). This supports the effectiveness of the feature selection and demonstrates that a reduced, interpretable subset improves overall model performance and generalization. The resulting 12-feature FS1 model, trained using the selected XGBoost parameters, was therefore taken forward as the final model configuration. The resulting 12 feature (FS1) model was then trained on the entire test/train set (69 observations; 80% of the dataset) and evaluated on the independent hold-out ‘validation set’ (18 observations; 20% of the dataset). This yielded an overall accuracy of 94.4%, with only one linear observation misclassified (UniProt ID: A0A6C0M6B5, confusion matrix in Fig. S13). Repeated stratified five-fold cross-validation across the complete modelling dataset (87 observations; 10 repeats) gave a mean accuracy of 90.0% (SD 2.4%), balanced accuracy of 89.2% (SD 2.9%), and MCC of 0.775 (SD 0.054; Fig. S13). As the dataset comprises a single enzyme family, similar sequences and structures occur across the training and validation sets. To assess whether this inflated model performance, leave-one-cluster-out evaluation was performed, giving accuracies of 87.4% for sequence clusters and 93.1% for structural clusters, with balanced accuracies of 86.9% and 93.0%, respectively (Table S18).

Further, we explore alternative classification methods using both the full feature set and FS1. This analysis included the use of additional methods, including Random Forest and logistic regression, ESM-2, phmmer and Foldseek, which all performed similarly, or worse than XGBoost (Tables S15, S18 and Fig. S15). This demonstrates the predictive power of active site information and indicates that this type of information is important for discriminating between the two groups. FS1 was selected to continue from here, given better overall performance.

**Fig. 6:**
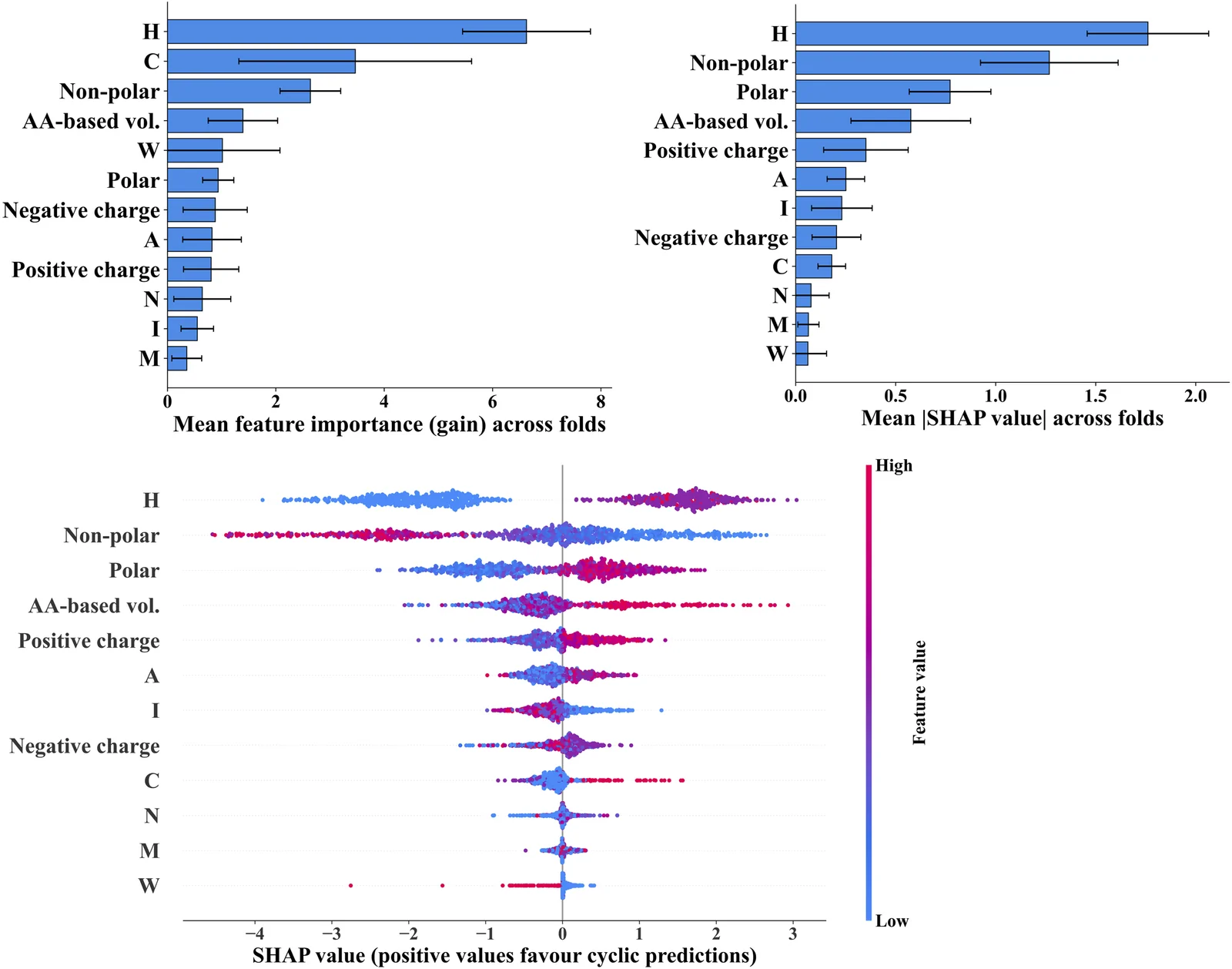
Feature importance for the FS1 XGBoost model across repeated stratified five-fold cross-validation of the complete modelling dataset. Top left: Mean XGBoost gain across the 50 fitted models. Histidine had a mean gain of 6.626 (SD of 1.181), cysteine had a mean gain of 3.466 (SD of 2.147) and non-polar residue count had a mean gain of 2.637 (SD of 0.560). Top right: Mean absolute out-of-fold SHAP importance across the 50 fitted models. Histidine had a mean absolute SHAP value of 1.761 (SD of 0.304), non-polar residue count had a mean absolute SHAP value of 1.268 (SD of 0.344) and polar residue count had a mean absolute SHAP value of 0.772 (SD of 0.204). Bottom: Directional SHAP values pooled across the out-of-fold predictions. Positive SHAP values favoured the cyclic class and negative SHAP values favoured the linear class. Higher histidine and polar residue counts favoured cyclic predictions, while higher non-polar residue counts favoured linear predictions. Error bars in (top left) and (top right) show the SD across the 50 fitted models.

Of the 12 features within FS1 (Fig. 6), histidine ranked first by both mean gain and mean absolute SHAP. Cysteine ranked second and non-polar residue count ranked third by mean gain, whereas non-polar and polar residue counts ranked second and third by mean absolute SHAP, respectively. Higher histidine counts favoured cyclic predictions, consistent with the reported role of histidine in α-terpinyl cation formation.^29,37^ Polarity-related variables (‘polar’ and ‘non-polar’) were also among the most influential features, with polar residue count ranking third by mean absolute SHAP. Higher non-polar residue counts favoured linear predictions, whereas higher polar residue counts generally favoured cyclic predictions, although the directional effect was weaker for polar residue count. Polarity-related active-site variables have been shown to affect cyclisation, however, primarily influencing the type or number of cyclisation events.^31,39^ Cysteine ranked third by mean gain but ninth by mean absolute SHAP and has been linked to differences in cyclisation type rather than the initial 1,6-ring closure.^31,38^ The importance and prevalence of these features in the final models may also be due to the fact the hydroxylation of the end products used for the model. It could be interesting to investigate how they contribute to mTS catalysis in future experimental work.

During preparation of this manuscript, a preprint from the Pluskal group reported a UniProt-mined terpene synthase dataset.^49,142^ There are 14 enzymes within the preprint dataset (5 of which produce limonene and 9 of which produce linalool) which are not present in our dataset (Table S17). To benchmark our approach, we refit the final XGBoost model (FS1, same hyperparameters/class weights) on the full training and validation data (87 observations) and evaluated it on these additional 14 enzymes, resulting in an accuracy of 92.9% (with only the limonene-producing, UniProt ID: G1JUH4, predicted incorrectly, Fig. S14). We also tested a selection of mTSs that catalyse the production of different linear and (bi)cyclic monoterpene products. Of these, 7/10 of the linear monoterpene-producing enzymes were predicted to produce linalool (*i.e.* linear), while 8/8 monocyclic and 4/9 bicyclic monoterpene-producing enzymes were predicted to produce limonene (*i.e.* (mono)cyclic), respectively (Fig. S16 and Table S19). This suggests that the ML model has some transferability to predict linear and monocyclic monoterpene products, but may not generalize to wider terpenes, as exemplified by the poor performance with the bicyclic monoterpene-producing enzymes. It would be interesting to use the new TPS dataset to extend the ML model to explore additional mechanistic steps in mTSs. However, this would require a different model architecture to do multi-class and/or multi-label (for multiple product) classification, which is outside the scope of the current study.

As a preliminary investigation of wider generalisability, the workflow was retrained using 66 sesquiterpene synthases, 39 linear and 27 monocyclic (1,6-cyclising) (Table S20) selected from a previously published dataset.^8,114^ This dataset covers enzymes producing a broader range of products than the predominantly linalool- and limonene-based mTS dataset. Without further feature selection, hyperparameter optimisation or model improvement, repeated stratified five-fold cross-validation achieved a mean accuracy of 84.4% (SD 3.8%), balanced accuracy of 84.1% (SD 4.0%), and MCC of 0.680 (SD 0.079; Fig. S17).

Although we cannot claim wider generalizability of the existing ML model, the approach taken here addresses a broader class of enzyme engineering challenges that are not unique to monoterpene synthases. In particular, mTSs share key characteristics with enzyme families such as cytochrome P450s,^9–12^ β-lactamases,^13–15^ and serine proteases,^16,17^ where weak sequence–function relationships and strong dependence on local active site chemistry complicate prediction and engineering. In this study, these challenges are addressed by explicitly defining and aligning structurally conserved active site residues and by representing functional differences through the local compositional and physicochemical properties of these regions, rather than relying on whole-protein or sequence-based representations.

## Conclusions

Here, we have developed a simple workflow that allows structure-guided sequence and feature comparisons of related classes of enzymes; in this case the limonene and linalool producing mTSs. This exploits structure prediction using AlphaFold2,^51^ active site identification and characterization using Fpocket,^106^ nearest-neighbor active site sequence alignment and an XGBoost^112^ based ML model with good classification performance. Our active site alignment algorithm identified a number of well-known mTS motifs, as well as identifying additional residues that appear to be important for general catalysis due to their conservation. Analysis of the relative importance of features in the ML classifier further identified some known features that aided in distinguishing between limonene and linalool producing mTSs (small, charged, uncharged, aromatic active site residues), with more that do not yet have a known direct effect on monoterpene cyclisation. This integrative approach offers a data-driven path to decoding enzyme specificity and as this method does not require mechanistic information, only requiring identification of active site residues, we anticipate that this can be readily integrated into (semi)-rational engineering approaches for terpene synthases and other ‘difficult-to-engineer’ enzyme families.

## Conflicts of interest

There are no conflicts to declare.

## Abbreviations

ML: Machine learning
mTS: Monoterpene synthase
TS: Terpene synthase
AS: Active site
mTS: Monoterpene synthase
BO: Bayesian optimization
LOO: Leave one out (cross-validation)
XGBoost: Extreme gradient boosting

## Supplementary Material

DD-005-D6DD00312E-s001

DD-005-D6DD00312E-s002

## Data Availability

All Python code, AlphaFold structures, Fpocket output and ML training and evaluation data used in this paper is freely available at https://github.com/cathaloraghallaigh/ATC and at ZENDO (https://doi.org/10.5281/zenodo.22877417). All additional data supporting this study are provided in the supplementary information (SI) accompanying this paper. Supplementary information: enzyme datasets and feature definitions, benchmarking of AlphaFold predictions against crystal structures, active-site residue analyses, and sensitivity analyses of structural model choice and residue mapping. It also provides machine-learning algorithm comparisons, cross-validation results, independent validation, and further testing on monoterpene synthases with other products and on sesquiterpene synthases. See DOI: https://doi.org/10.1039/d6dd00312e.
