# Supplementary material for: Pockets to products: a data-driven approach for classification of monoterpene synthases
Source: Digit Discov. 2026 Sep 18;5(10):4573–89. doi: 10.1039/d6dd00312e (PMC13613485; doi:10.1039/d6dd00312e)
Supplement: Supplementary file 1 [file DD-005-D6DD00312E-s001.pdf]

Supplementary Information (SI) for Digital Discovery.
This journal is © The Royal Society of Chemistry 2026

Supporting Information:

Pockets to products: a data-driven approach for classification of monoterpene synthases

Cathal Ó Raghallaigh, Nigel S. Scrutton, Sam Hay*

Manchester Institute of Biotechnology and Department of Chemistry,

The University of Manchester, 131 Princess Street, Manchester, M1 7DN, United Kingdom

Sam.Hay@manchester.ac.uk

Table of Contents

Characterised sequences, available X-ray crystal structures and features used in this project…..S2-S10
Figure S1
Tables S1-S3

Benchmarking of AlphaFold predictions against X-ray crystal structures……..……..…..…..….S11-S22
Tables S4-S9

Active-site visualization of AlphaFold models with X-ray crystal structures................................S23-S25
Figures S2-S3

Structural residue conservation……..……..……..……..……..……..……..……..……..…………S26–S39
Figures S4-S8
Tables S10-S11

Machine Learning workflow and classification……..……..……..……..……..……..…..………S40–S61
Figures S9-S17
Tables S12–S20

Additional References……..……..……..……..……..……..……..……..……..……..……………..S62-S69

All code, AlphaFold structures, Fpocket output and ML training and evaluation data used in this paper is freely available at https://github.com/cathaloraghallaigh/ATC.

The supplementary Excel file contains an expanded version of Table S1, including DOIs and assay conditions, together with the datasets used for training and testing all models.

Characterised sequences, available X-ray crystal structures and features used in this project

Table S1: *Experimentally characterized monoterpene synthases that produce either linalool (linear) or limonene (cyclic) as major products. Enzymes are listed with their species of origin and the publication in which their activity was described. Each enzyme produces one or more major products classified as either linear or monocyclic monoterpenes. An expanded dataset, including protein sequences, product profiles and assay conditions, is available in the GitHub repository.*

Protein ID	Category	Species	Paper Title
P0CV94	Linear	*Fragaria ananassa*	Gain and Loss of Fruit Flavor Compounds Produced by Wild and Cultivated Strawberry Species.[1]
P0CV95	Linear	*Fragaria ananassa*	Gain and Loss of Fruit Flavor Compounds Produced by Wild and Cultivated Strawberry Species.[1]
Q9M7C9	Monocyclic	*Abies grandis*	cDNA Cloning, Characterization, and Functional Expression of Four New Monoterpene Synthase Members of the Tpsd Gene Family from Grand Fir (*Abies grandis*).[2]
O22340	Monocyclic	*Abies grandis*	cDNA Cloning, Characterization, and Functional Expression of Four New Monoterpene Synthase Members of the Tpsd Gene Family from Grand Fir (*Abies grandis*).[2]
Q20HU7	Monocyclic	*Picea sitchensis*	Wound-Induced Terpene Synthase Gene Expression in *Sitka Spruce* That Exhibit Resistance or Susceptibility to Attack by the White Pine Weevil.[3]
Q84UV0	Linear	*Arabidopsis thaliana*	Biosynthesis and Emission of Terpenoid Volatiles from Arabidopsis Flowers.[4]
Q9SW76	Monocyclic	*Mentha longifolia*	Genome-Wide Analysis of Terpene Synthase Gene Family in Mentha longifolia and Catalytic Activity Analysis of a Single Terpene Synthase[5]
Q40322	Monocyclic	*Mentha spicata*	4S-limonene synthase from the oil glands of spearmint (*Mentha spicata*). cDNA isolation, characterization, and bacterial expression of the catalytically active monoterpene cyclase.[6]
Q8H2B4	Linear	*Mentha aquatica*	Molecular cloning and characterization of a new linalool synthase.[7]
F8TWD1	Linear	*Populus trichocarpa*	Four terpene synthases produce major compounds of the gypsy moth feeding-induced volatile blend of *Populus trichocarpa*.[8]
F8TWD2	Linear	*Populus trichocarpa*	Four terpene synthases produce major compounds of the gypsy moth feeding-induced volatile blend of *Populus trichocarpa*.[8]
R4YVJ5	Monocyclic	*Coffea arabica*	Functional characterization of three *Coffea arabica* L. monoterpene synthases: insights into the enzymatic machinery of coffee aroma.[9]
Q1XBU5	Linear	*Solanum lycopersicum*	The Tomato Terpene Synthase Gene Family.[10]

A0A059SVB0	Linear	*Coriandrum sativum*	Transcriptome profiling, and cloning and characterization of the main monoterpene synthases of *Coriandrum sativum* L.[11]
A7IZZ1	Monocyclic	*Cannabis sativa*	Functional Expression and Characterization of Trichome-Specific (-)-Limonene Synthase and (+)-α-Pinene Synthase from *Cannabis sativa*.[12]
ATY48639	Linear	*Phyla dulcis (Aztec sweet herb) (Lippia dulcis)*	Functional identification of a *Lippia dulcis* bornyl diphosphate synthase that contains a duplicated, inhibitory arginine-rich motif.[13]
Q5SBP3	Linear	*Ocimum basilicum*	The Biochemical and Molecular Basis for the Divergent Patterns in the Biosynthesis of Terpenes and Phenylpropenes in the Peltate Glands of Three Cultivars of Basil.[14]
Q9SPN1	Linear	*Artemisia annua*	(3R)-Linalool Synthase from *Artemisia annua* L.: cDNA Isolation, Characterization, and Wound Induction.[15]
Q9SPN0	Linear	*Artemisia annua*	(3R)-Linalool Synthase from *Artemisia annua* L.: cDNA Isolation, Characterization, and Wound Induction.[15]
C0PPR1	Linear	*Picea sitchensis*	Transcriptome mining, functional characterization, and phylogeny of a large terpene synthase gene family in spruce (Piceaspp.).[16]
F2XF93	Linear	*Picea glauca*	Transcriptome mining, functional characterization, and phylogeny of a large terpene synthase gene family in spruce (Piceaspp.).[16]
F2XFA6	Linear	*Picea sitchensis*	Transcriptome mining, functional characterization, and phylogeny of a large terpene synthase gene family in spruce (Piceaspp.).[16]
Q2XSC5	Linear	*Lavandula angustifolia*	Cloning and functional characterization of three terpene synthases from lavender (*Lavandula angustifolia*).[17]
Q2XSC6	Monocyclic	*Lavandula angustifolia*	Cloning and functional characterization of three terpene synthases from lavender (*Lavandula angustifolia*).[17]
J7HWK5	Linear	*Cinnamomum osmophloeum*	Characterization of S-(+)-linalool synthase from several provenances of *Cinnamomum osmophloeum*.[18]
A0A6C0M6B5	Linear	*Albizia julibrissin*	Five TPSs are responsible for volatile terpenoid biosynthesis in *Albizia julibrissin*.[19]
Q8L5K1	Monocyclic	*Citrus limon*	Monoterpene biosynthesis in lemon (*Citrus limon*).[20]
Q8L5K3	Monocyclic	*Citrus limon*	Monoterpene biosynthesis in lemon (*Citrus limon*).[20]
Q675L1	Monocyclic	*Picea abies*	Functional Characterization of Nine Norway Spruce TPS Genes and Evolution of Gymnosperm Terpene Synthases of the TPS-d Subfamily.[21]

Q675L2	Linear	*Picea abies*	Functional Characterization of Nine Norway Spruce TPS Genes and Evolution of Gymnosperm Terpene Synthases of the TPS-d Subfamily.[21]
Q9FUW5	Monocyclic	*Schizonepeta tenuifolia*	Molecular Cloning, Functional Expression and Characterization of d-Limonene Synthase from *Schizonepeta tenuifolia*.[22]
Q940E7	Monocyclic	*Agastache rugosa*	Molecular Cloning, Functional Expression and Characterization of d-Limonene Synthase from *Schizonepeta tenuifolia*.[22]
C0KWV5	Linear	*Perilla frutescens var. hirtella*	Geraniol and linalool synthases from wild species of perilla.[23]
C0KWV7	Linear	*Perilla frutescens var. hirtella*	Geraniol and linalool synthases from wild species of perilla.[23]
A0A1C9J6A7	Monocyclic	*Citrus sinensis*	Functional and Structural Characterization of a (+)-Limonene Synthase from *Citrus sinensis*.[24]
B1NA83	Linear	*Antirrhinum majus*	Two nearly identical terpene synthases catalyze the formation of nerolidol and linalool in snapdragon flowers.[25]
B1NA84	Linear	*Antirrhinum majus*	Two nearly identical terpene synthases catalyze the formation of nerolidol and linalool in snapdragon flowers.[25]
A0A5B9G8E4	Linear	*Prunus dulcis*	Profiling of volatile terpenes from almond (*Prunus dulcis*) young fruits and characterization of seven terpene synthase genes.[26]
B0F4G4	Monocyclic	*Mentha piperita*	Characterization and mechanism of (4S)-limonene synthase, A monoterpene cyclase from the glandular trichomes of peppermint (*Mentha x piperita*).[27]
Q6F5H2	Monocyclic	*Citrus unshiu*	Isolation and characterization of a new d-limonene synthase gene with a different expression pattern in *Citrus unshiu* Marc.[28]
Q6F5H3	Monocyclic	*Citrus unshiu*	Isolation and characterization of a new d-limonene synthase gene with a different expression pattern in *Citrus unshiu* Marc.[28]
A0A097ZLN9	Linear	*Citrus unshiu*	Isolation and characterization of a new d-limonene synthase gene with a different expression pattern in *Citrus unshiu* Marc.[28]
A0A097ZLP5	Linear	*Citrus unshiu*	Isolation and characterization of a new d-limonene synthase gene with a different expression pattern in *Citrus unshiu* Marc.[28]
A0A097ZLS9	Linear	*Citrus unshiu*	Isolation and characterization of a new d-limonene synthase gene with a different expression pattern in *Citrus unshiu* Marc.[28]
B6F137	Linear	*Backhousia citriodora*	Molecular Cloning and Characterization of a Linalool Synthase from Lemon Myrtle.[29]

Q6ZH94	Linear	*Oryza sativa subsp. japonica*	Molecular and genomic basis of volatile-mediated indirect defense against insects in rice.[30]
O04806	Monocyclic	*Perilla frutescens var. acuta*	cDNA Cloning, Characterization, and Functional Expression of 4S-(−)-Limonene Synthase from *Perilla frutescens*.[31]
U5PZT6	Linear	*Hedychium coronarium*	Characterization of two monoterpene synthases involved in floral scent formation in *Hedychium coronarium*.[32]
A0A4Y5QVX4	Monocyclic	*Cannabis sativa*	Functional Expression and Characterization of Trichome-Specific (-)-Limonene Synthase and (+)-α-Pinene Synthase from *Cannabis sativa*.[12]
A0A6P6W6H5	Monocyclic	*Coffea arabica*	Functional characterization of three *Coffea arabica* L. monoterpene synthases: insights into the enzymatic machinery of coffee aroma.[9]
D5SL78	Linear	*Streptomyces clavuligerus*	Structural basis of catalysis in the bacterial monoterpene synthases linalool synthase and 1,8-cineole synthase.[33]
Q96376	Linear	*Clarkia breweri*	Evolution of floral scent in Clarkia: novel patterns of S-linalool synthase gene expression in the *C. breweri* flower.[34]
Q9AXM7	Monocyclic	*Perilla frutescens var. frutescens*	cDNA Cloning, Characterization, and Functional Expression of 4S-(−)-Limonene Synthase from *Perilla frutescens*.[31]
A0A068B0N9	Linear	*Prunus campanulata*	Cloning and characterization of terpene synthase genes from Taiwan cherry.[35]
A0A0D3ML99	Monocyclic	*Eleutherococcus trifoliatus*	Cloning and functional characterization of a monoterpene synthase gene from *Eleutherococcus trifoliatus*.[36]
A0A0F6PMD0	Monocyclic	*Perilla frutescens*	Capturing of the monoterpene olefin limonene produced in *Saccharomyces cerevisiae*.[37]
A0A140KFH3	Linear	*Camellia saluenensis*	Identification of a novel hedycaryol synthase gene isolated from *Camellia brevistyla* flowers and floral scent of Camellia cultivars.[38]
A0A1Q1N939	Linear	*Freesia hybrid cultivar*	MYB21 interacts with MYC2 to control the expression of terpene synthase genes in flowers of *Freesia hybrida* and *Arabidopsis thaliana*.[39]
A0A2R3ZE38	Linear	*Rosa chinensis*	Linalool and linalool nerolidol synthases in roses, several genes for little scent.[40]
A0A2R3ZE39	Linear	*Rosa chinensis*	Linalool and linalool nerolidol synthases in roses, several genes for little scent.[40]
A0A345BJ04	Linear	*Lavandula x intermedia*	RNA-Seq in the discovery of a sparsely expressed scent-determining monoterpene synthase in lavender (*Lavandula*).[41]
A0A3G9EWY9	Linear	*Chengiopanax sciadophylloides*	Isolation and Functional Characterization of New Terpene Synthase Genes from Traditional Edible Plants.[42]

A0A481YAH7	Monocyclic	*Citrus japonica (Kumquat) (Fortunella crassifolia)*	Genetic diversity of limonene synthase genes in Rongan kumquat (*Fortunella crassifolia*).[43]
A0A4Y5QWA6	Linear	*Cannabis sativa*	Gene Networks Underlying Cannabinoid and Terpenoid Accumulation in Cannabis.[44]
A0A4Y5QZ62	Linear	*Cannabis sativa*	Gene Networks Underlying Cannabinoid and Terpenoid Accumulation in Cannabis.[44]
A0A7G8EIM4	Linear	*Camellia sinensis*	Enzyme Catalytic Efficiencies and Relative Gene Expression Levels of (R)-Linalool Synthase and (S)-Linalool Synthase Determine the Proportion of Linalool Enantiomers in *Camellia sinensis var. sinensis*.[45]
A0A7L7S5P2	Linear	*Ficus carica*	Characterization of terpene synthase genes potentially involved in black fig fly (*Silba adipata*) interactions with *Ficus carica*.[46]
A0A7L7S5U1	Linear	*Ficus carica*	Characterization of terpene synthase genes potentially involved in black fig fly (*Silba adipata*) interactions with *Ficus carica*.[46]
A0A7L7T499	Linear	*Wurfbainia longiligularis*	Chromosome-level genome assembly and functional characterization of terpene synthases provide insights into the volatile terpenoid biosynthesis of *Wurfbainia villosa*.[47]
A0A7L7TAQ2	Linear	*Wurfbainia villosa*	Chromosome-level genome assembly and functional characterization of terpene synthases provide insights into the volatile terpenoid biosynthesis of *Wurfbainia villosa*.[47]
C0KY88	Linear	*Osmanthus fragrans var. thunbergii*	Isolation and Expression Analysis of OfLIS Gene in *Osmanthus fragrans*.[48]
D4N3A0	Linear	*Actinidia arguta*	Characterisation of an (S)-linalool synthase from kiwifruit (*Actinidia arguta*) that catalyses the first committed step in the production of floral lilac compounds.[49]
D4N3A1	Linear	*Actinidia polygama*	Characterisation of an (S)-linalool synthase from kiwifruit (*Actinidia arguta*) that catalyses the first committed step in the production of floral lilac compounds.[49]
G5CV39	Linear	*Solanum lycopersicum*	Tomato terpene synthases TPS5 and TPS39 account for a monoterpene linalool production in tomato fruits.[49]
H6UQ81	Linear	*Rosa rugosa*	Flowery odor formation revealed by differential expression of monoterpene biosynthetic genes and monoterpene accumulation in rose (*Rosa rugosa Thunb*.).[50]
H6WBC5	Linear	*Vitis vinifera*	Identification of a Plastid-Localized Bifunctional Nerolidol/Linalool Synthase in Relation to Linalool Biosynthesis in Young Grape Berries.[51]

Q9M5A4	Monocyclic	*Perilla frutescens var. hirtella*	*Perilla citriodora* from Taiwan and Its Phytochemical Characteristics.[52]
R4I6X2	Linear	*Cinnamomum osmophloeum*	Characterization of S-(+)-linalool synthase from several provenances of *Cinnamomum osmophloeum*.[18]
dinctg000_np1212847	Linear	*Dipterocarpus intricatus*	Cyclization mechanism of monoterpenes catalyzed by monoterpene synthases in dipterocarpaceae.[53]
hmoChr44410	Linear	*Hopea mollissima*	Cyclization mechanism of monoterpenes catalyzed by monoterpene synthases in dipterocarpaceae.[53]
slectg065_np871	Linear	*Shorea leprosula*	Cyclization mechanism of monoterpenes catalyzed by monoterpene synthases in dipterocarpaceae.[53]
slectg127_np665	Linear	*Shorea leprosula*	Cyclization mechanism of monoterpenes catalyzed by monoterpene synthases in dipterocarpaceae.[18]
slectg240_np1756	Linear	*Shorea leprosula*	Cyclization mechanism of monoterpenes catalyzed by monoterpene synthases in dipterocarpaceae.[53]
vodctg000225_np551212.245	Monocyclic	*Vatica odorata*	Cyclization mechanism of monoterpenes catalyzed by monoterpene synthases in dipterocarpaceae.[53]
vodctg000225_np551212959	Linear	*Vatica odorata*	Cyclization mechanism of monoterpenes catalyzed by monoterpene synthases in dipterocarpaceae.[53]
vodctg000245_np551212567	Monocyclic	*Vatica odorata*	Cyclization mechanism of monoterpenes catalyzed by monoterpene synthases in dipterocarpaceae.[53]
vraChr51716	Monocyclic	*Vatica rassak*	Cyclization mechanism of monoterpenes catalyzed by monoterpene synthases in dipterocarpaceae.[53]
vraChr52544	Monocyclic	*Vatica rassak*	Cyclization mechanism of monoterpenes catalyzed by monoterpene synthases in dipterocarpaceae.[53]

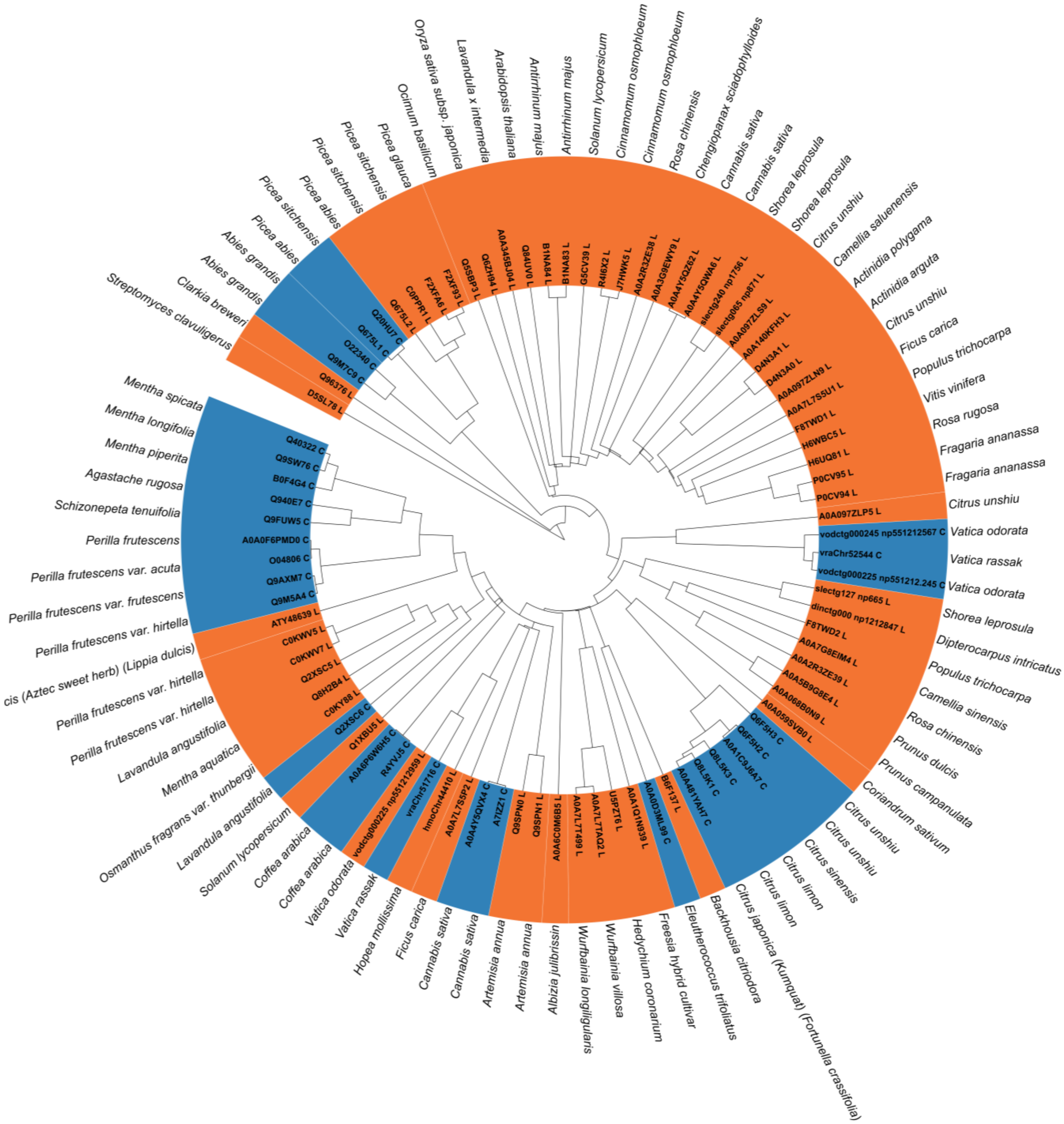

(continued on next page)

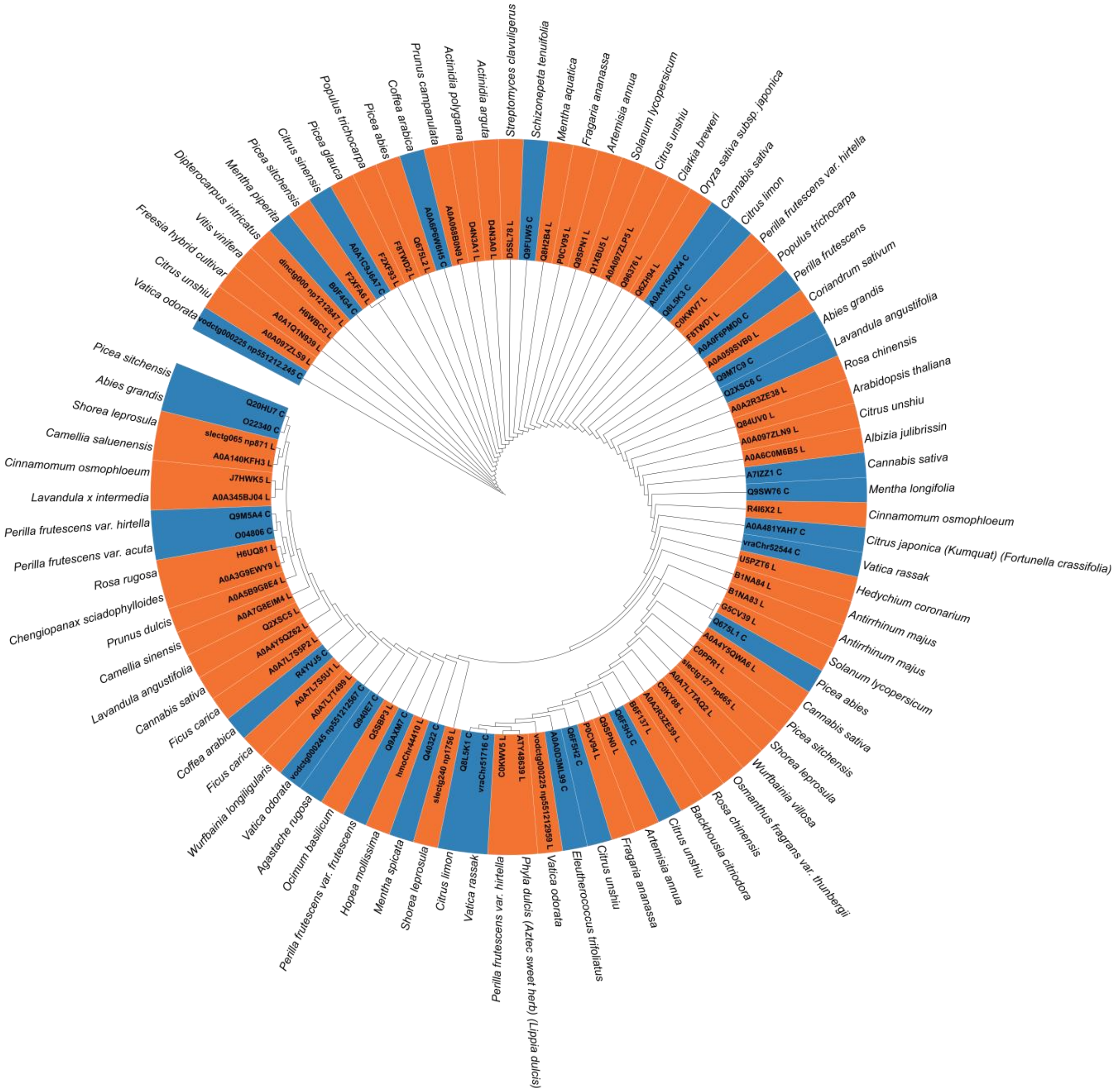

Figure S1: Phylogenetic analysis of the 88 monoterpene synthases (mTSs), with tip labels showing the UniProt accession and species name. Enzymes are colored by product preference: linalool (linear, orange) and limonene (cyclic, blue). (A) Sequence-based tree generated from a Clustal Omega multiple sequence alignment. (B) Structure-based tree constructed from pairwise structural alignments obtained using ColabAlign. Both trees were visualized using the interactive Tree of Life (iTOL) software[54].

Table S2: *Variables included in full feature set. How the variables associated with pocket characteristics are generated can be found in the Fpocket source paper,*[55] *those measuring amino acid properties (AA) are from published literature.*[56]

Cyclic	Polarity Score	Number of residues	Charge ratio
Drug Score	Amino Acid based volume Score	Ionizable groups (AA)	Uncharged (AA)
Number of alpha spheres	Pocket volume Monte Carlo	Polar (AA)	Aromatic (AA)
Mean alpha sphere radius	Pocket volume Convex hull	Non polar	Hydrophobic (AA)
Mean alpha sphere solvent acc	Charge Score	Charged (AA)	Small (AA)
Mean B factor of pocket residues	Number of apolar alpha sphere	Positive charge (AA)	Tiny (AA)
Hydrophobicity Score	Proportion of apolar alpha sphere	Negative charge (AA)	

Table S3: *List of all available X-ray crystal structures for the limonene and linalool groups, their associated uniprot IDs and their origin species.*

**UniProt ID**	**Product**	**Structure**	**Species**
**A0A1C9J6A7**	Limonene	5UV0, 5UV1, 5UV2, 6ONM	*Citrus sinensis*
**A7IZZ1**	Limonene	9OPS	*Cannabis sativa*
**Q40322**	Limonene	2ONG	*Mentha spicata*
**D5SL78**	Linalool	5NX4, 5NX5	*Streptomyces clavuligerus*
**A0A8H4VHP2** [a]	Linalool	8GY0	*Aureobasidium pediades*

[a]This enzyme (A0A8H4VHP2) was excluded from our analysis as it was identified as an outlier in the dataset as discussed in Table S7.

Benchmarking of AlphaFold predictions against X-ray crystal structures

Table S4A: *Alignment of X-ray crystal structures with their associated AlphaFold predictions for Q40322 (UniProt ID). "BL" indicates AlphaFold predictions generated with the corresponding X-ray crystal structure template excluded ("blacklisted") from the modelling process, thereby testing the ability of AlphaFold to predict the structure without relying on that experimentally determined template and assessing generalizability. (top) TM-align scores, a length- and topology-weighted measure of structural similarity, with values closer to 1.0 indicating nearly identical folds. (bottom) Root mean square distance (RMSD) values, where lower numbers indicate closer alignment.*

**TM Score**	**2ONGA**	**2ONGB**	**Q40322**	**Q40322-BL**
**2ONGA**	1	0.99445	0.9948	0.99478
**2ONGB**	0.99445	1	0.99374	0.99369
**Q40322**	0.9948	0.99374	1	0.98421
**Q40322-BL**	0.99478	0.99369	0.98421	1

**RMSD**	**2ONGA**	**2ONGB**	**Q40322**	**Q40322-BL**
**2ONGA**	0	0.65	0.62	0.62
**2ONGB**	0.65	0	0.68	0.68
**Q40322**	0.62	0.68	0	1.17
**Q40322-BL**	0.62	0.68	1.17	0

Table S4B: *Alignment of X-ray crystal structures with their associated AlphaFold predictions for Q40322 (UniProt ID), evaluated at the active site following global structural alignment to ensure fold-level superposition prior to local comparison. The active site was defined by residues identified by Fpocket. "BL" indicates AlphaFold predictions generated with the corresponding X-ray crystal structure template excluded ("blacklisted") from the modelling process, thereby testing the ability of AlphaFold to predict the structure without relying on that experimentally determined template and assessing generalizability. Root mean square distance (RMSD) values are reported, where lower numbers indicate closer structural agreement within the active site. (Top) Heavy-atom active-site RMSD calculated using the intersection of shared non-hydrogen atoms across selected residues, capturing both backbone and side-chain positional differences. (Bottom) Cα active-site RMSD calculated across the same residue set, providing a backbone-focused measure of alignment. The difference between heavy-atom and Cα RMSD reflects local side-chain displacement and fine-scale active-site variation beyond backbone movement, thereby benchmarking active-site structural conservation between experimental and predicted structures.*

	**2ONG**	**2ONGB**	**Q40322**	**Q40322-BL**
**2ONG**	0	1.423	1.669	1.672
**2ONGB**	1.423	0	1.813	1.82
**Q40322**	1.669	1.813	0	0.661
**Q40322-BL**	1.672	1.82	0.661	0

	**2ONG**	**2ONGB**	**Q40322**	**Q40322-BL**
**2ONG**	0	1.003	1.155	1.156
**2ONGB**	1.003	0	0.835	0.833
**Q40322**	1.155	0.835	0	0.677
**Q40322-BL**	1.156	0.833	0.677	0

Table S4C: Comparison of active-site residue RMSDs for Q40322 between AlphaFold2 monomer and multimer predictions. The top matrix reports all heavy-atom RMSDs, while the bottom matrix reports Cα RMSDs. For monomer predictions, the relaxed and unrelaxed structures correspond to the model with the highest pLDDT score (here, AlphaFold model 2). Multimer predictions are shown for the highest-scoring relaxed model (AlphaFold model 3), and in cases where a different AlphaFold model produced the highest-scoring multimer, this model was included for comparison.

Q40322	Relaxed (2)	Unrelaxed (2)	Multi Relaxed (2)	Multi Relaxed (3)
**Relaxed (2)**	0	0.215	1.607	1.174
**Unrelaxed (2)**	0.215	0	1.602	1.165
**Multi Relaxed (2)**	1.607	1.602	0	0.465
**Multi Relaxed (3)**	1.174	1.165	0.465	0

Q40322	Relaxed (2)	Unrelaxed (2)	Multi Relaxed (2)	Multi Relaxed (3)
**Relaxed (2)**	0	0.095	1.289	0.73
**Unrelaxed (2)**	0.095	0	1.305	0.747
**Multi Relaxed (2)**	1.289	1.305	0	0.414
**Multi Relaxed (3)**	0.73	0.747	0.414	0

Table S5A: *Alignment of X-ray crystal structures with their associated AlphaFold predictions for A0A1C9J6A7 (UniProt ID). "BL" indicates AlphaFold predictions generated with the corresponding X-ray crystal structure template excluded ("blacklisted") from the modelling process, thereby testing the ability of AlphaFold to predict the structure without relying on that experimentally determined template and assessing generalizability. (top) TM-align scores, a length- and topology-weighted measure of structural similarity, with values closer to 1.0 indicating nearly identical folds. (bottom) Root mean square distance (RMSD) values, where lower numbers indicate closer alignment.*

**TM Score**	**5UV0**	**5UV1**	**5UV2**	**6ONM**	**A0A1C9J6A7**	**A0A1C9J6A7-BL**
**5UV0**	1	0.99865	0.99909	0.99888	0.97459	0.97431
**5UV1**	0.99865	1	0.99921	0.99937	0.98355	0.98431
**5UV2**	0.99909	0.99921	1	0.99923	0.97959	0.97966
**6ONM**	0.99888	0.99937	0.99923	1	0.98711	0.98841
**A0A1C9J6A7**	0.97459	0.98355	0.97959	0.98711	1	0.95538
**A0A1C9J6A7-BL**	0.97431	0.98431	0.97966	0.98841	0.95538	1

**RMSD**	**5UV0**	**5UV1**	**5UV2**	**6ONM**	**A0A1C9J6A7**	**A0A1C9J6A7-BL**
**5UV0**	0	0.29	0.24	0.27	1.53	1.55
**5UV1**	0.29	0	0.23	0.2	1.09	1.08
**5UV2**	0.24	0.23	0	0.22	1.26	1.28
**6ONM**	0.27	0.2	0.22	0	0.92	0.88
**A0A1C9J6A7**	1.53	1.09	1.26	0.92	0	2.05
**A0A1C9J6A7-BL**	1.55	1.08	1.28	0.88	2.05	0

Table S5B: *Alignment of X-ray crystal structures with their associated AlphaFold predictions for A0A1C9J6A7 (UniProt ID), evaluated at the active site following global structural alignment to ensure fold-level superposition prior to local comparison. The active site was defined by residues identified by Fpocket. "BL" indicates AlphaFold predictions generated with the corresponding X-ray crystal structure template excluded ("blacklisted") from the modelling process, thereby testing the ability of AlphaFold to predict the structure without relying on that experimentally determined template and assessing generalizability. Root mean square distance (RMSD) values are reported, where lower numbers indicate closer structural agreement within the active site. (Top) Heavy-atom active-site RMSD calculated using the intersection of shared non-hydrogen atoms across selected residues, capturing both backbone and side-chain positional differences. (Bottom) Cα active-site RMSD calculated across the same residue set, providing a backbone-focused measure of alignment. The difference between heavy-atom and Cα RMSD reflects local side-chain displacement and fine-scale active-site variation beyond backbone movement, thereby benchmarking active-site structural conservation between experimental and predicted structures. The RMSD is high between 5UV0 and A0A1C9J6A7. However, exclusion of residues corresponding to typically unresolved or disordered regions in the other crystal structures results in a reduction in active-site RMSD (heavy-atom: 2.606 Å; Cα: 1.027 Å), with a comparable decrease observed for A0A1C9J6A7-BL (heavy-atom: 2.700 Å; Cα: 1.129 Å). While these values remain relatively high, the reduction indicates that a substantial proportion of the deviation arises from poorly resolved regions rather than consistent structural differences. The remaining discrepancy is therefore attributed to conformational variation, with the 5UV0 structure representing a more open state [24] relative to the closed conformations adopted by the AlphaFold models.*

	**5UV0**	**5UV1**	**5UV2**	**6ONM**	**A0A1C9J6A7**	**A0A1C9J6A7-BL**
**5UV0**	0	0.933	1.123	0.916	4.491	4.669
**5UV1**	0.933	0	0.316	0.321	2.634	2.676
**5UV2**	1.123	0.316	0	0.393	3.061	3.239
**6ONM**	0.916	0.321	0.393	0	2.159	2.156
**A0A1C9J6A7**	4.491	2.634	3.061	2.159	0	0.9
**A0A1C9J6A7-BL**	4.669	2.676	3.239	2.156	0.9	0

	**5UV0**	**5UV1**	**5UV2**	**6ONM**	**A0A1C9J6A7**	**A0A1C9J6A7-BL**
**5UV0**	0	0.438	0.48	0.532	3.529	3.693
**5UV1**	0.438	0	0.174	0.197	1.043	1.065
**5UV2**	0.48	0.174	0	0.271	1.885	2.092
**6ONM**	0.532	0.197	0.271	0	1.063	1.065
**A0A1C9J6A7**	3.529	1.043	1.885	1.063	0	0.742
**A0A1C9J6A7-BL**	3.693	1.065	2.092	1.065	0.742	0

Table S5C: Comparison of active-site residue RMSDs for A0A1C9J6A7 between AlphaFold2 monomer and multimer predictions. The top matrix reports all heavy-atom RMSDs, while the bottom matrix reports Cα RMSDs. For monomer predictions, the relaxed and unrelaxed structures correspond to the model with the highest pLDDT score (here, AlphaFold model 4). Multimer predictions are shown for the highest-scoring relaxed model (AlphaFold model 1), and in cases where a different AlphaFold model produced the highest-scoring multimer, this model was included for comparison.

A0A1C9J6A7	Relaxed (4)	Unrelaxed (4)	Multi Relaxed (1)	Multi Relaxed (4)
**Relaxed (4)**	0	0.214	1.486	1.262
**Unrelaxed (4)**	0.214	0	1.475	1.267
**Multi Relaxed (1)**	1.486	1.475	0	1.292
**Multi Relaxed (4)**	1.262	1.267	1.292	0

A0A1C9J6A7	Relaxed (4)	Unrelaxed (4)	Multi Relaxed (1)	Multi Relaxed (4)
**Relaxed (4)**	0	0.097	0.838	1.126
**Unrelaxed (4)**	0.097	0	0.824	1.123
**Multi Relaxed (1)**	0.838	0.824	0	0.703
**Multi Relaxed (4)**	1.126	1.123	0.703	0

Table S6A: *Alignment of X-ray crystal structures with their associated AlphaFold predictions for D5SL78 (UniProt ID). "BL" indicates AlphaFold predictions generated with the corresponding X-ray crystal structure template excluded ("blacklisted") from the modelling process, thereby testing the ability of AlphaFold to predict the structure without relying on that experimentally determined template and assessing generalizability. (top) TM-align scores, a length- and topology-weighted measure of structural similarity, with values closer to 1.0 indicating nearly identical folds. (bottom) Root mean square distance (RMSD) values, where lower numbers indicate closer alignment.*

TM Score	5NX4	5NX5	D5SL78	D5SL78-BL
**5NX4**	1	0.99355	0.98787	0.98778
**5NX5**	0.99355	1	0.99015	0.98992
**D5SL78**	0.98787	0.99015	1	0.99426
**D5SL78-BL**	0.98778	0.98992	0.99426	1

RMSD	5NX4	5NX5	D5SL78	D5SL78-BL
**5NX4**	0	0.34	0.7	0.71
**5NX5**	0.34	0	0.63	0.64
**D5SL78**	0.7	0.63	0	0.52
**D5SL78-BL**	0.71	0.64	0.52	0

Table S6B: *Alignment of X-ray crystal structures with their associated AlphaFold predictions for D5SL78 (UniProt ID), evaluated at the active site following global structural alignment to ensure fold-level superposition prior to local comparison. The active site was defined by residues identified by Fpocket. "BL" indicates AlphaFold predictions generated with the corresponding X-ray crystal structure template excluded ("blacklisted") from the modelling process, thereby testing the ability of AlphaFold to predict the structure without relying on that experimentally determined template and assessing generalizability. Root mean square distance (RMSD) values are reported, where lower numbers indicate closer structural agreement within the active site. (Top) Heavy-atom active-site RMSD calculated using the intersection of shared non-hydrogen atoms across selected residues, capturing both backbone and side-chain positional differences. (Bottom) Cα active-site RMSD calculated across the same residue set, providing a backbone-focused measure of alignment. The difference between heavy-atom and Cα RMSD reflects local side-chain displacement and fine-scale active-site variation beyond backbone movement, thereby benchmarking active-site structural conservation between experimental and predicted structures.*

	5NX4	5NX5	D5SL78	D5SL78-BL
**5NX4**	0	0.579	1.114	1.078
**5NX5**	0.579	0	1.067	1.023
**D5SL78**	1.114	1.067	0	0.255
**D5SL78-BL**	1.078	1.023	0.255	0

	5NX4	5NX5	D5SL78	D5SL78-BL
**5NX4**	0	0.242	0.492	0.48
**5NX5**	0.242	0	0.447	0.436
**D5SL78**	0.492	0.447	0	0.145
**D5SL78-BL**	0.48	0.436	0.145	0

Table S6C: Comparison of active-site residue RMSDs for D5SL78 between AlphaFold2 monomer and multimer predictions. The top matrix reports all heavy-atom RMSDs, while the bottom matrix reports Cα RMSDs. For monomer predictions, the relaxed and unrelaxed structures correspond to the model with the highest pLDDT score (here, AlphaFold model 2). Multimer predictions are shown for the highest-scoring relaxed model (AlphaFold model 5), and in cases where a different AlphaFold model produced the highest-scoring multimer, this model was included for comparison.

D5SL78	Relaxed (2)	Unrelaxed (2)	Multi Relaxed (2)	Multi Relaxed (5)
**Relaxed (2)**	0	0.176	0.576	0.589
**Unrelaxed (2)**	0.176	0	0.607	0.621
**Multi Relaxed (2)**	0.576	0.607	0	0.179
**Multi Relaxed (5)**	0.589	0.621	0.179	0

D5SL78	Relaxed (2)	Unrelaxed (2)	Multi Relaxed (2)	Multi Relaxed (5)
**Relaxed (2)**	0	0.09	0.373	0.331
**Unrelaxed (2)**	0.09	0	0.407	0.361
**Multi Relaxed (2)**	0.373	0.407	0	0.144
**Multi Relaxed (5)**	0.331	0.361	0.144	0

Table S7A: *Alignment of X-ray crystal structures with their associated AlphaFold predictions for A0A8H4VHP2 (UniProt ID). "BL" indicates AlphaFold predictions generated with the corresponding X-ray crystal structure template excluded ("blacklisted") from the modelling process, thereby testing the ability of AlphaFold to predict the structure without relying on that experimentally determined template and assessing generalizability. (top) TM-align scores, a length- and topology-weighted measure of structural similarity, with values closer to 1.0 indicating nearly identical folds. (bottom) Root mean square distance (RMSD) values, where lower numbers indicate closer alignment.*

TM Score	8GY0A	8GY0B	A0A8H4VHP2	A0A8H4VHP2-BL
**8GY0A**	1	0.99545	0.90956	0.91233
**8GY0B**	0.99545	1	0.91261	0.91528
**A0A8H4VHP2**	0.90956	0.91261	1	0.97922
**A0A8H4VHP2-BL**	0.91233	0.91528	0.97922	1

RMSD	8GY0A	8GY0B	A0A8H4VHP2	A0A8H4VHP2-BL
**8GY0A**	0	0.49	2.21	2.19
**8GY0B**	0.49	0	2.13	2.1
**A0A8H4VHP2**	2.21	2.13	0	1.23
**A0A8H4VHP2-BL**	2.19	2.1	1.23	0

Table S7B: *Alignment of X-ray crystal structures with their associated AlphaFold predictions for A0A8H4VHP2 (UniProt ID), evaluated at the active site following global structural alignment to ensure fold-level superposition prior to local comparison. The active site was defined by residues identified by Fpocket. "BL" indicates AlphaFold predictions generated with the corresponding X-ray crystal structure template excluded ("blacklisted") from the modelling process, thereby testing the ability of AlphaFold to predict the structure without relying on that experimentally determined template and assessing generalizability. Root mean square distance (RMSD) values are reported, where lower numbers indicate closer structural agreement within the active site. (Top) Heavy-atom active-site RMSD calculated using the intersection of shared non-hydrogen atoms across selected residues, capturing both backbone and side-chain positional differences. (Bottom) Cα active-site RMSD calculated across the same residue set, providing a backbone-focused measure of alignment. The difference between heavy-atom and Cα RMSD reflects local side-chain displacement and fine-scale active-site variation beyond backbone movement, thereby benchmarking active-site structural conservation between experimental and predicted structures.*

	8GY0	8GY0B	A0A8H4VHP2-BL	A0A8H4VHP2
**8GY0**	0	1.687	4.909	4.851
**8GY0B**	1.687	0	4.65	4.601
**A0A8H4VHP2-BL**	4.909	4.65	0	1.78
**A0A8H4VHP2**	4.851	4.601	1.78	0

	8GY0	8GY0B	A0A8H4VHP2-BL	A0A8H4VHP2
**8GY0**	0	1.015	3.823	3.721
**8GY0B**	1.015	0	3.54	3.444
**A0A8H4VHP2-BL**	3.823	3.54	0	1.673
**A0A8H4VHP2**	3.721	3.444	1.673	0

Table S7C: Comparison of active-site residue RMSDs for A0A8H4VHP2 between AlphaFold2 monomer and multimer predictions. The top matrix reports all heavy-atom RMSDs, while the bottom matrix reports Cα RMSDs. For monomer predictions, the relaxed and unrelaxed structures correspond to the model with the highest pLDDT score (here, AlphaFold model 3). Multimer predictions are shown for the highest-scoring relaxed model (AlphaFold model 4), and in cases where a different AlphaFold model produced the highest-scoring multimer, this model was included for comparison.

A0A8H4VHP2	Relaxed (3)	Unrelaxed (3)	Multi Relaxed (3)	Multi Relaxed (4)
**Relaxed (3)**	0	0.208	1.398	0.976
**Unrelaxed (3)**	0.208	0	1.414	0.991
**Multi Relaxed (3)**	1.398	1.414	0	0.765
**Multi Relaxed (4)**	0.976	0.991	0.765	0

A0A8H4VHP2	Relaxed (3)	Unrelaxed (3)	Multi Relaxed (3)	Multi Relaxed (4)
**Relaxed (3)**	0	0.1	1.196	0.873
**Unrelaxed (3)**	0.1	0	1.206	0.88
**Multi Relaxed (3)**	1.196	1.206	0	0.472
**Multi Relaxed (4)**	0.873	0.88	0.472	0

The misalignment between the AlphaFold prediction for A0A8H4VHP2 and the corresponding crystal structure observed here is consistent with its behavior in downstream analyses. Specifically, inclusion of this structure, whether using the experimental model or the associated AlphaFold prediction, led to a consistent reduction in machine learning performance during training. As a result, A0A8H4VHP2 was excluded from our statistical analysis (**Figure 3**) and the final modelling dataset. While the precise structural origin of this discrepancy remains unclear, its impact on model performance supports the interpretation that this protein represents an outlier within the dataset. It is therefore retained here for structural benchmarking purposes only.

Table 8A: *Alignment of X-ray crystal structures with their associated AlphaFold predictions for A7IZZ1 (UniProt ID). "BL" indicates AlphaFold predictions generated with the corresponding X-ray crystal structure template excluded ("blacklisted") from the modelling process, thereby testing the ability of AlphaFold to predict the structure without relying on that experimentally determined template and assessing generalizability. (top) TM-align scores, a length- and topology-weighted measure of structural similarity, with values closer to 1.0 indicating nearly identical folds. (bottom) Root mean square distance (RMSD) values, where lower numbers indicate closer alignment.*

TM Score	9OPS	A7IZZ1	A7IZZ1-BL
**9OPS**	1	0.98811	0.98619
**A7IZZ1**	0.98811	1	0.89247
**A7IZZ1-BL**	0.98619	0.89247	1

RMSD	9OPS	A7IZZ1	A7IZZ1-BL
**9OPS**	0	0.91	1
**A7IZZ1**	0.91	0	1.62
**A7IZZ1-BL**	1	1.62	0

Table S8B: *Alignment of X-ray crystal structures with their associated AlphaFold predictions for A7IZZ1 (UniProt ID), evaluated at the active site following global structural alignment to ensure fold-level superposition prior to local comparison. The active site was defined by residues identified by Fpocket. "BL" indicates AlphaFold predictions generated with the corresponding X-ray crystal structure template excluded ("blacklisted") from the modelling process, thereby testing the ability of AlphaFold to predict the structure without relying on that experimentally determined template and assessing generalizability. Root mean square distance (RMSD) values are reported, where lower numbers indicate closer structural agreement within the active site. (Top) Heavy-atom active-site RMSD calculated using the intersection of shared non-hydrogen atoms across selected residues, capturing both backbone and side-chain positional differences. (Bottom) Cα active-site RMSD calculated across the same residue set, providing a backbone-focused measure of alignment. The difference between heavy-atom and Cα RMSD reflects local side-chain displacement and fine-scale active-site variation beyond backbone movement, thereby benchmarking active-site structural conservation between experimental and predicted structures.*

	9OPS	A7IZZ1	A7IZZ1-BL
**9OPS**	0	1.065	1.088
**A7IZZ1**	1.065	0	1
**A7IZZ1-BL**	1.088	1	0

	9OPS	A7IZZ1	A7IZZ1-BL
**9OPS**	0	0.746	0.858
**A7IZZ1**	0.746	0	0.927
**A7IZZ1-BL**	0.858	0.927	0

Table S8C: *Comparison of active-site residue RMSDs for A7IZZ1 between AlphaFold2 monomer and multimer predictions. The top matrix reports all heavy-atom RMSDs, while the bottom matrix reports Cα RMSDs. For monomer predictions, the relaxed and unrelaxed structures correspond to the model with the highest pLDDT score (here, AlphaFold model 3). Multimer predictions are shown for the highest-scoring relaxed model (AlphaFold model 1), and in cases where a different AlphaFold model produced the highest-scoring multimer, this model was included for comparison.*

**A7IZZ1**	**Relaxed (3)**	**Unrelaxed (3)**	**Multi Relaxed (1)**	**Multi Relaxed (3)**
**Relaxed (3)**	0	0.233	0.898	1.278
**Unrelaxed (3)**	0.233	0	0.946	1.299
**Multi Relaxed (1)**	0.898	0.946	0	1.234
**Multi Relaxed (3)**	1.278	1.299	1.234	0

**A7IZZ1**	**Relaxed (3)**	**Unrelaxed (3)**	**Multi Relaxed (1)**	**Multi Relaxed (3)**
**Relaxed (3)**	0	0.123	0.411	0.983
**Unrelaxed (3)**	0.123	0	0.453	0.99
**Multi Relaxed (1)**	0.411	0.453	0	0.989
**Multi Relaxed (3)**	0.983	0.99	0.989	0

Table S9A: Comparison of pocket features and classification performance between original and alternative AlphaFold2 models. Structures with pocket residue-count similarity below 75% of the original model were excluded to remove pockets affected by pinching or extension beyond the active site. Analysis included 73 enzymes with at least one remaining alternative structure, comprising 56 second-ranked and 57 third-ranked models. Similarity was calculated for amino-acid composition, all 46 extracted pocket features, and the 12 selected model features. Classification was assessed using leave-one-protein-out cross-validation with the original hyperparameters, training on the remaining original structures and testing the withheld original and alternative models.

Metric	***Original***	***Alternative 1***	***Alternative 2***	***All alternatives***
**Structures**	73	56	57	113
**Proteins**	73	56	57	73
**Mean pocket residues**	33.4	30.4	30.8	30.6
**Amino-acid composition similarity (%)**	—	87.3	85.6	86.4
**All-feature similarity (%)**	—	87.2	86.1	86.6

**FS1-feature similarity (%)**	—	88.4	87.8	88.1
**Accuracy (%)**	89	85.7	91.2	88.5
**Balanced accuracy (%)**	89	86.5	92.1	89.3
**MCC**	0.77	0.722	0.822	0.772

Table S9B: Comparison of individual FS1 features between original and alternative structural models. Values show the mean feature value for each model, the mean absolute difference between original and alternative models, and the mean similarity across all comparisons.

Feature	Original mean	Alternative 1 mean	Alternative 2 mean	Mean absolute difference	Similarity (%)
**Alanine**	1.52	1.2	1.33	0.63	68
**Methionine**	0.59	0.62	0.58	0.1	91.5
**Tryptophan**	1.15	0.98	1.07	0.19	87.9
**Isoleucine**	2.3	2.27	2.21	0.32	91.5
**Cysteine**	0.25	0.14	0.21	0.06	93.8
**Histidine**	0.58	0.62	0.51	0.24	84.2
**Asparagine**	0.84	0.88	0.68	0.27	82
**Positive charge**	4.37	4.5	4.19	1.06	87.6
**Negative charge**	7.01	6.43	6.58	1.06	91.5
**Polar**	22.27	20.57	20.58	3.48	91.8
**Non-polar**	11.4	9.93	10.42	2.24	89.2
**Amino-acid-based volume score**	4.4	4.46	4.44	0.16	98.2

Active-site visualization of AlphaFold models with X-ray crystal structures.

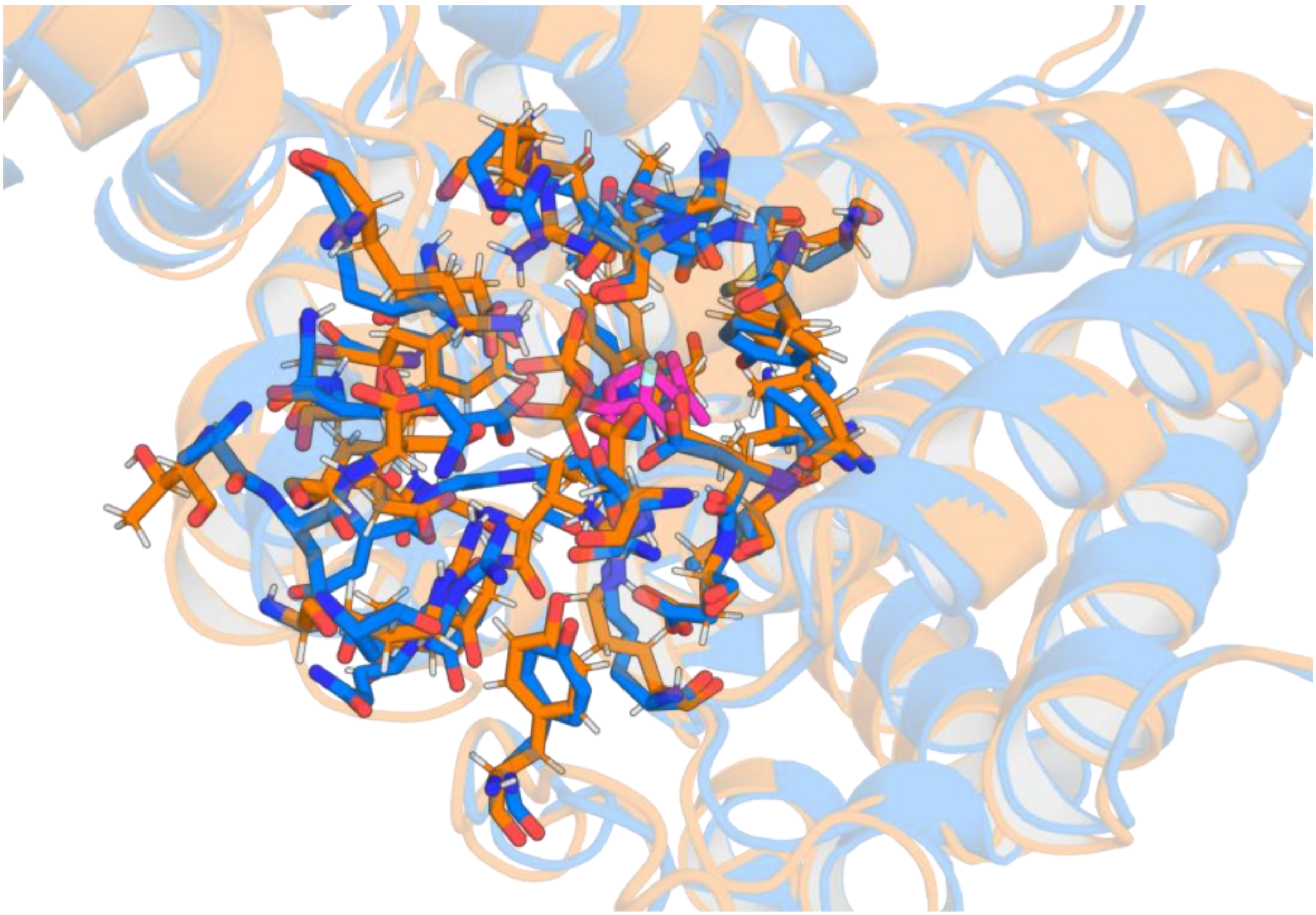

Figure S2: *Alignment of the AlphaFold2 model of F8TWD2 (member of the linear group; orange) with an X-ray crystal structure of a limonene synthase (PDB ID: 2ONG; blue). Here all residues in the active site are overlaid and the 2-fluorogeranyl diphosphate (FGPP) substrate analog found in the X-ray crystal structure is shown in magenta.*

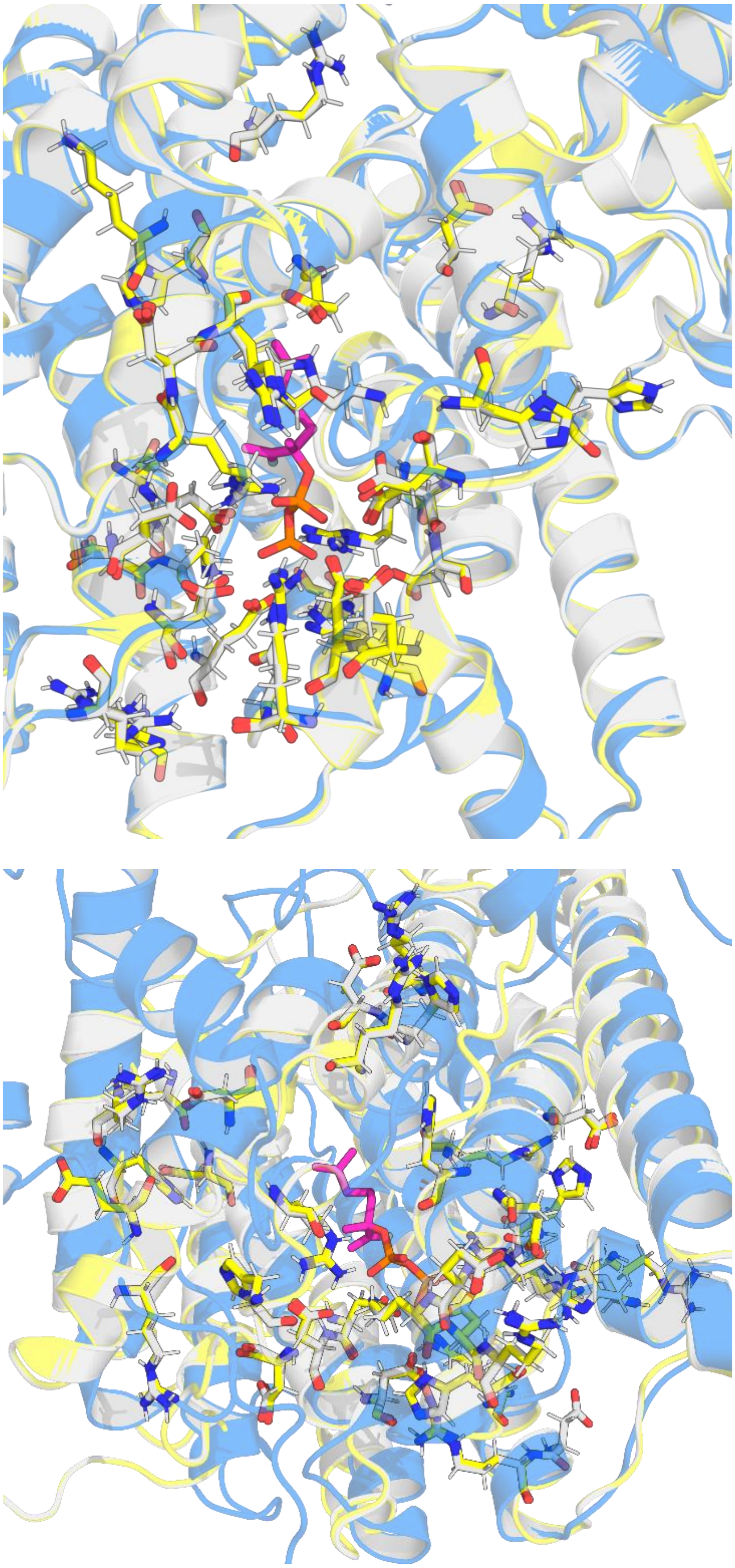

Figure S3A: *Alignment of the relaxed (grey) and unrelaxed (yellow) AlphaFold2 models of D5SL78 (top) and Q40322 (bottom) with PDB: 2ONG (blue, with FGPP shown in magenta). All ionizable residues (Asp, Glu, His, Lys, Arg) within 12 Å of the FGPP are shown. There is minimal perturbation around these residues, suggesting that the AMBER relaxation step is not improperly altering these models.*

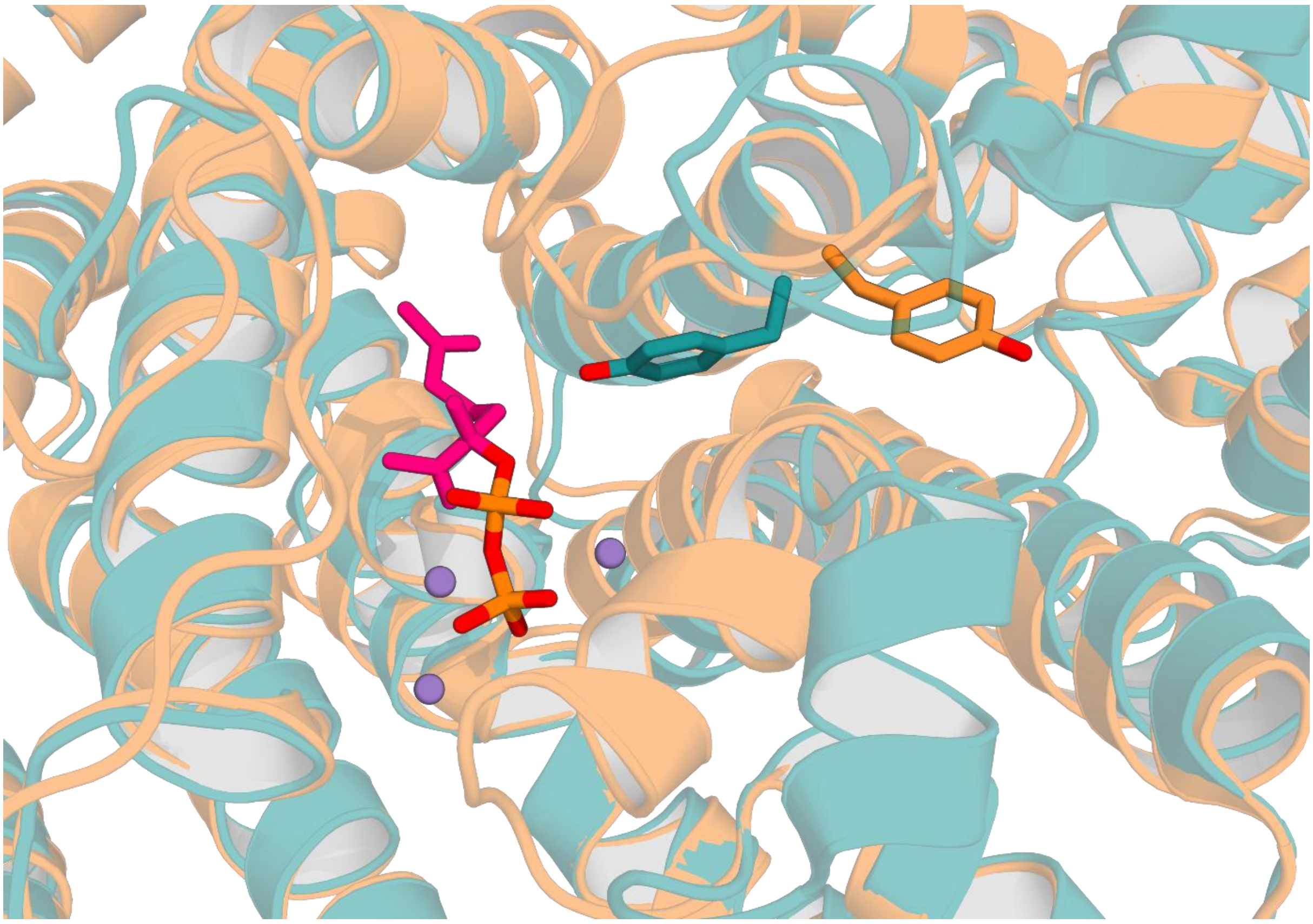

Figure S3B: *Comparison of open and closed limonene synthase conformations based on the X-ray crystal structures of (+)-limonene synthase from Citrus sinensis (PDB ID: 5UV0; UniProt: A0A1C9J6A7) and (4S)-limonene synthase from Mentha spicata (PDB ID: 2ONG; UniProt: Q40322). In this overlay, 5UV0 is shown in orange and 2ONG in mint, with the conserved active-site tyrosines Y565 in 5UV0 and Y573 in 2ONG showing as sticks, together with the 2-fluorogeranyl diphosphate (FGPP) substrate analog and three Mn2+ are shown in magenta and purple, respectively. The comparison illustrates the open versus closed structural transition, with 2ONG representing a fully closed state and 5UV0 an open state shown previously.[24] Closure involves an inward movement of the short H-α1 helix and a flip of the conserved tyrosine into the active site, making this residue movement a clear marker of the conformational difference between the two states. This comparison was used to assess the conformational state of the AlphaFold models by measuring the position of the equivalent residue relative to the 5UV0 and 2ONG reference states; the vast majority of models were classified as closed, in agreement with what has been seen in literature previously,[57] with only three of the 88 proteins, C0PPR1, F2XFA6, and Q6ZH94, were identified as open cases in this analysis.*

Structural residue conservation

Table S10: *A per-position residue frequencies for the limonene group and linalool group at the manually selected positions. Entries are reported as Residue frequency, with Δ columns giving the absolute frequency difference between groups (cyclic and linear). Only residues with group frequency ≥ 0.15 are listed, gaps were excluded (no residues within 4 Å). "Specified Residue" denotes the residue at that position in the reference sequence.*

	Residue Position	Cyclic Group	Δ Cyclic	Linear Group	Δ Linear	Specified Residue
0	63	Y:0.82	Y:Δ0.45	Y:0.37	Y:Δ0.45	Y
1	315	R:1.00	R:Δ0.02	R:0.98	R:Δ0.02	R
2	324	W:0.86	W:Δ0.04	W:0.81	W:Δ0.04	W
3	345	I:0.39, N:0.25	N:Δ0.18, I:Δ0.13	I:0.53	I:Δ0.13	N
4	348	I:0.61	I:Δ0.15	I:0.46, V:0.24	V:Δ0.20, I:Δ0.15	I
5	349	T:0.86	T:Δ0.38	T:0.47, Y:0.44	Y:Δ0.44, T:Δ0.38	T
6	352	D:1.00	D:Δ0.00	D:1.00	D:Δ0.00	D
7	353	D:1.00	D:Δ0.00	D:1.00	D:Δ0.00	D
8	356	D:1.00	D:Δ0.03	D:0.97	D:Δ0.03	D
9	427	Y:0.86	Y:Δ0.35	Y:0.51, F:0.44	F:Δ0.37, Y:Δ0.35	Y
10	430	E:1.00	E:Δ0.02	E:0.98	E:Δ0.02	E
11	452	S:1.00	S:Δ0.12	S:0.88	S:Δ0.12	S
12	453	I:0.50, S:0.25, V:0.18	S:Δ0.17, I:Δ0.13, V:Δ0.08	S:0.42, I:0.37	S:Δ0.17, I:Δ0.13	I
13	454	S:0.39, G:0.29, T:0.21	G:Δ0.51, S:Δ0.27, T:Δ0.20	G:0.80	G:Δ0.51	S
14	458	M:0.46, I:0.29	M:Δ0.45, I:Δ0.18	V:0.49, A:0.22	V:Δ0.38, A:Δ0.08	M
15	492	L:0.61, F:0.21	L:Δ0.17, F:Δ0.08	L:0.44	L:Δ0.17	L
16	493	R:0.75	R:Δ0.01	R:0.76, L:0.19	L:Δ0.04, R:Δ0.01	R
17	496	D:0.82	D:Δ0.02	D:0.80	D:Δ0.02	D
18	499	G:0.75	G:Δ0.04	G:0.71	G:Δ0.04	G
19	500	T:0.75	T:Δ0.28	T:0.47, A:0.29	A:Δ0.29, T:Δ0.28	T
20	502	S:0.32, K:0.25, L:0.21	L:Δ0.15, K:Δ0.10, S:Δ0.09	S:0.41, K:0.15	K:Δ0.10, S:Δ0.09	V

	Residue Position	Cyclic Group	Δ Cyclic	Linear Group	Δ Linear	Specified Residue
21	503	E:0.61	E:Δ0.42	K:0.41, E:0.19, A:0.17	E:Δ0.42, K:Δ0.41, A:Δ0.03	E
22	504	E:0.86	E:Δ0.33	E:0.53, D:0.15	E:Δ0.33, D:Δ0.01	E
23	507	R:1.00	R:Δ0.47	R:0.53, D:0.15	R:Δ0.47, D:Δ0.15	R
24	509	D:0.75	D:Δ0.02	D:0.73	D:Δ0.02	D
25	512	K:0.75, S:0.25	K:Δ0.36, S:Δ0.29	S:0.54, K:0.39	K:Δ0.36, S:Δ0.29	K
26	573	Y:1.00	Y:Δ0.19	Y:0.81	Y:Δ0.19	Y
27	577	D:0.75, G:0.25	D:Δ0.41, G:Δ0.18	D:0.34, Y:0.20	D:Δ0.41, Y:Δ0.20	D
28	578	G:0.50, Y:0.18	G:Δ0.28, Y:Δ0.14	G:0.22	G:Δ0.28	G
29	579	H:0.71, Y:0.18	H:Δ0.68, Y:Δ0.08			H
30	581	G:0.46, T:0.18	G:Δ0.28, T:Δ0.16	G:0.19	G:Δ0.28	T
31	582	V:0.25, Q:0.21	Q:Δ0.20, V:Δ0.13			Q

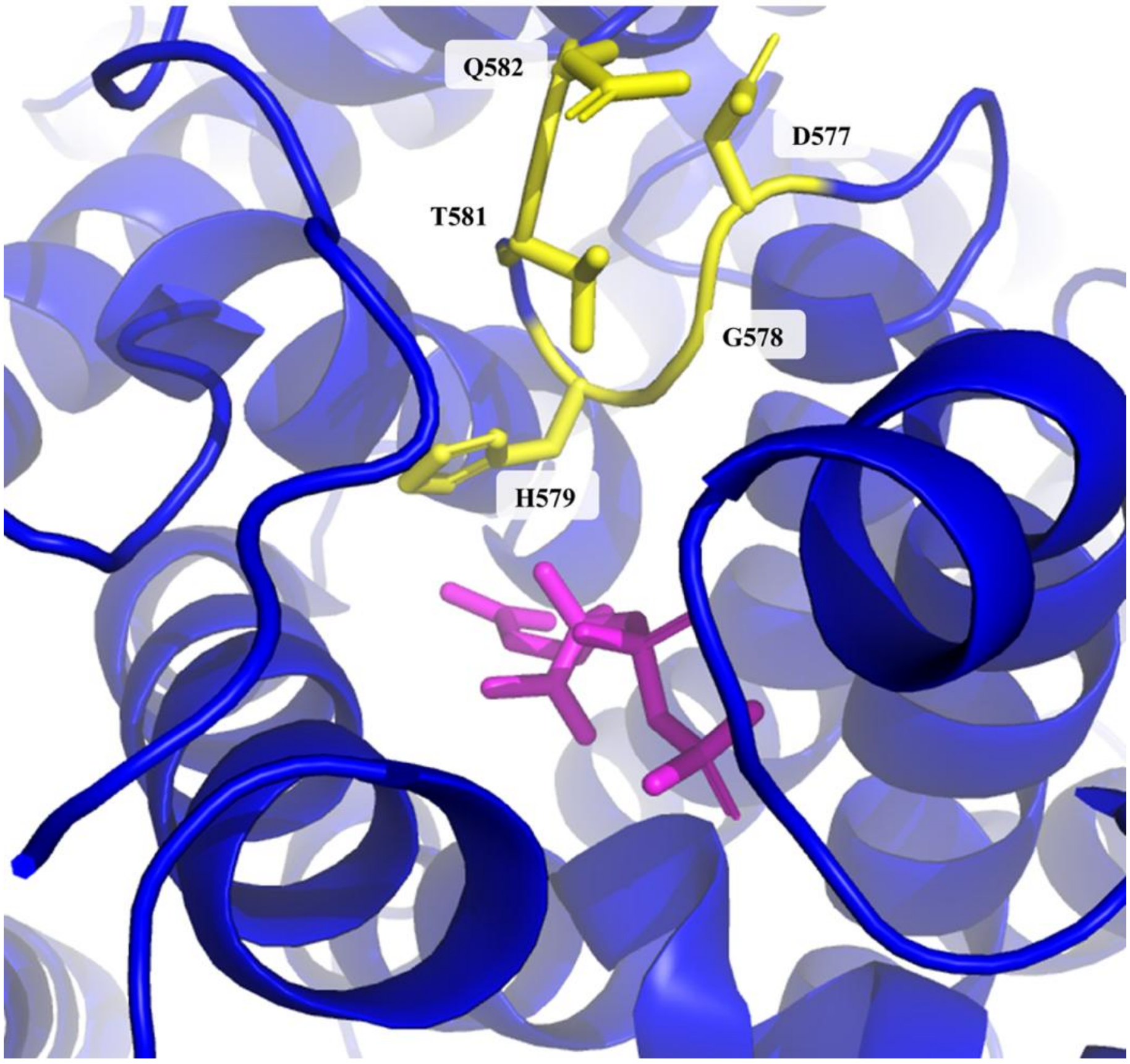

Figure S4: *Active site view of Citrus limon limonene synthase (PDB ID: 2ONG). Geranyl pyrophosphate (GPP) is shown in pink, and the residues identified as "missing" from the linear group are highlighted in yellow. These residues are classified as missing because no corresponding residues in the linear-producing enzymes are located within 4 Å of the substrate.*

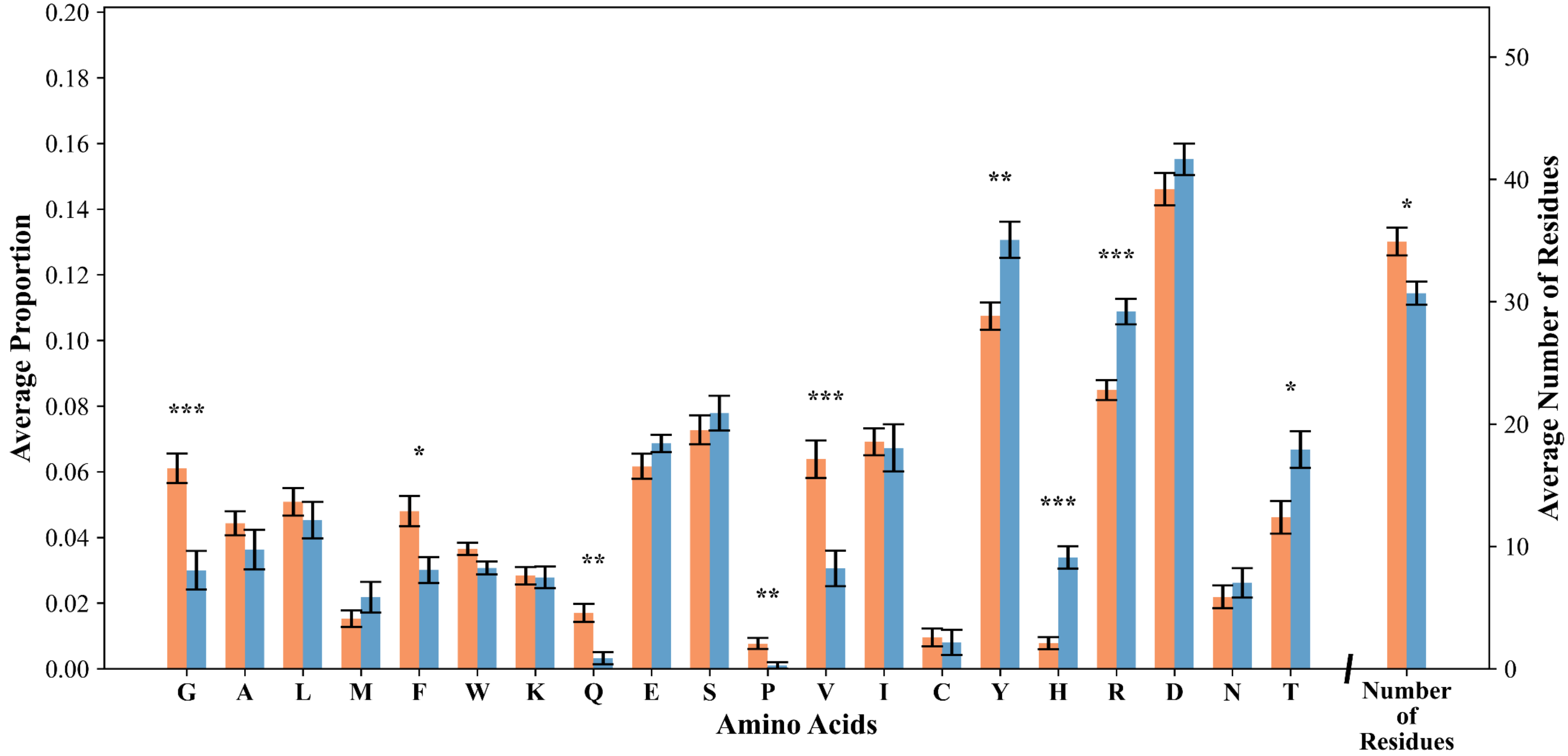

(continued on next page)

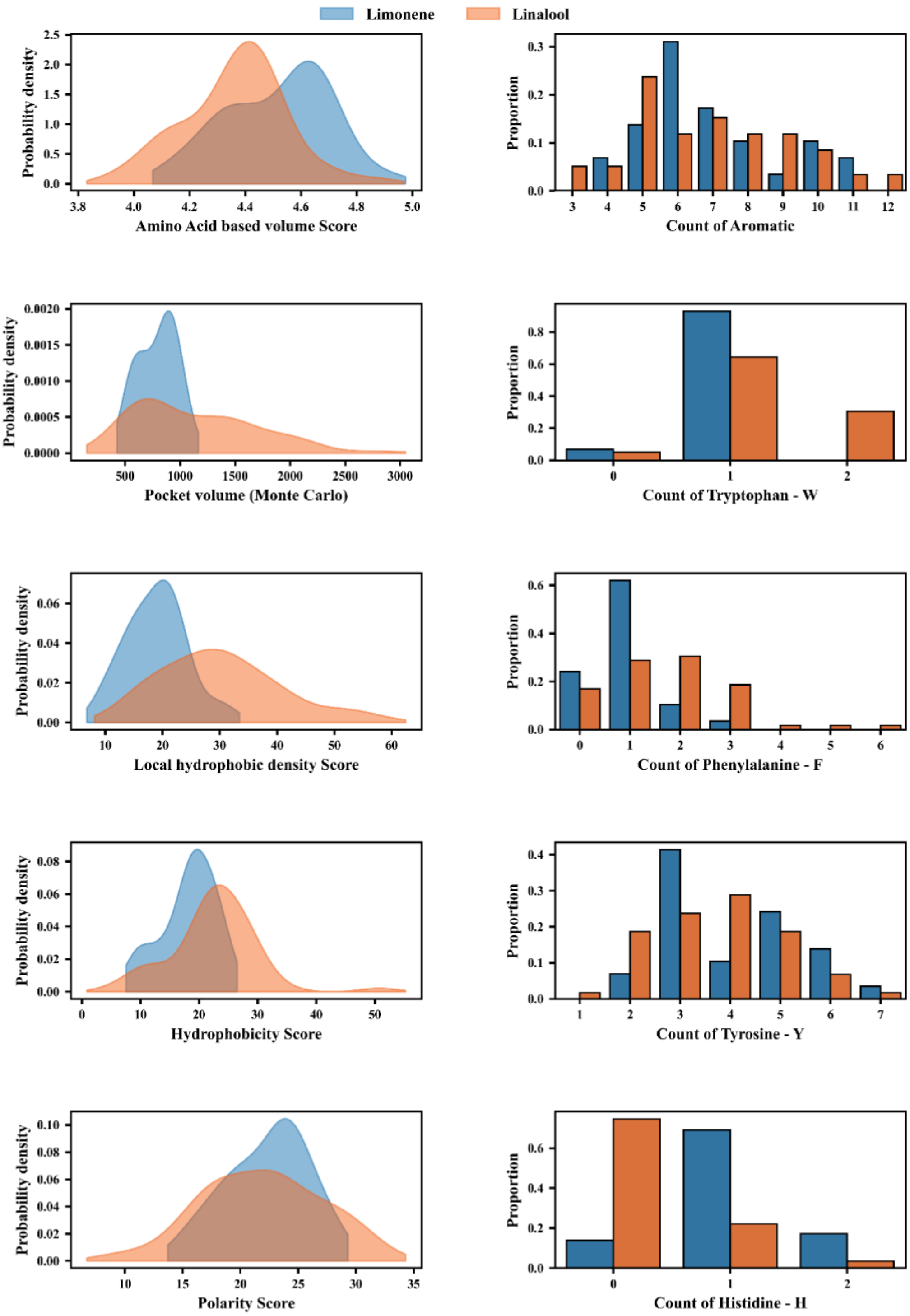

Figure S5: Enlarged version of Figure 3 showing amino acid frequencies and residue counts for linear (orange) and cyclic (blue) monoterpene synthases (top), with distributions of pocket properties and aromatic residue frequencies shown below.

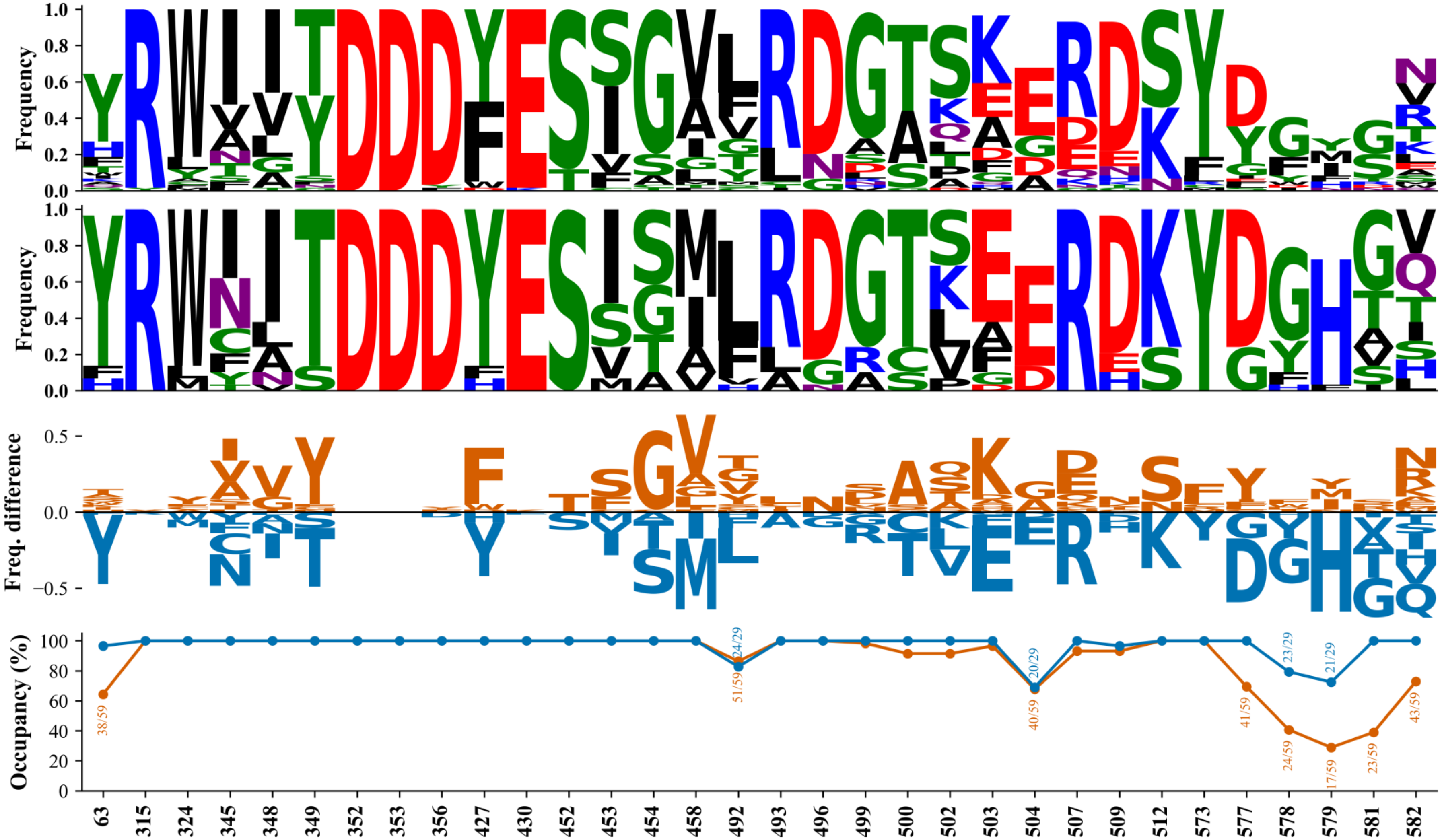

Figure S6A: *Active-site sequence conservation determined using the limonene synthase crystal structure 2ONG as the reference. Sequence logos are shown for linear (top) and cyclic (upper middle) enzymes, followed by the difference sequence logo (lower middle) and residue occupancy at each reference position (bottom). Active-site residues were identified using the nearest-residue structural alignment procedure described in the main text.*

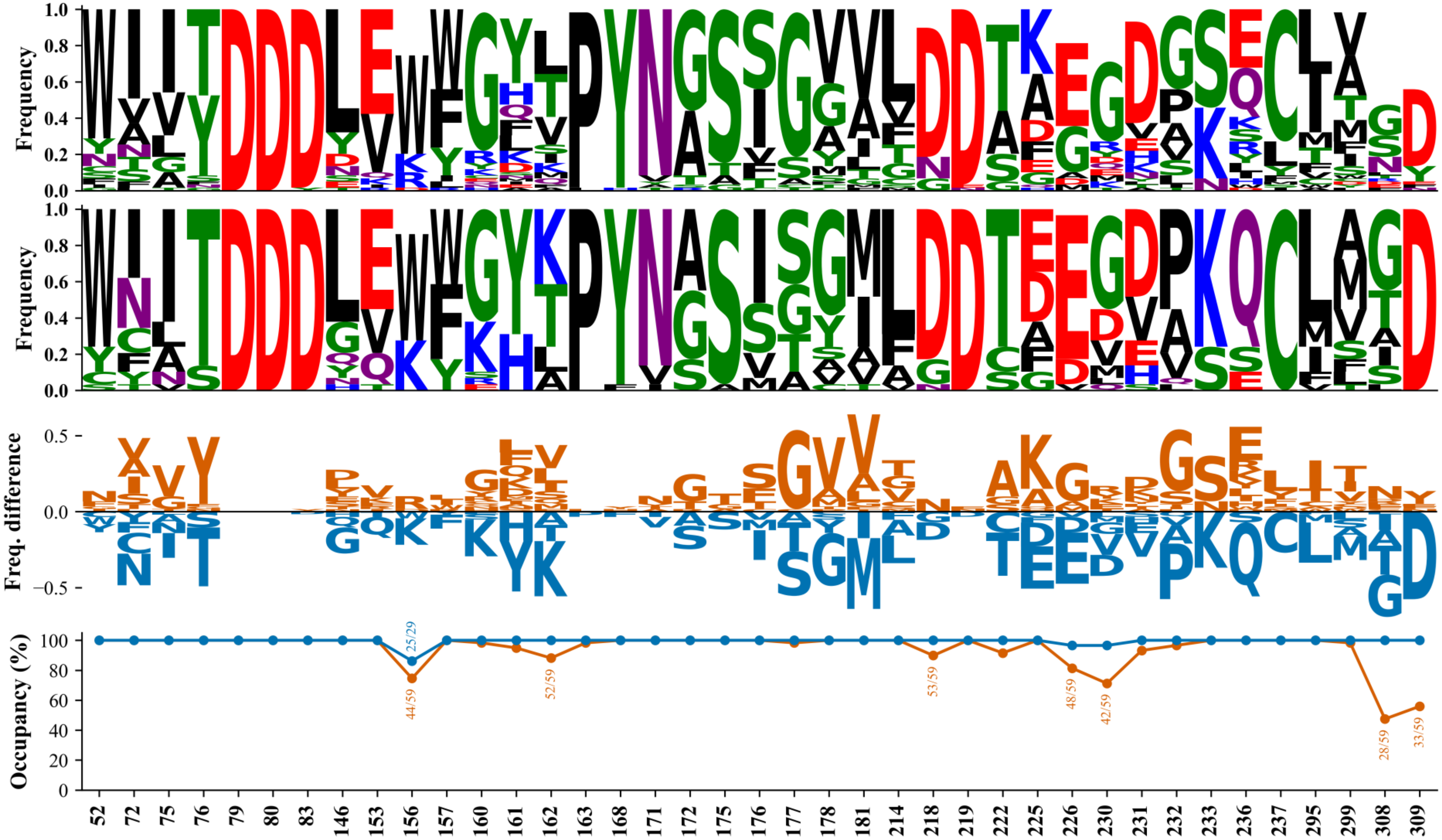

Figure S6B: *Active-site sequence conservation determined using the linalool synthase crystal structure 5NX5 as the reference. Sequence logos are shown for linear (top) and cyclic (upper middle) enzymes, followed by the difference sequence logo (lower middle) and residue occupancy at each reference position (bottom). Active-site residues were identified using the nearest-residue structural alignment procedure described in the main text.Broadly similar conservation patterns are observed relative to the 2ONG-based analysis (Figure 4). Differences in residue frequency at certain positions are present, the most noticeable being the final aspartate residue (83) in the DDxxD motif. However, this is likely due to the X-ray crystal structure being poorly resolved at this position.*

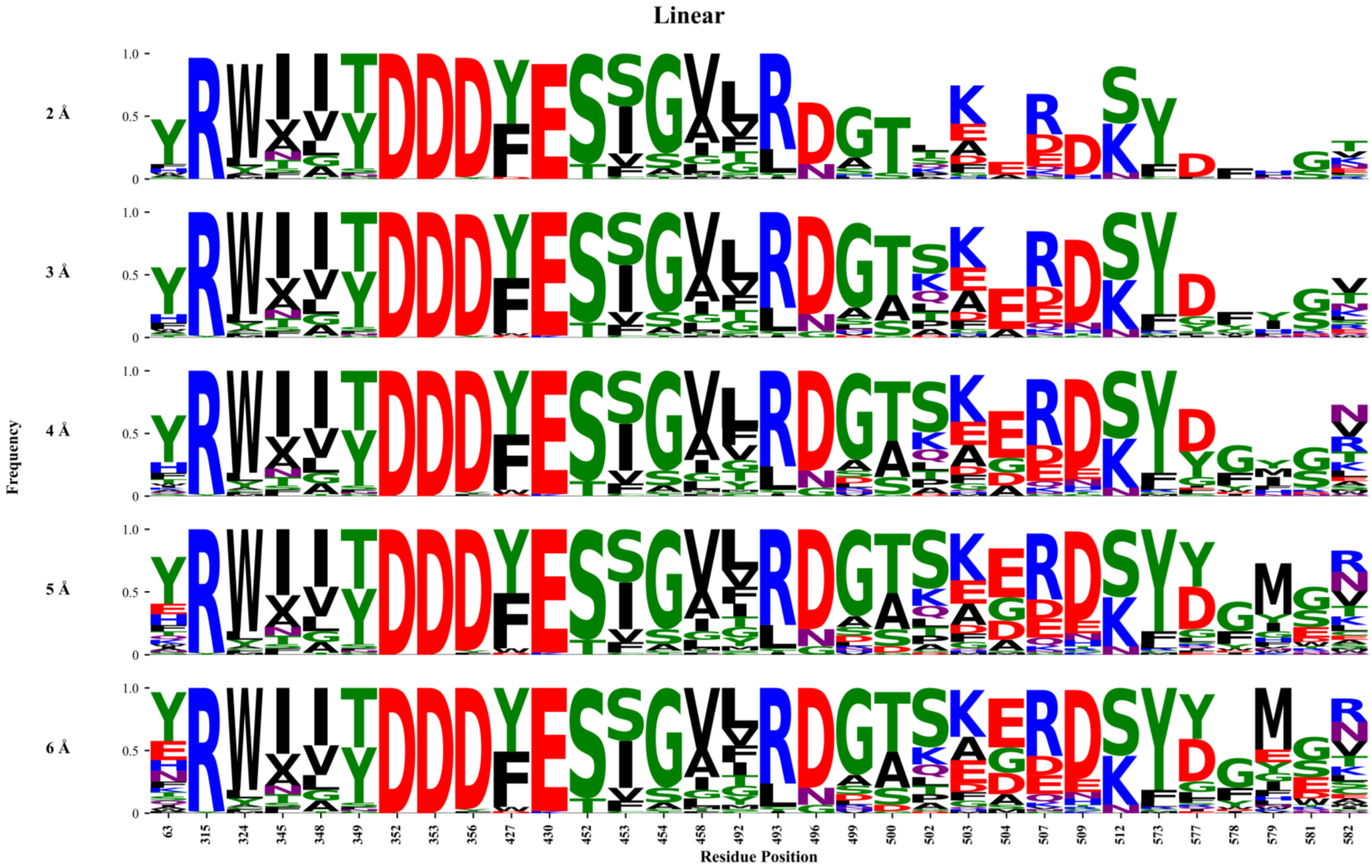

(continued on next page)

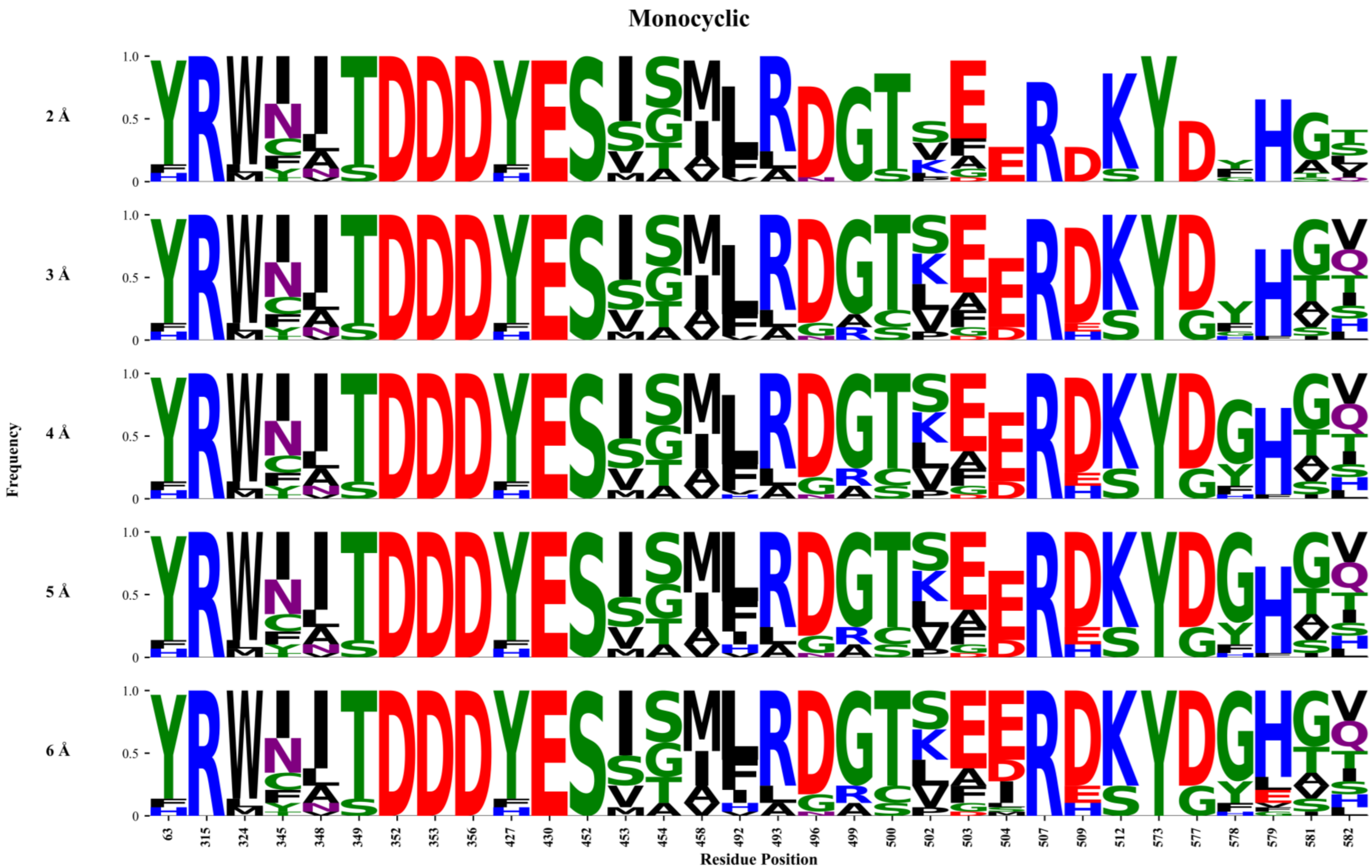

(continued on next page)

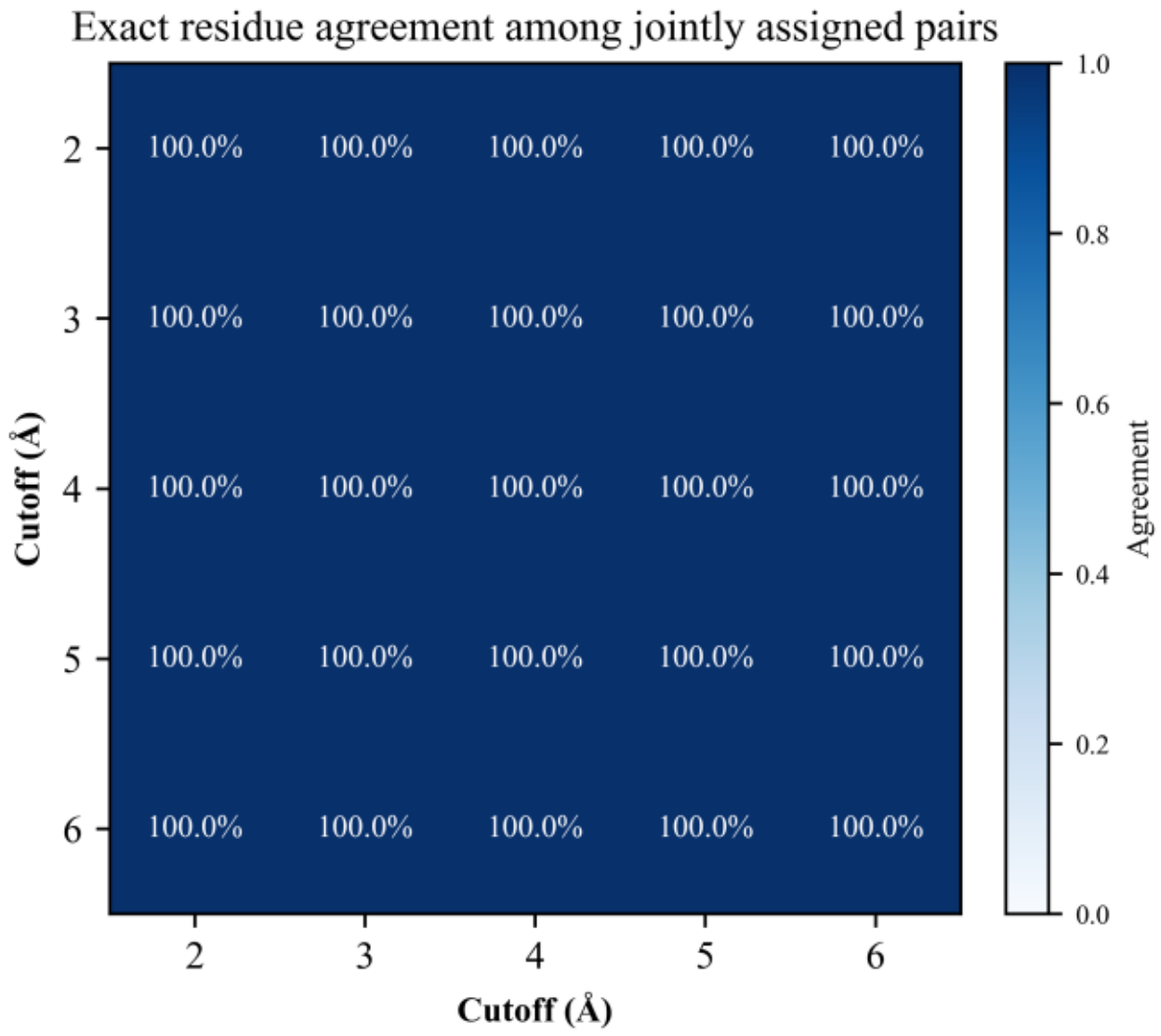

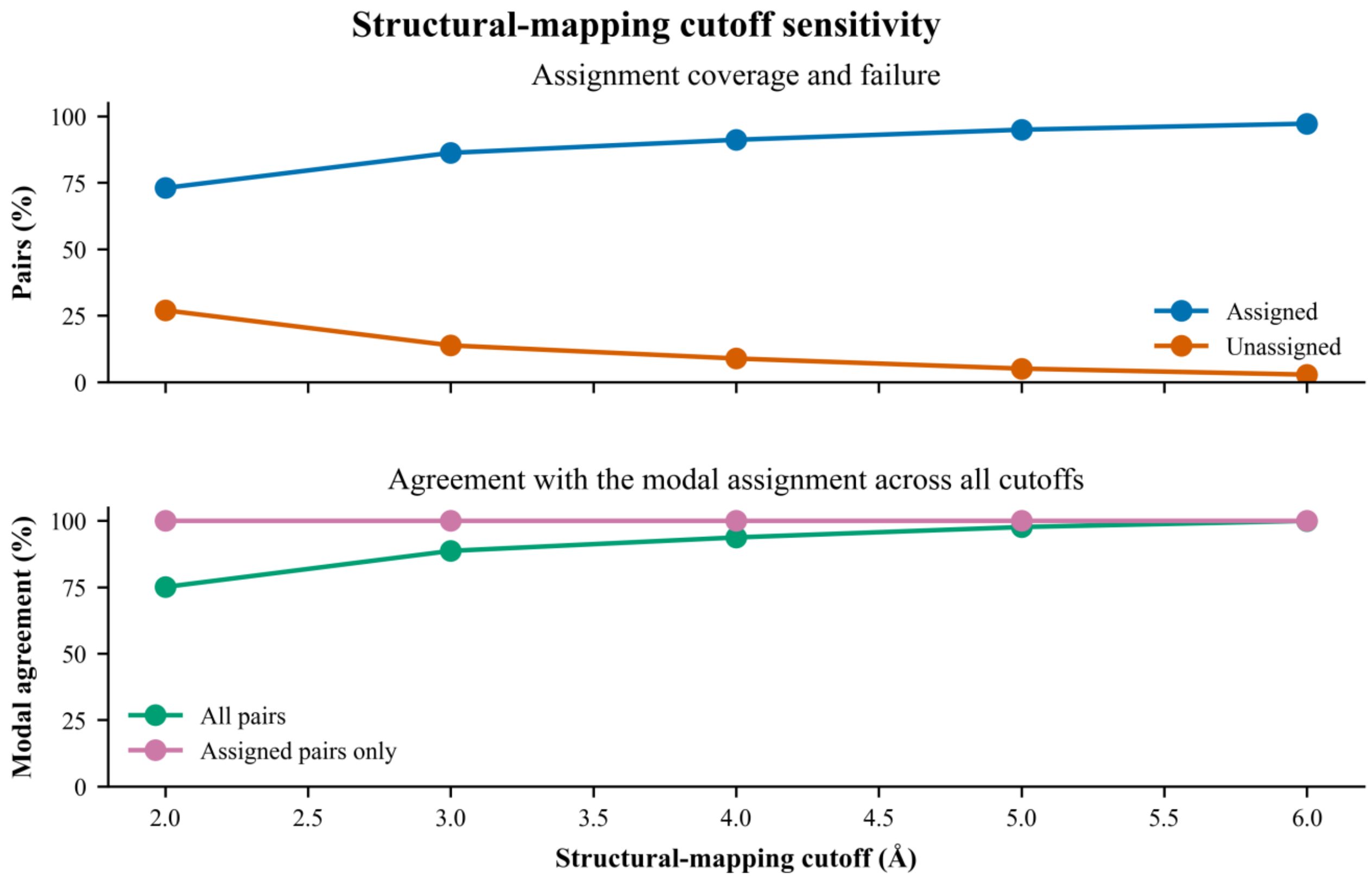

Figure S7: *Sensitivity of structural residue mapping to the distance cutoff. Sequence logos show residue frequencies at 32 selected reference positions for 59 Linear structures (A) and 29 Monocyclic structures (B), using cutoffs of 2, 3, 4, 5 and 6 Å. Letter height represents frequency across all structures, such that total stack height also indicates alignment occupancy. The dominant residue remained unchanged across all cutoffs at 27 of 32 Linear positions and 30 of 32 Monocyclic positions. Pairwise comparisons showed 100% residue agreement among the 2,033-2,644 protein-position pairs assigned at both cutoffs (C). Assignment coverage increased from 73.0% at 2 Å to 97.2% at 6 Å, while the failure rate decreased from 27.0% to 2.8% (D, upper). Agreement with the modal assignment remained 100% among assigned pairs, whereas agreement across all pairs increased with assignment coverage (D, lower). Cutoff selection therefore primarily affected whether a residue was assigned rather than its identity when assigned.*

Table S11*: Comparison of dominant active-site residue frequencies (from sequence logos) obtained using alternative reference structures. Structurally equivalent active-site residues were identified using structural alignment between the plant monoterpene synthase X-ray crystal structure 2ONG and the bacterial terpene synthase 5NX5. For each structurally matched position, dominant amino acid frequencies were independently calculated for linear (top) and cyclising (bottom) enzyme groups using identical protein datasets, differing only in the choice of reference structure used for active-site residue definition. Dominant residues and accompanying frequency distributions are shown for analyses performed using either the plant-derived (2ONG) or bacterial (5NX5) structural reference. Despite substantial size differences between the reference structures (329 residues in the bacterial 5NX5 and 599 in the plant 2ONG), dominant residue identity is retained at 82% of structurally equivalent positions, demonstrating that inferred conservation patterns are largely independent of reference structure selection*

2ONG numbering	5NX5 numbering	2ONG dominant	2ONG secondary	5NX5 dominant	5NX5 secondary	Identity
345	72	I (0.53)	—	I (0.50)	V(0.16)	Yes
348	75	I (0.46)	V(0.24)	I (0.47)	V(0.24)	Yes
349	76	T (0.47)	Y(0.44)	T (0.48)	Y(0.45)	Yes
352	79	D (1.00)	—	D (1.00)	—	Yes
353	80	D (1.00)	—	D (1.00)	—	Yes
452	175	S (0.88)	—	S (0.90)	—	Yes
453	176	S (0.42)	I(0.37)	S (0.43)	I(0.36)	Yes
454	177	G (0.80)	—	G (0.81)	—	Yes
458	181	V (0.49)	A(0.22)	V (0.50)	A(0.22)	Yes
492	214	L (0.44)	—	L (0.50)	—	Yes
496	218	D (0.80)	—	D (0.79)	—	Yes
500	222	T (0.47)	A(0.29)	T (0.48)	A,S	Yes
502	225	S (0.41)	K(0.15)	E (0.31)	K,A	No
504	226	E (0.53)	D(0.15)	E (0.33)	G(0.22)	Yes
512	233	S (0.54)	K(0.39)	S (0.55)	K(0.40)	Yes
573	295	Y (0.81)	—	L (0.38)	I(0.28)	No

577	299	D (0.34)	Y(0.20)	M (0.26)	A(0.19)	No

**2ONG numbering**	**5NX5 numbering**	**2ONG dominant**	**2ONG secondary**	**5NX5 dominant**	**5NX5 secondary**	**Identity**
345	72	I (0.39)	N(0.25)	I (0.38)	N(0.28)	Yes
348	75	I (0.61)	—	I (0.62)	—	Yes
349	76	T (0.86)	—	T (0.86)	—	Yes
352	79	D (1.00)	—	D (1.00)	—	Yes
353	80	D (1.00)	—	D (1.00)	—	Yes
452	175	S (1.00)	—	S (1.00)	—	Yes
453	176	I (0.50)	S,V	I (0.52)	S,V	Yes
454	177	S (0.39)	G,T	S (0.41)	G,T	Yes
458	181	M (0.46)	I(0.29)	M (0.48)	I(0.28)	Yes
492	214	L (0.61)	F(0.21)	L (0.69)	F(0.17)	Yes
496	218	D (0.82)	—	D (0.83)	—	Yes
500	222	T (0.75)	—	T (0.76)	—	Yes
502	225	S (0.32)	K,L	E (0.69)	—	No
504	226	E (0.86)	—	E (0.55)	D(0.17)	Yes
512	233	K (0.75)	S	K (0.76)	S	Yes
573	295	Y (1.00)	—	L (0.62)	—	No
577	299	D (0.75)	G(0.25)	M (0.41)	V(0.24)	No

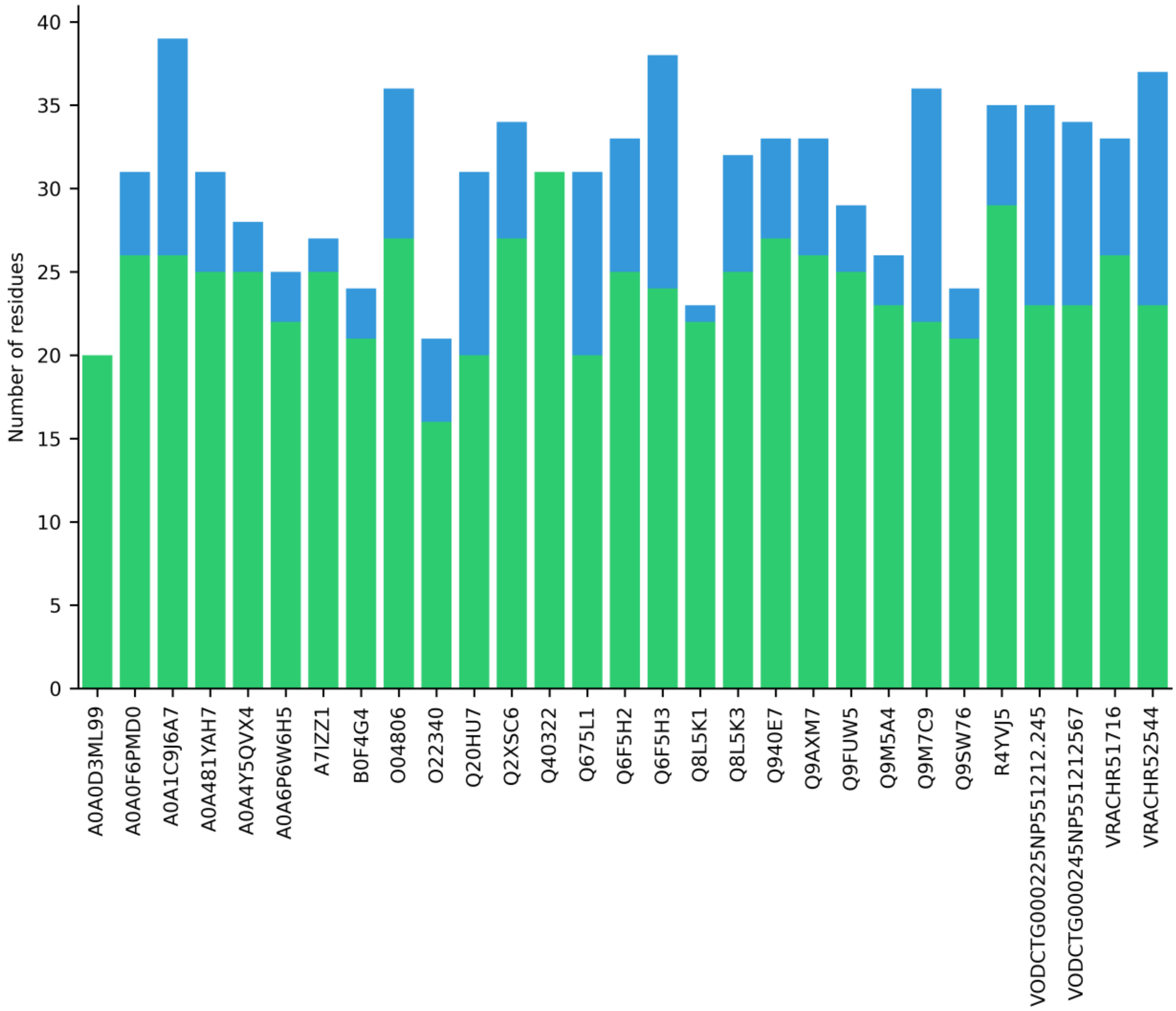

(continued on next page)

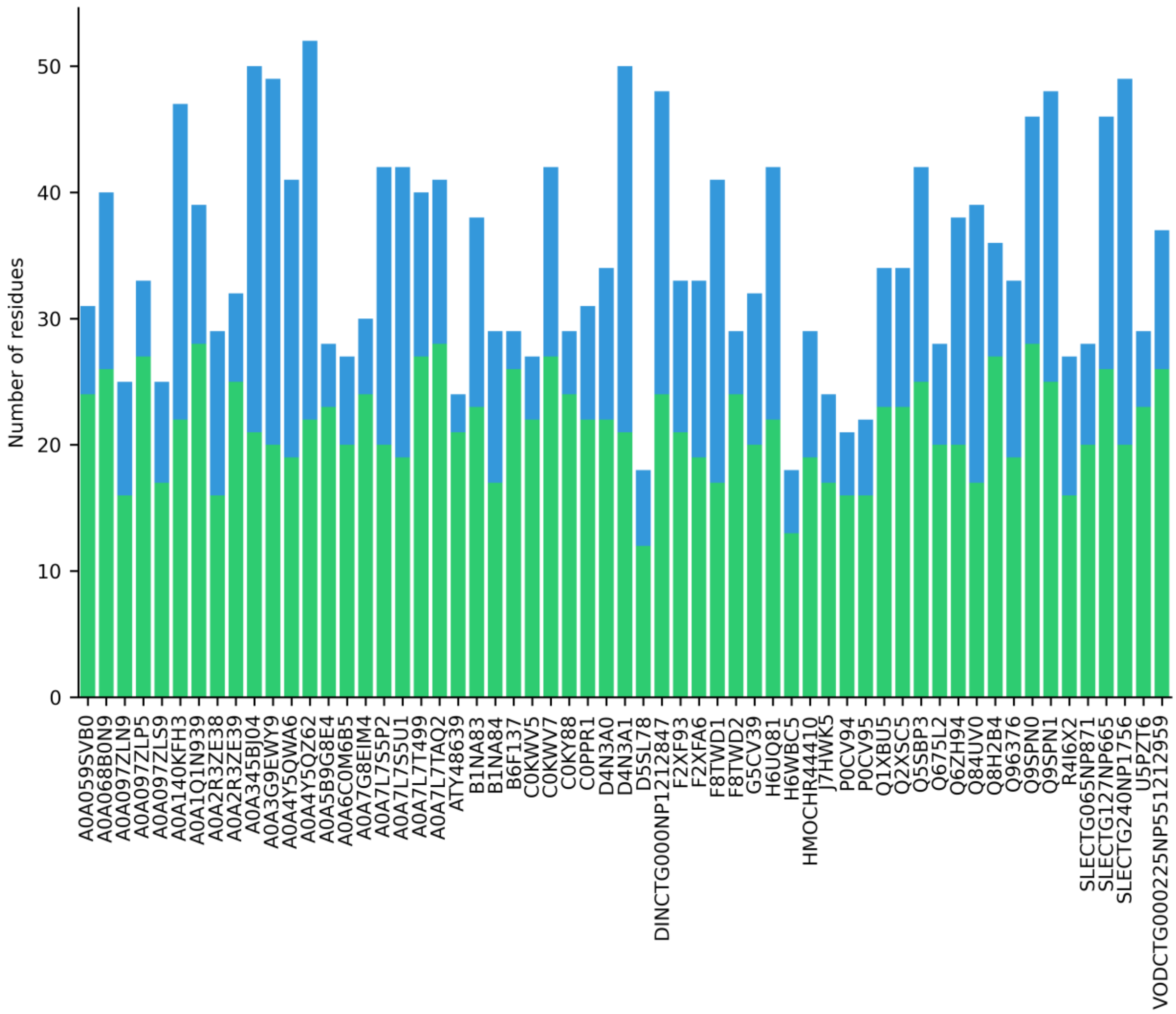

Figure S8: *Comparison of residues identified by Fpocket for each individual structure with those determined by structural alignment, performed as in Figure 4, using the cyclic limonene synthase reference structure (PDB ID: 2ONG). The graphs show cyclic (top) and linear (bottom) monoterpene synthase groups compared against the cyclic 2ONG reference. Green indicates residues identified by Fpocket that are also present among residues defined by the structural alignment, whereas blue indicates residues identified solely by Fpocket and not present in the alignment-derived active site definition.*

Machine Learning workflow and classification

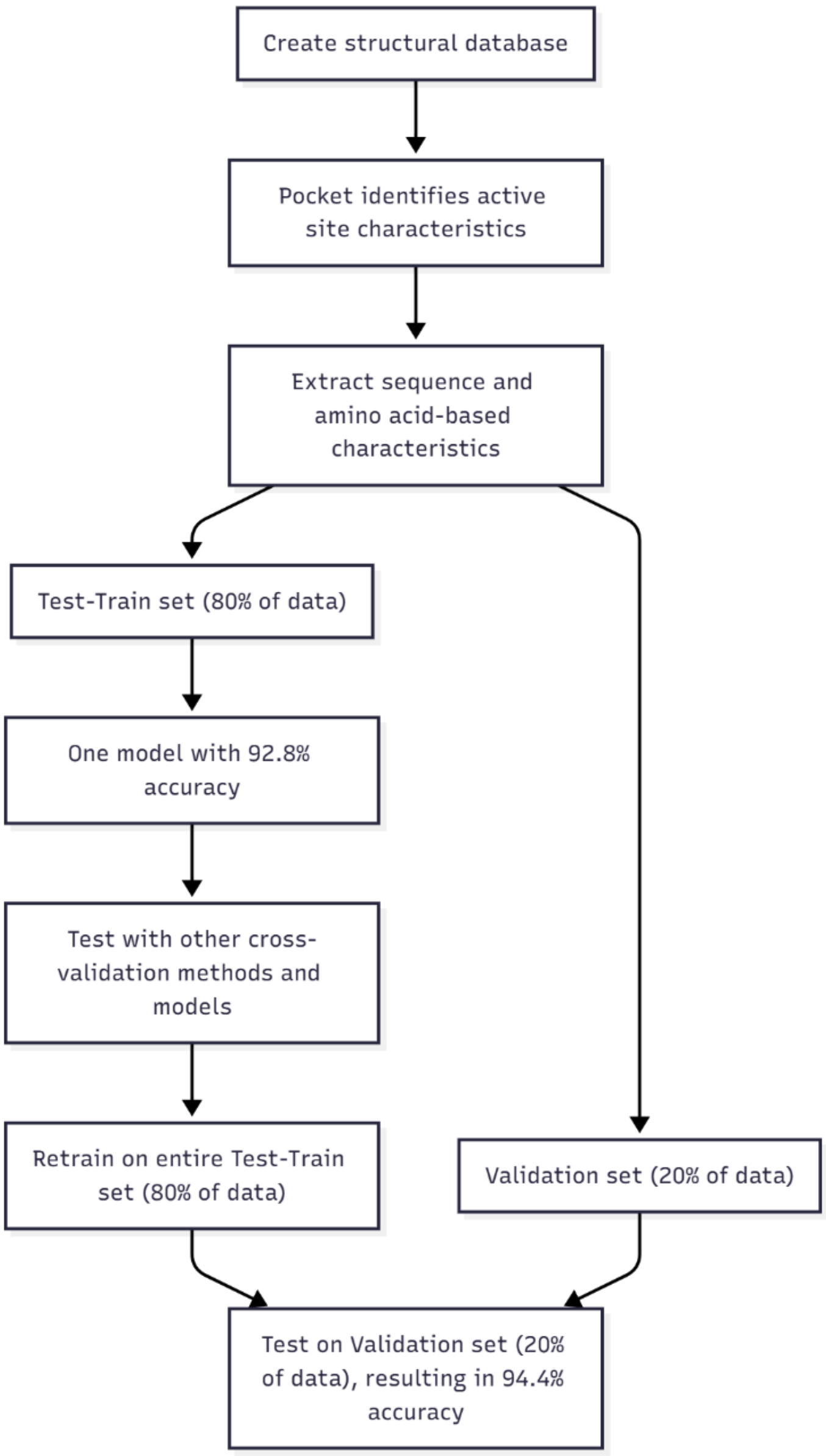

Figure S9: *Workflow illustrating machine learning model development and validation. Structural models were first generated, and Fpocket was used to identify active site characteristics. These were combined with sequence- and amino acid-based features to produce the full feature set. The dataset was then divided into a test-train set (80% of data) and a validation set (20% of data). An initial XGBoost model trained on the test-train set achieved 92.8% accuracy. This model was further evaluated using additional cross-validation methods and classifiers, followed by retraining on the full test-train data. The final model, tested on the hold-out validation set, achieved 94.4% accuracy.*

Extreme gradient boosting (XGBoost)[58] is a decision tree-based supervised ML algorithm that has been shown to build accurate classification models and can perform well with relatively small datasets.[59–61] Additionally, as these models are interpretable, we can understand and learn from which features influence the ability of the model to distinguish between the groups. Bayesian optimization (BO) has also been shown to be useful for feature selection for high-dimensional data in making molecular biology XGBoost models.[62,63] Fpocket can generate up to 46 features describing the active site. Using all of these features would result in ML models with too large of a search space, so non-amino acid features were grouped based on redundancy between the features. Prior to any feature-space exploration, the dataset was reduced to a ~80% test/train (69 members; 47 linear and 22 cyclic) and a ~20% validation set (18 members, 12 linear & 6 cyclic). Due to the modest size of the dataset, leave-one-out (LOO) cross-validation was also used to increase performance estimation.[64,65] This

method of cross-validation, each round, leaves an observation out of the training dataset and only tests on that one observation and repeats this for all of the observations within the dataset. It is more computationally expensive than other cross-validation methods, however it has been shown to minimize the chance of overfitting[66] and has been used successfully in ML models for protein/enzymology before.[67–69]

By grouping features into 'feature groups', where redundant, or biochemically similar features were grouped, resulting in 27 feature groups (where only one from the group can be tested each round), including each individual amino acid. This means, on top of the amino acids, there are seven feature groups, where only one subgroup (subgroups can contain paired features, e.g. 'charged' and 'uncharged', or individual features, e.g. 'charge score') can be selected from each group (**Table S12**). Bayesian optimization was carried out to find the best combination of each of these feature groups. This grouping allowed reduction of the number of features whilst reducing the testing space needed. BO-LOO-XGBoost resulted in a model with 92.8% ('feature set 1') accuracy with the lowest accuracy model found was 71.0%, which is particularly low, given it is a binary classification model. This feature set, showed low correlation between the features (**Figure S12**).

Table S12: *A Definition of 27 feature groups used in model selection. Individual amino acids were each assigned to their own group (Groups 1–20), while additional physicochemical and structural descriptors were consolidated into seven broader groups (Groups 21–27). Within each group, only one feature or subgroup could be selected during model optimization, with subgroups including either paired descriptors (e.g., charged/uncharged) or individual scores (e.g., charge score).*

Group	*Feature Group / Subgroups*
1	Exclude, Glycine – G
2	Exclude, Alanine – A
3	Exclude, Leucine – L
4	Exclude, Methionine – M
5	Exclude, Phenylalanine – F
6	Exclude, Tryptophan – W
7	Exclude, Lysine – K
8	Exclude, Glutamine – Q
9	Exclude, Glutamic Acid – E
10	Exclude, Serine – S
11	Exclude, Proline – P
12	Exclude, Valine – V
13	Exclude, Isoleucine – I
14	Exclude, Cysteine – C
15	Exclude, Tyrosine – Y
16	Exclude, Histidine – H
17	Exclude, Arginine – R
18	Exclude, Asparagine – N
19	Exclude, Aspartic Acid – D
20	Exclude, Threonine – T
21	Exclude, Charged | Uncharged, Charge Score, Positive charge | Negative charge, Pos-neg charge ratio
22	Exclude, Polar | Non-polar, Polarity Score, Hydrophobicity Score, Local hydrophobic density Score, Number of apolar alpha sphere, Proportion of apolar alpha sphere, Hydrophobic, Mean alpha-sphere solvent acc
23	Exclude, Drug Score
24	Exclude, Small, Number of alpha spheres | Mean alpha-sphere radius, Pocket volume (Monte Carlo), Pocket volume (Convex hull)
25	Exclude, Amino Acid based volume Score
26	Exclude, Aromatic
27	Exclude, Number of residues

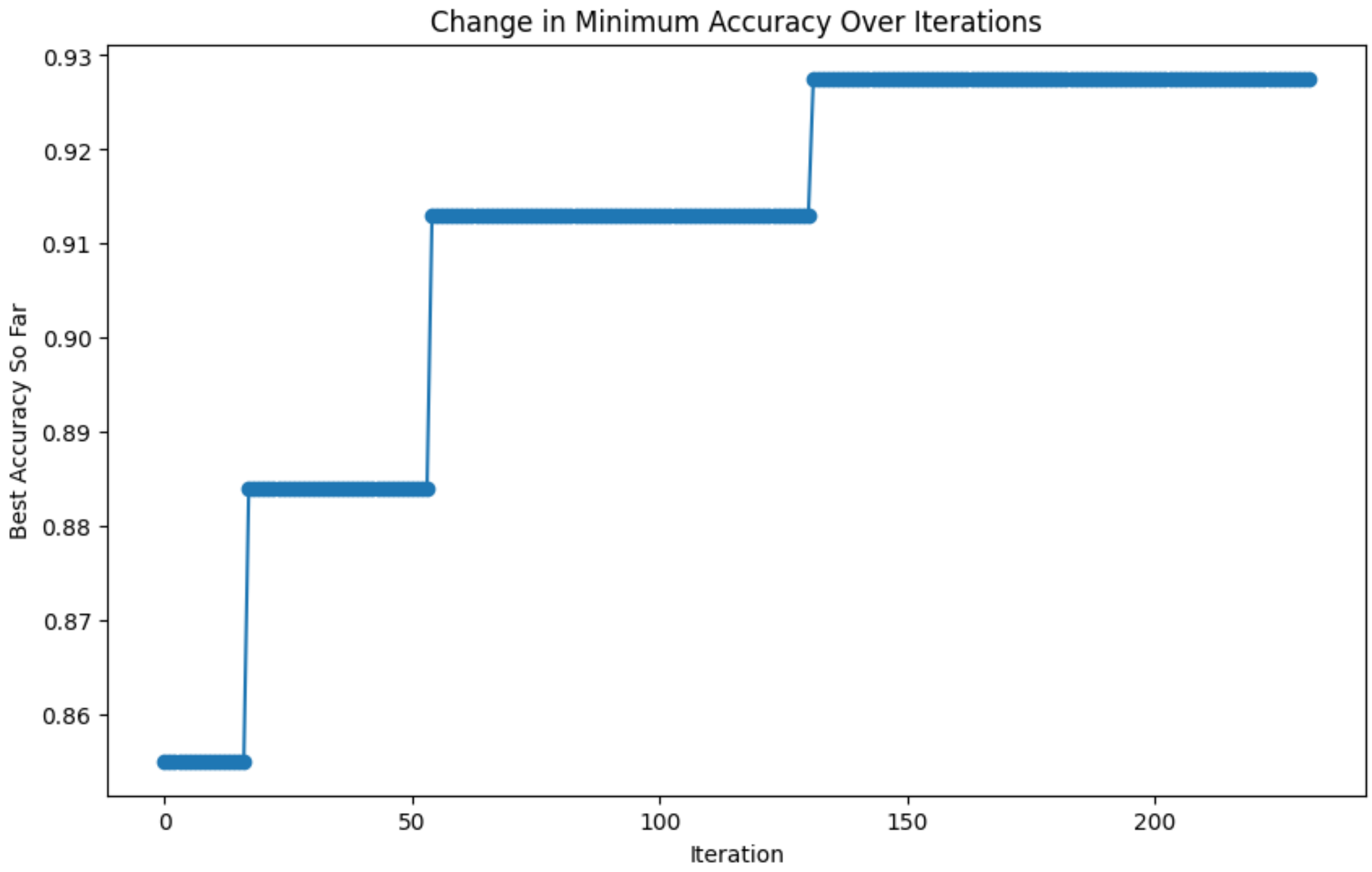

Figure S10: *Evolution of minimum accuracy in the leave-one-out cross-validation (LOO-CV) model over successive iterations of Bayesian optimization. Optimization was carried out on the 27 predefined feature groups (**Table S12**). While Bayesian optimization yielded only modest gains in maximum accuracy, it substantially reduced the number of features required to build the model, highlighting the value of subgroup-based feature selection.*

Table S13: *Variables included in 'feature set 1' (features output from the most accurate model discovered using Bayesian optimization). How the variables associated with pocket characteristics are generated can be found in the Fpocket source paper,[55] those measuring amino acid properties (AA) are from published literature.[56]*

**Feature Set 1 Variables**
Amino Acid based volume Score
Alanine – A (AA)
Methionine – M (AA)
Tryptophan – W (AA)
Isoleucine – I (AA)
Cysteine – C (AA)
Histidine – H (AA)
Asparagine – N (AA)
Positive charge (AA)
Negative charge (AA)
Polar (AA)
Non-polar (AA)

Of the 12 features within FS1 (**Figure 6**), several are known to influence cyclisation. Gain and mean absolute SHAP importance were calculated across the repeated stratified five-fold cross-validation of the full dataset. Directional SHAP values were obtained from the out-of-fold predictions, with positive values favouring cyclic predictions and negative values favouring linear predictions. Histidine showed the highest importance by both mean gain and mean absolute SHAP (first for both).

The presence of one or more histidine residues strongly favoured cyclic predictions, whereas their absence favoured linear predictions. This agrees with the reported role of histidine in promoting α-terpinyl cation formation.[70,71] Cysteine also showed high gain-based importance (third highest), but was lower by mean absolute SHAP (seventh). Two cysteine residues favoured cyclic predictions, while the absence of cysteine favoured linear predictions, although contributions from a single cysteine were smaller and mixed. Cysteine has previously been linked to differences in cyclisation type rather than the initial 1,6-ring closure[72,73] Amino acid-based volume score showed relatively high importance by both measures (sixth by mean gain and fourth by mean absolute SHAP). Scores at the upper end of the observed range generally favoured cyclic predictions, whereas many lower and intermediate scores favoured linear predictions, suggesting a nonlinear relationship. This supports previous findings that pocket geometry can influence cyclisation.[74,75] Polarity-related variables were among the most influential, although their importance differed between the two measures.

Non-polar residue count showed high importance by both mean gain and mean absolute SHAP (second for both). Higher non-polar residue counts favoured linear predictions, whereas lower counts favoured cyclic predictions. Polar residue count was lower by mean gain (seventh) but higher by mean absolute SHAP (third). Higher polar residue counts generally favoured cyclic predictions and lower counts favoured linear predictions, although this directional relationship was less distinct than for non-polar residue count. Polarity-related active-site variables have previously been shown to affect cyclisation, primarily by influencing the type or number of cyclisation events.[72,76] Charge-related descriptors showed lower gain-based importance, with positive and negative charge placing ninth and tenth, respectively. Their importance was higher by mean absolute SHAP, particularly for positive charge (fifth), while negative charge remained lower (eighth). Higher positive-charge values favoured cyclic predictions, whereas lower values favoured linear predictions. Negative charge showed a nonlinear relationship, with intermediate values, particularly seven negatively charged residues, favouring cyclic predictions, while lower and higher values generally favoured linear predictions. Charge-related active-site properties have previously been shown to affect product specificity.[77–79]

Of the individual amino acids, tryptophan showed relatively high mean gain across the complete-dataset repeated cross-validation (fourth) but was lower by mean absolute SHAP (tenth). Two tryptophan residues favoured linear predictions, whereas zero or one made only small contributions towards cyclic predictions. Previous work has shown that tryptophan can affect whether a product is linear or cyclic.[71] Isoleucine showed moderate importance by both mean gain and mean absolute SHAP (eighth and sixth, respectively). Zero or one isoleucine residue favoured cyclic predictions, whereas two or more favoured linear predictions. This agrees with previous work showing that introducing an isoleucine while removing a methionine through the M458I substitution in a limonene synthase resulted in approximately 60% more linear products. A T344I substitution in a fenchol synthase produced a similar but smaller change of approximately 10%.[80] In contrast, methionine showed the lowest importance by both mean gain and mean absolute SHAP (twelfth for both). Its SHAP values were close to zero, with no consistent directional relationship between methionine count and model prediction.

Asparagine also showed low importance by both measures (eleventh for both mean gain and mean absolute SHAP), with small SHAP contributions and no consistent relationship between residue count and model prediction. Despite its limited importance in the model, asparagine is essential for the metal binding required for terpene synthase activity and can also influence water activation[81], or the closed enzyme conformation in sesquiterpene synthases. However, asparagine ranked eleventh by mean absolute SHAP and made only small contributions, with no consistent relationship between residue count and model prediction. Alanine showed relatively high gain-based importance (fifth), but was lower by mean absolute SHAP (ninth). Zero or one alanine residue generally favoured linear predictions, whereas two or more favoured cyclic predictions. Previous studies have shown that

introducing alanine into the active sites of cyclic monoterpene synthases can reduce both overall activity and cyclisation capacity.[71,82,79,83] The importance and prevalence of these features in the final models may also reflect the hydroxylation of the end products used for model training. Future experimental work could investigate how these features contribute to mTS catalysis.

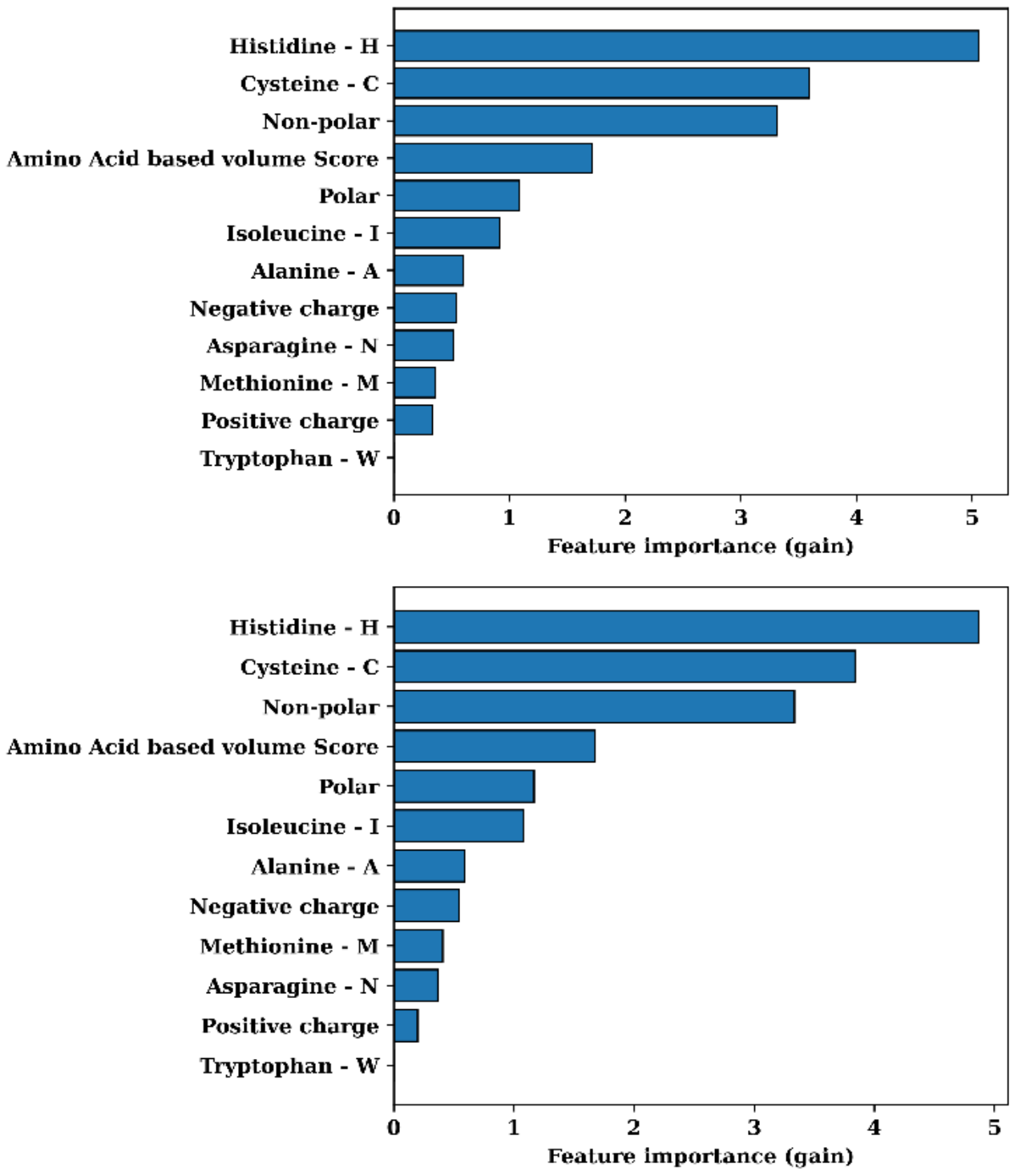

Figure S11: *Relative feature importance based on average gain from the XGBoost model. Results are shown separately for the LOO-XGBoost (top) and retraining on full test-train on validation (bottom). Very little differ from the relative feature importance, with only differences being seen in those with lowest relative importance.*

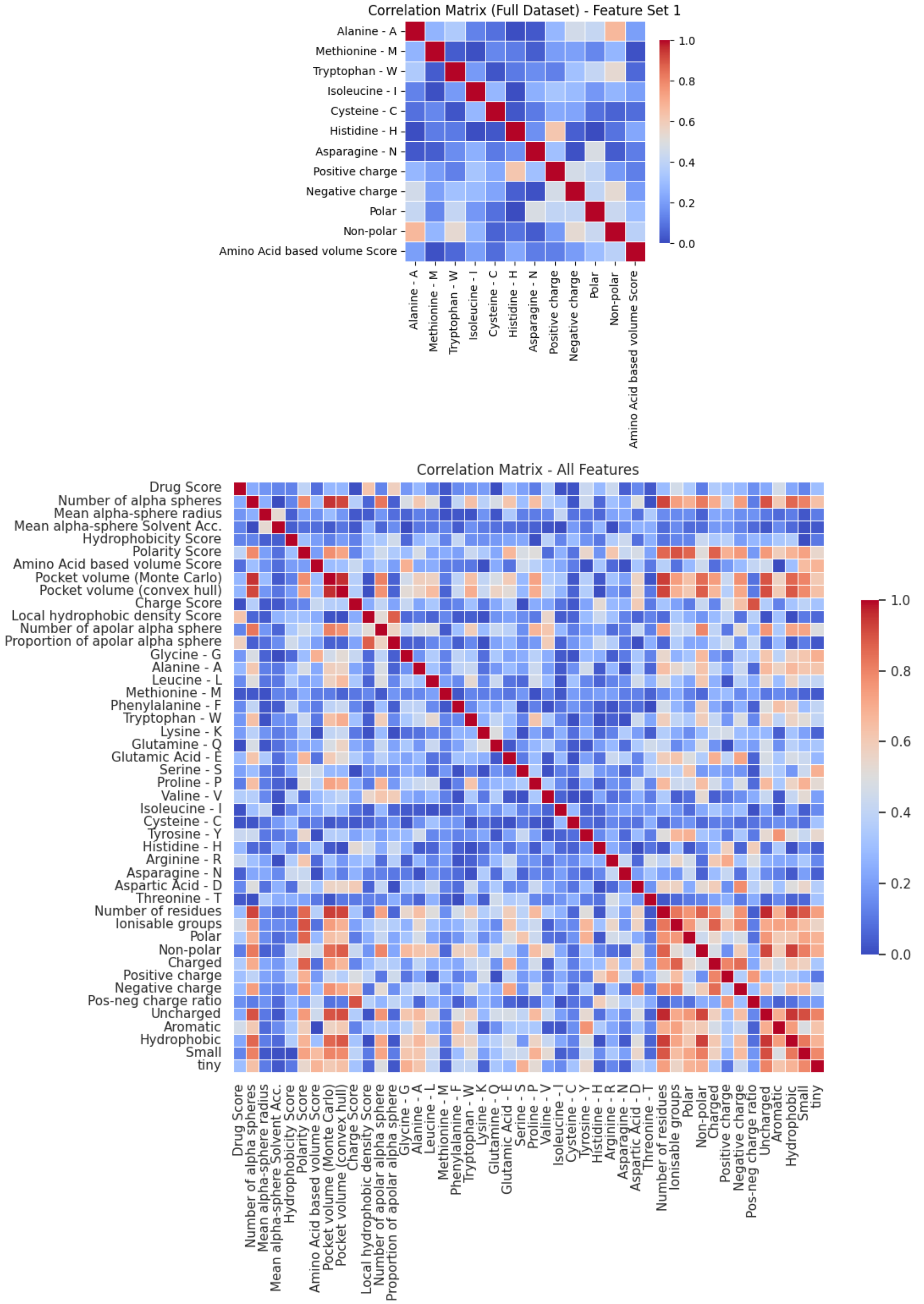

Figure S12: *Correlation matrix of feature set used in model construction, feature set 1 (FS1, top), all features (bottom). Within feature set 1, most features show low pairwise correlation, contrasting the full feature set, where many have high correlation.*

Table S14: *Outliers and prediction confidence for the final model (LOO-XGBoost, feature set 1; accuracy = 92.0%). Seven enzymes were misclassified - five linear (0) and two cyclic (1). Although more linear enzymes*

were misclassified in total, cyclic enzymes showed proportionally higher misclassification relative to their smaller class size.

Protein	True class	Predicted class	Confidence
A0A097ZLP5	Linear	Cyclic	98.90%
Q9M7C9	Cyclic	Linear	97.90%
Q2XSC6	Cyclic	Linear	97.50%
Q1XBU5	Linear	Cyclic	94.20%
A0A6C0M6B5	Linear	Cyclic	93.80%
hmoChr44410	Linear	Cyclic	74.10%
D5SL78	Linear	Cyclic	54.70%

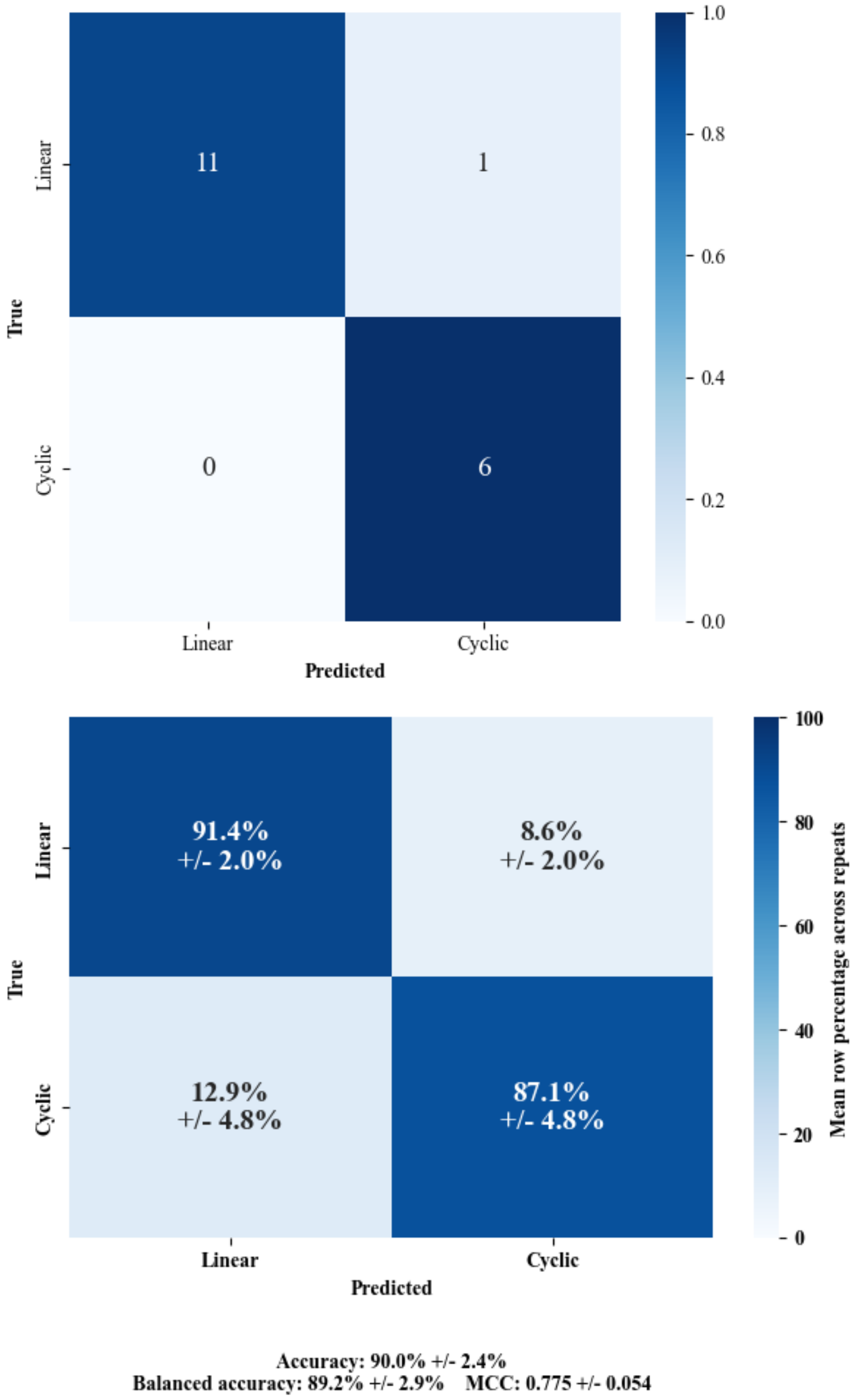

Figure S13: Confusion matrices for the FS1 XGBoost model. (Top) Independent holdout evaluation following training on the test/train dataset (69 enzymes) and evaluation on the holdout set (18 enzymes). Accuracy was 94.4%, balanced accuracy was 95.8%, and MCC was 0.886. For the linear class, precision was 100%, recall was 91.7%, specificity was 100%, and F1 score was 95.7%. For the cyclic class, precision was 85.7%, recall was 100%, specificity was 91.7%, and F1 score was 92.3%. (Bottom) Repeated stratified five-fold cross-validation across the complete dataset (87 enzymes; 10 repeats). Each cell shows the mean row percentage and SD across repeats. Mean accuracy was 90.0% (SD 2.4%), balanced accuracy was 89.2% (SD 2.9%), and MCC was 0.775 (SD 0.054). For the linear class, mean precision was 93.8% (SD 2.2%), recall was 91.4% (SD 2.0%), specificity was 87.1% (SD 4.8%), and F1 score was 92.5% (SD 1.7%). For the cyclic class, mean precision was 82.8% (SD 3.7%), recall was 87.1% (SD 4.8%), specificity was 91.4% (SD 2.0%), and F1 score was 84.8% (SD 3.7%).

Table S15: Classification performance comparison of machine learning models trained on reduced (FS1) versus full feature sets. Eight different algorithms were evaluated, including ensemble methods (XGBoost, Random Forest), support vector machines (SVM), partial least squares regression with logistic regression (PLS + LogReg), and various logistic regression approaches (L2, L1, ElasticNet, and Bayesian), using repeated stratified 5-fold cross-validation (10 repeats). Comparison across feature sets demonstrates the trade-offs between model complexity and performance, with the reduced FS1 feature set generally providing comparable or superior discriminatory power between linear and cyclic monoterpene synthases while enhancing interpretability across different approaches.

**Data-set**	**Model**	**Overall Accuracy (%)**	**Range (%)**	**Accuracy**					
				Balanced Mean (%)	Balanced Std Dev. (%)	Cyclic Mean (%)	Cyclic Std Dev. (%)	Linear Mean (%)	Linear Std Dev. (%)
**FS1**	XGBoost	83.50	57.1-100	80.50	10.90	71.50	21.60	89.40	8.60
**FS1**	Random Forest	88.50	69.2-100.0	87.20	9.40	83.20	17.50	91.20	9.50
**FS1**	SVM (linear)	81.00	53.8-100.0	79.70	10.20	75.90	19.70	83.60	12.60
**FS1**	PLS + LogReg	83.20	61.5-100.0	81.40	10.00	75.90	18.10	86.80	11.30
**FS1**	LogReg (L2)	82.40	69.2-100.0	80.20	9.20	74.20	19.40	86.20	10.10
**FS1**	LogReg (L1)	82.90	61.5-100.0	81.70	9.40	78.30	19.20	85.20	10.90
**FS1**	LogReg (ElasticNet)	82.80	71.4-100.0	81.30	9.50	77.10	20.10	85.60	9.30
**FS1**	Bayesian LogReg	82.50	69.2-100.0	80.20	9.50	73.80	19.40	86.60	9.80
**Full**	XGBoost	80.70	57.1-100.0	77.80	11.60	70.20	24.10	85.50	11.40
**Full**	Random Forest	86.20	71.4-100.0	83.40	9.50	75.60	20.50	91.30	9.40
**Full**	SVM (linear)	80.40	64.3-100.0	79.80	10.00	77.90	18.90	81.70	11.20
**Full**	PLS + LogReg	81.70	46.2-100.0	80.70	11.10	77.70	19.10	83.70	11.40
**Full**	LogReg (L2)	79.50	57.1-92.9	78.40	9.60	74.90	20.50	81.90	9.50
**Full**	LogReg (L1)	79.50	57.1-92.9	79.40	9.60	78.40	19.90	80.40	11.00
**Full**	LogReg (ElasticNet)	79.30	61.5-92.9	78.50	8.70	75.90	18.40	81.00	10.50
**Full**	Bayesian LogReg	79.50	57.1-92.9	78.60	9.20	75.70	19.50	81.50	10.10

Table S16: *Validation set performance comparison of various machine learning models. Eight different algorithms were evaluated, including ensemble methods (XGBoost, Random Forest), support vector machines (SVM), partial least squares regression with logistic regression (PLS + LogReg), and various logistic regression approaches (L2, L1, ElasticNet, and Bayesian), using both the reduced (FS1) feature set and the full feature set. All models were trained on the training dataset and evaluated on an independent validation set. This comparison demonstrates the performance of the final selected model (XGBoost with FS1 features) relative to alternative modelling approaches, confirming its suitability for discriminating between linear and cyclic monoterpene synthases on unseen data.*

Dataset	Model	Overall Accuracy	Balanced Accuracy	Cyclic Accuracy	Linear Accuracy
**FS1**	XGBoost	94.40%	95.80%	100.00%	91.70%
**FS1**	Random Forest	72.20%	62.50%	33.30%	91.70%
**FS1**	SVM (linear)	94.40%	91.70%	83.30%	100.00%
**FS1**	PLS + LogReg	83.30%	87.50%	100.00%	75.00%
**FS1**	LogReg (L2)	77.80%	83.30%	100.00%	66.70%
**FS1**	LogReg (L1)	72.20%	79.20%	100.00%	58.30%
**FS1**	LogReg (ElasticNet)	77.80%	83.30%	100.00%	66.70%
**FS1**	Bayesian LogReg	77.80%	83.30%	100.00%	66.70%
**FullFS**	XGBoost	94.40%	91.70%	83.30%	100.00%
**FullFS**	Random Forest	94.40%	91.70%	83.30%	100.00%
**FullFS**	SVM (linear)	100.00%	100.00%	100.00%	100.00%
**FullFS**	PLS + LogReg	88.90%	91.70%	100.00%	83.30%
**FullFS**	LogReg (L2)	88.90%	91.70%	100.00%	83.30%
**FullFS**	LogReg (L1)	83.30%	87.50%	100.00%	75.00%
**FullFS**	LogReg (ElasticNet)	88.90%	91.70%	100.00%	83.30%
**FullFS**	Bayesian LogReg	88.90%	91.70%	100.00%	83.30%

Table S17*: Additional observations from the dataset from the Pluskal group[84], including reported monoterpene products and their classification. A total of 9 enzymes are classified as linear (linalool-producing) and 5 as monocyclic (limonene-producing).*

UniProt ID	Monoterpene Compound	Monoterpene Category
A0A140AZ72	Linalool	Linear
A0A3S9GV71	Linalool	Linear
A0A7S5L324	Linalool	Linear
A0A7S5L3H2	Linalool	Linear
C0KWV3	Linalool	Linear
ErTC-1	Linalool	Linear
G1JUH4	Limonene	Monocyclic
G8H5M7	Limonene	Monocyclic
P0CV96	Linalool	Linear
Q29VN2	Linalool	Linear
Q75WN1	Limonene	Monocyclic
Q9FV72	Limonene	Monocyclic
R9W377	Limonene	Monocyclic
TmTC-1	Linalool	Linear

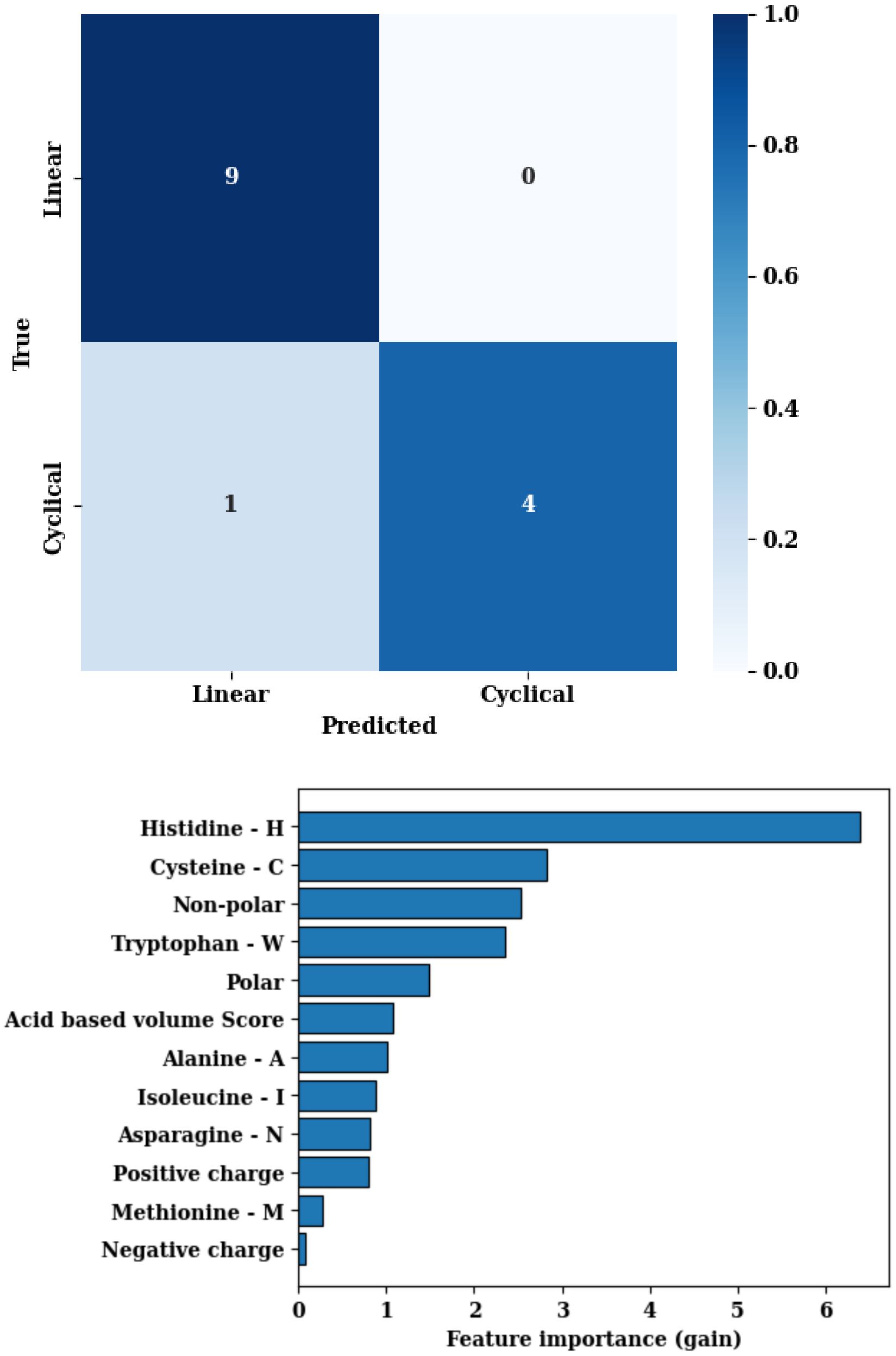

Figure S14: *Performance of the XGBoost model trained on the full curated dataset (87 observations), using the final feature set, and evaluated on the independent Pluskal dataset (14 observations). (Top) Relative feature importance (gain) from the final trained model. (Bottom) Confusion matrix showing classification of linear and cyclic monoterpene synthases. All nine linear enzymes and four of five cyclic enzymes were correctly predicted, giving an overall accuracy of 92.9%, balanced accuracy of 90.0% and MCC of 0.849. For the linear class, precision was 90%, recall was 100%, specificity was 80% and F1 score was 94.7%. For the cyclic class, precision was 100%, recall was 80%, specificity was 100% and F1 score was 88.9%. One cyclic enzyme (G1JUH4) was misclassified as linear with a confidence of 88.2%.*

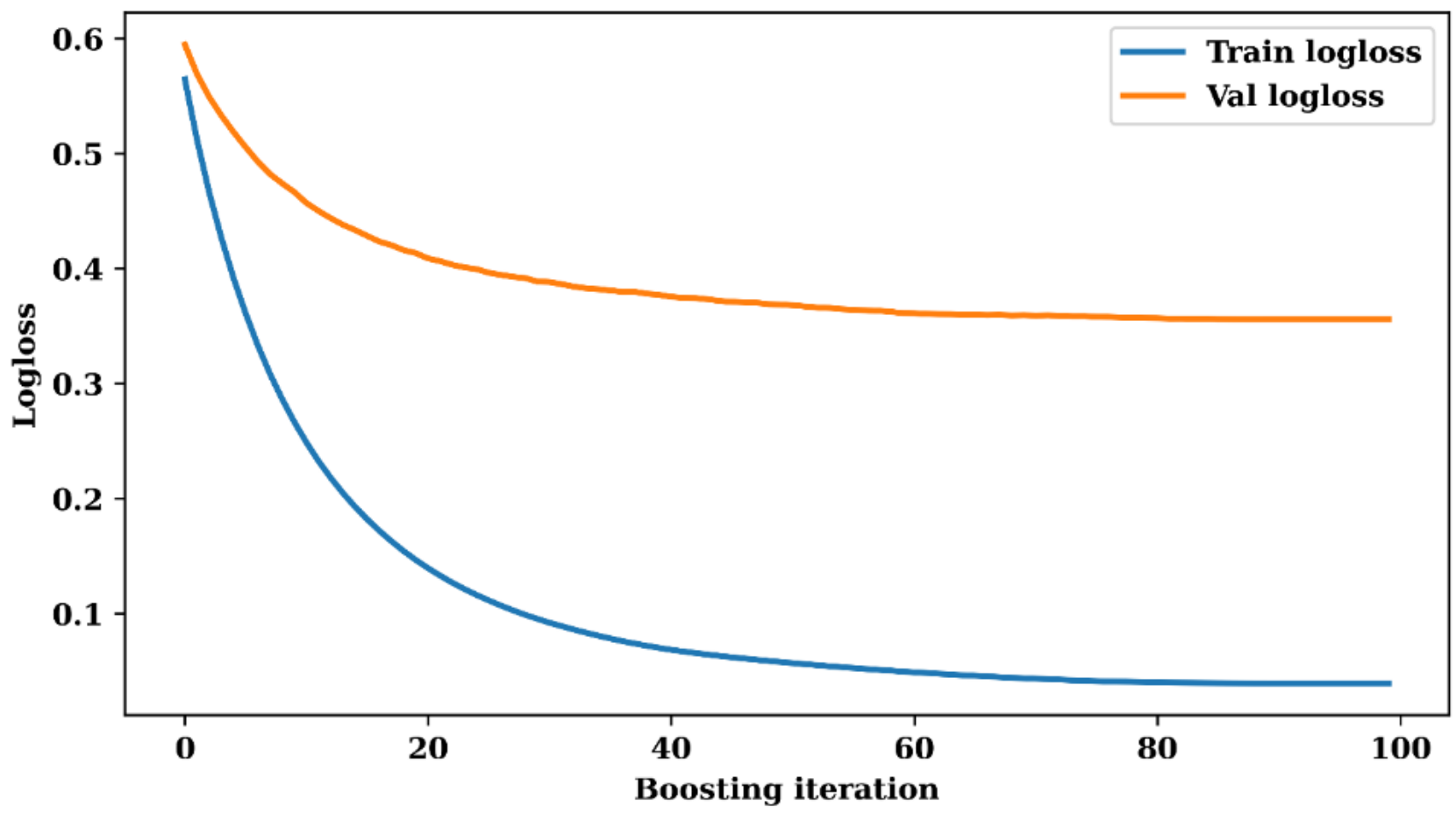

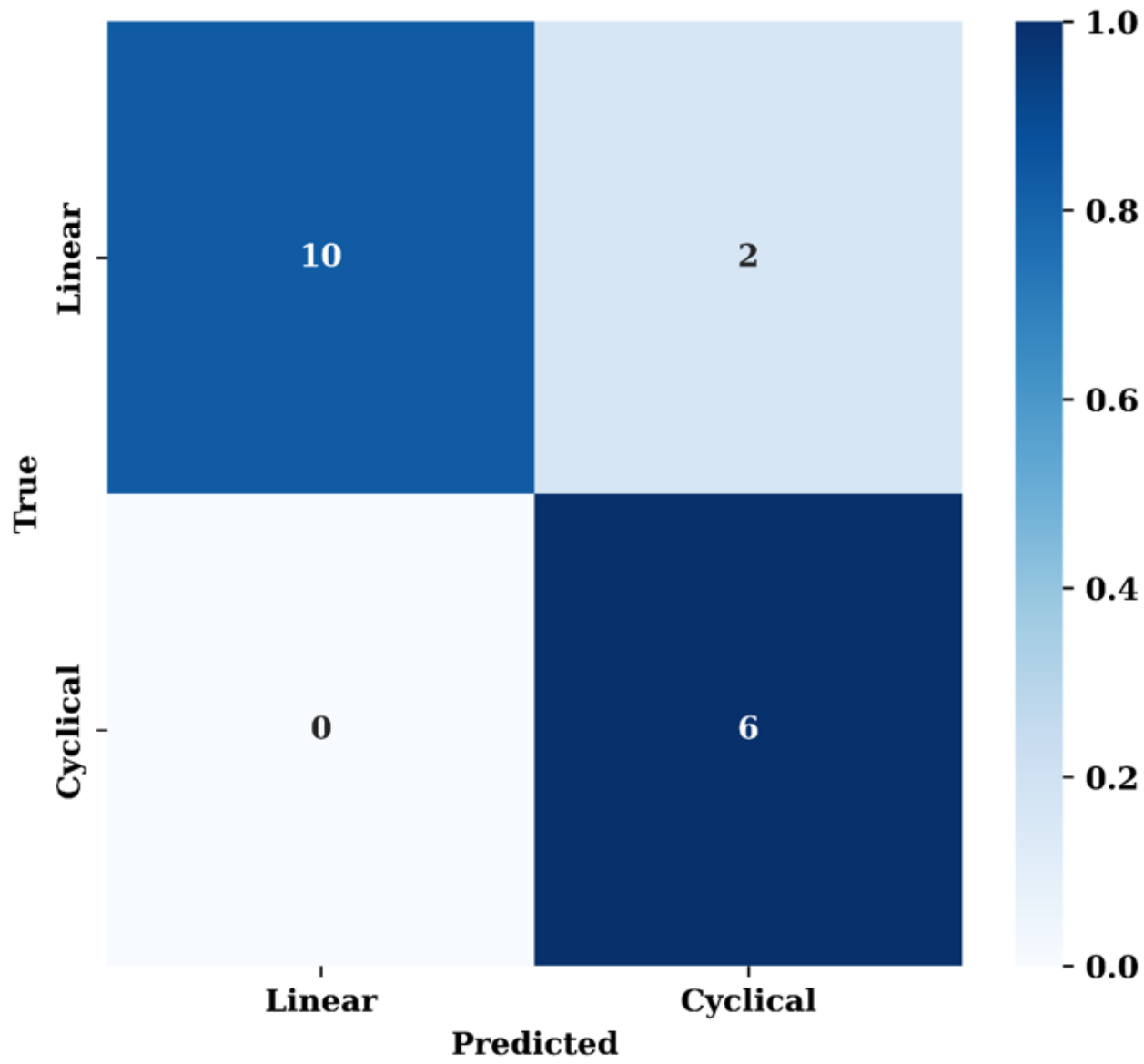

Figure S15: *(Top) Log loss curves for the XGBoost model trained on ESM-2*[85] *protein embeddings using the test-train set only, evaluated with repeated stratified k-fold cross-validation (k = 5, repeats = 10, total runs = 50). Training log loss (blue) converged rapidly to 0.04, while validation log loss (orange) stabilized at 0.36 by iteration 88, indicating the model's convergence without significant overfitting. The model achieved a mean accuracy of 86.6% ± 1 across all folds. The confusion matrix summed over all runs showed strong performance on the negative class (445 true negatives, 25 false positives) and moderate performance on the positive class (153 true positives, 67 false negatives), with an overall sensitivity of 69.5% and specificity of 94.7%. (Bottom) Confusion matrix for a holdout evaluation where the model was trained once on the entire test-train set (69 samples) and then tested once on the holdout validation set (18 samples). The matrix shows 88.9% overall accuracy on the test set, with two linear sequences misclassified as cyclic (ATY48639, confidence 98.4%; D5SL78, confidence 67.5%), while all cyclic sequences were correctly classified.*

Table S18*:* Performance comparison of *BLAST,*[86] *phmmer,*[87] *and Foldseek*[88] under random 5-fold cross-validation, as well as these models with XGBoost under random 5-fold cross-validation and homology-aware leave-one-cluster-out (LOCO) splits. Metrics include overall accuracy, balanced accuracy, class-specific precision, recall, F1-score, and Matthews correlation coefficient (MCC).

Method	Split	n	Overall Acc. (%)	Balanced Acc. (%)	Precision (L) (%)	Recall (L) (%)	F1 (L) (%)	Precision (C) (%)	Recall (C) (%)	F1 (C) (%)	MCC
BLAST	Random 5-fold	87	88.5	88.7	94.5	88.1	91.2	78.1	89.3	83.3	0.75
phmmer	Random 5-fold	86	88.4	88.6	94.4	87.9	91.1	78.1	89.3	83.3	0.749
Foldseek	Random 5-fold	87	89.7	88.6	93.1	91.5	92.3	82.8	85.7	84.2	0.765
BLAST	Seq. LOCO	87	66.7	62.3	75.9	74.6	75.2	48.3	50	49.1	0.244
phmmer	Seq. LOCO	86	61.6	54.9	70.5	74.1	72.3	40	35.7	37.7	0.102
Foldseek	Seq. LOCO	86	74.4	70.9	81	81	81	60.7	60.7	60.7	0.417
XGBoost	Seq. LOCO	87	87.4	86.9	92.9	88.1	90.4	77.4	85.7	81.4	0.72
Foldseek	Struct. LOCO	87	78.2	75.5	84.5	83.1	83.8	65.5	67.9	66.7	0.505
XGBoost	Struct. LOCO	87	93.1	93	96.5	93.2	94.8	86.7	92.9	89.7	0.846
XGBoost	Feat. LOCO	87	83.9	80.6	86.9	89.8	88.3	76.9	71.4	74.1	0.625

Metrics were calculated from pooled predictions across folds. LOCO splits were constructed to eliminate sequence-, structural-, or feature-based similarity between training and test sets. Sequence LOCO clusters were generated using MMseqs2 (min-seq-id = 0.5, coverage = 0.9); structural LOCO clusters using Foldseek (TM-score = 0.90, coverage = 0.95, e-value = 1e-5); feature-based LOCO clusters using K-means using full feature set. Baseline methods (BLAST, phmmer, Foldseek) use top-1 nearest-neighbor annotation transfer based on alignment scores.

Table S19: Experimentally characterized monoterpene synthases used to evaluate model transferability to enzymes producing products distinct from linalool and limonene. Enzymes are listed with their protein ID, species of origin, reported product, and the publication in which their activity was described. Products are classified as linear, monocyclic, or bicyclic monoterpenes for consistency with the classification framework used in this study.

Protein ID	Product (category)	Species	Paper Title
Q8GUE4	geraniol (linear)	*Cinnamomum tenuipile*	A geraniol-synthase gene from *Cinnamomum tenuipilum*[89]
PbTC1	geraniol (linear)	*Paramuricea biscaya*	Ancient plant-like terpene biosynthesis in corals[90]
Q84KL3	a-pinene (bicyclic)	*Pinus taeda*	cDNA isolation, functional expression, and characterization of (+)-α-pinene synthase and (−)-α-pinene synthase from *loblolly pine (Pinus taeda)*: Stereocontrol in pinene biosynthesis[91]
C3RSF5	a-pinene (bicyclic)	*Chamaecyparis formosensis*	Cloning and characterization of α-pinene synthase from *Chamaecyparis formosensis* Matsum[92]
Q94G53	b-pinene (bicyclic)	*Artemisia annua*	Cloning and Functional Characterization of a β-Pinene Synthase from *Artemisia annua* That Shows a Circadian Pattern of Expression[93]
PchChr33874	b-phellandrene (monocyclic)	*Parashorea chinensis*	Cyclization mechanism of monoterpenes catalyzed by monoterpene synthases in dipterocarpaceae[53]
PchChr33879	b-phellandrene (monocyclic)	*Parashorea chinensis*	Cyclization mechanism of monoterpenes catalyzed by monoterpene synthases in dipterocarpaceae[53]
dinctg061np1212 1087	b-pinene (bicyclic)	*Dipterocarpus intricatus*	Cyclization mechanism of monoterpenes catalyzed by monoterpene synthases in dipterocarpaceae[53]
sroctg002np1314	b-phellandrene (monocyclic)	*Shorea roxburghii*	Cyclization mechanism of monoterpenes catalyzed by monoterpene synthases in dipterocarpaceae[53]
dalctg267np498	Borneol (bicyclic)	*Dipterocarpus alatus*	Cyclization mechanism of monoterpenes catalyzed by monoterpene synthases in dipterocarpaceae[53]
dalctg267np499	a-terpineol (monocyclic)	*Dipterocarpus alatus*	Cyclization mechanism of monoterpenes catalyzed by monoterpene synthases in dipterocarpaceae[53]
dgrChr5258	Borneol (bicyclic)	*Dipterocarpus gracilis*	Cyclization mechanism of monoterpenes catalyzed by monoterpene synthases in dipterocarpaceae[53]
dzectg04np1007	Borneol (bicyclic)	*Dipterocarpus zeylanicus*	Cyclization mechanism of monoterpenes catalyzed by monoterpene synthases in dipterocarpaceae.[53]
dzectg04np1012	a-terpineol (monocyclic)	*Dipterocarpus zeylanicus*	Cyclization mechanism of monoterpenes catalyzed by monoterpene synthases in dipterocarpaceae.[53]
dinctg061np1212 1085	Borneol (bicyclic)	*Dipterocarpus intricatus*	Cyclization mechanism of monoterpenes catalyzed by monoterpene synthases in dipterocarpaceae.[53]
D7U6G8	E-b-ocimene (linear)	*Vitis vinifera, Gewürztraminer*	Differential expression pattern of an acidic 9/13-lipoxygenase in flower opening and senescence and in leaf response to phloem feeders in the tea plant[94]

A0A0M3Q1Q3	g-terpinene (monocyclic)	*Thymus vulgaris*	Expression, crystallization and structure elucidation of γ-terpinene synthase from *Thymus vulgaris*[95]
Q308N0	geraniol (linear)	*Perilla frutescens*	Geraniol synthases from perilla and their taxonomical significance[96]
Q93X23	b-myrcene (linear)	*Quercus ilex*	Isolation and functional analysis of a cDNA encoding a myrcene synthase from holm oak (Quercus ilex L.)[97]
G0Y7D1	E-b-ocimene (linear)	*Litsea cubeba*	Molecular cloning and characterization of monoterpene synthases from *Litsea cubeba (Lour.)* Persoon[98]
O24474	b-myrcene (linear)	*Abies grandis*	Monoterpene Synthases from Grand Fir (*Abies grandis*)[99]
B6SCF4	b-myrcene (linear)	*Humulus lupulus*	Terpene Biosynthesis in Glandular Trichomes of Hop[100]
A0A1V0QSH2	a-pinene (bicyclic)	*Cannabis sativa*	Terpene synthases from *Cannabis sativa*[101]
A0A1V0QSF4	a-terpinene (monocyclic)	*Cannabis sativa*	Terpene synthases from *Cannabis sativa*[101]
Q5SBP1	b-myrcene (linear)	*Ocimum basilicum*	The biochemical and molecular basis for the divergent patterns in the biosynthesis of terpenes and phenylpropenes in the peltate glands of three cultivars of basil[14]
Q6USK1	geraniol (linear)	*Ocimum basilicum*	The biochemical and molecular basis for the divergent patterns in the biosynthesis of terpenes and phenylpropenes in the peltate glands of three cultivars of basil[14]
R9QMW7	b-phellandrene (monocyclic)	*Pinus banksiana*	Transcriptome resources and functional characterization of monoterpene synthases for two host species of the mountain pine beetle, lodgepole pine (*Pinus contorta*) and jack pine (*Pinus banksiana*)[102]

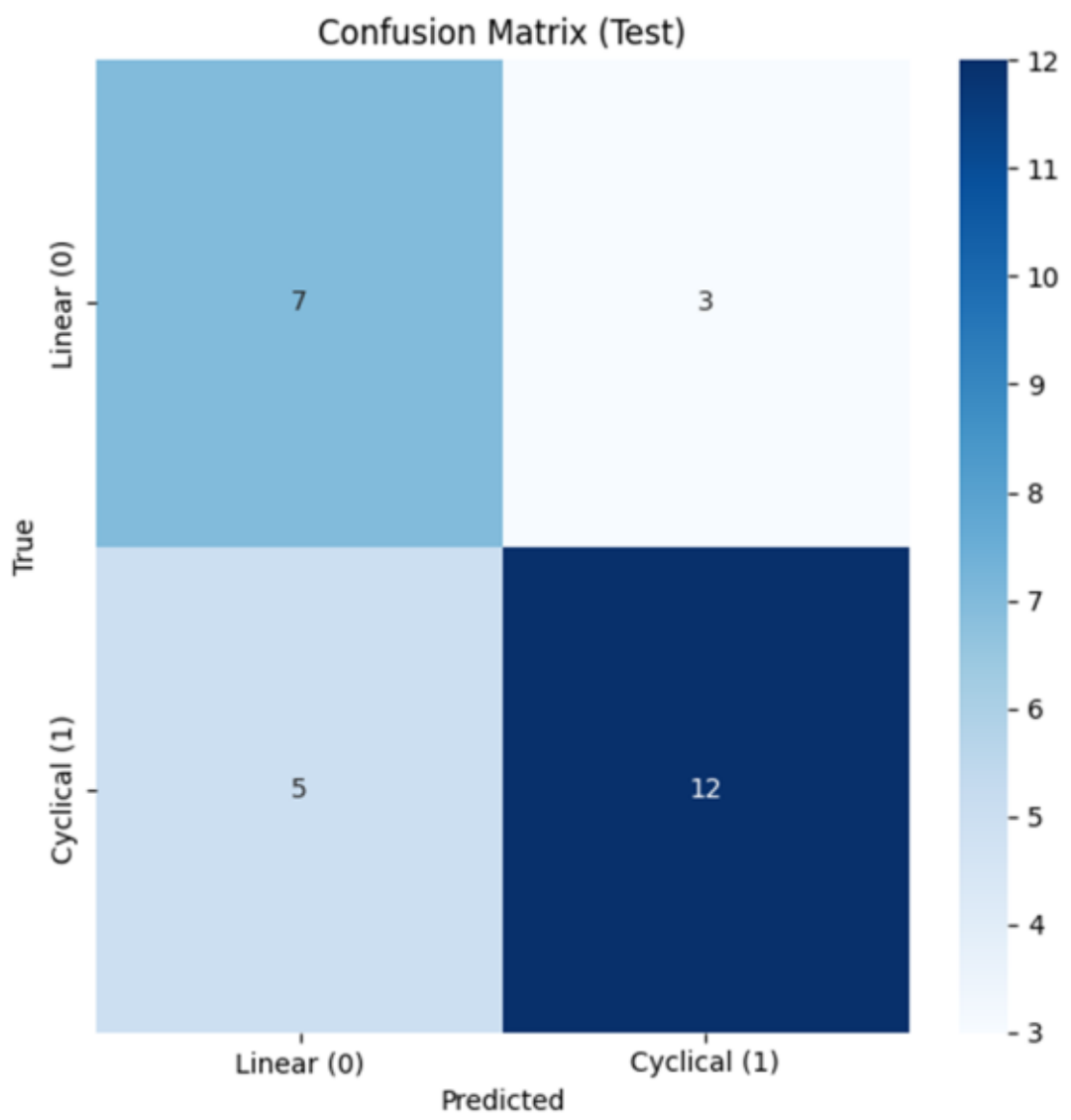

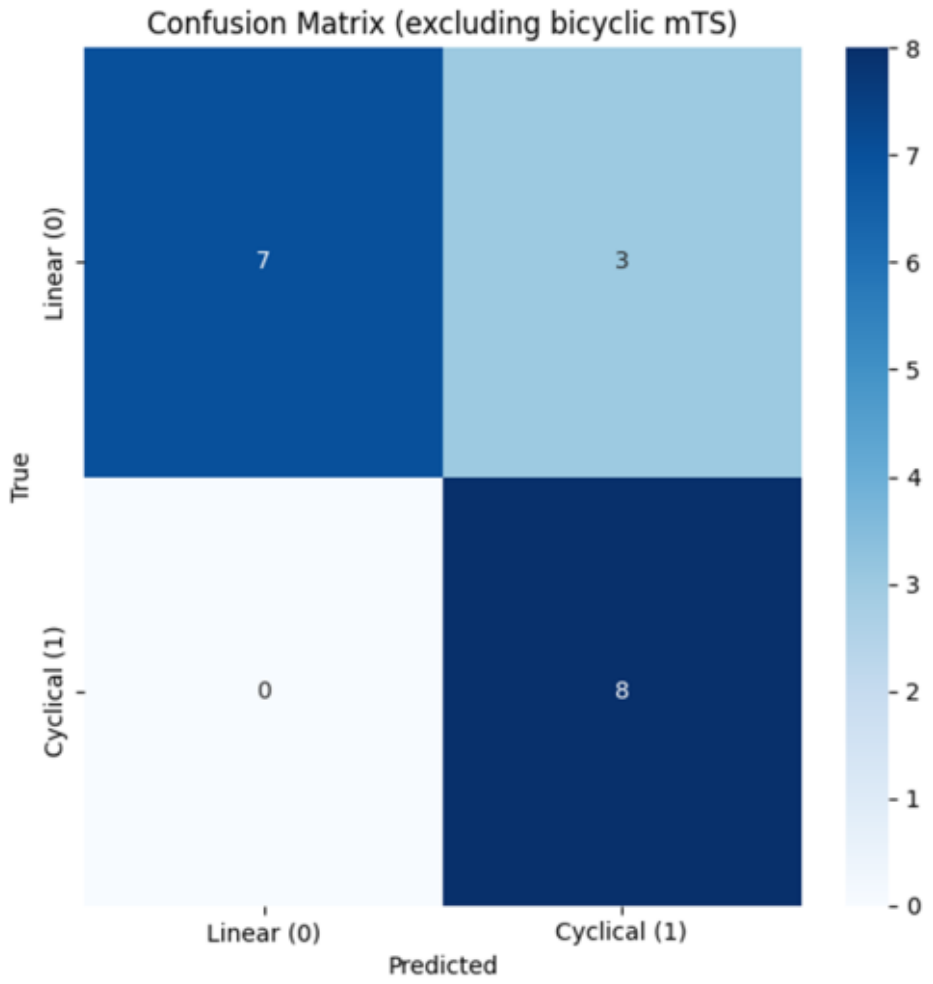

Figure S16: Evaluation of model transferability on an independent set of monoterpene synthases producing non-linalool/limonene products, selected to represent diverse species and product classes. A total of 27 enzymes were curated (10 linear, 8 monocyclic, 9 bicyclic), ensuring minimal species overlap within each group to reduce bias. (top) Overall classification performance, where enzymes producing linear products (e.g., β-myrcene, β-ocimene, geraniol) are treated as linear and those producing cyclic products (e.g., α/β-pinene, borneol, b-phellandrene) as cyclic, shows an accuracy of 70.4%, representing a reduction compared to the model performance reported in the main dataset, with similar performance across classes (linear: 70.0%, cyclic: 70.6%). Performance on bicyclic mTSs alone shows an accuracy (44.4%), with 5 enzymes incorrectly predicted as linear: three α-pinene producers (C3RSF5, Q84KL3, dinctg061np12121087) and two borneol producers (dalctg267np498, dgrChr5258), indicating that enzymes catalysing more complex, multi-step cyclisation reactions are not well captured by the current binary framework. (bottom) Excluding bicyclic enzymes, performance improves substantially (accuracy 83.3%), with perfect classification of monocyclic enzymes (cyclic class: 100%), consistent with the model capturing features associated with initial 1,6-cyclisation. Within the linear group, performance differences were observed between hydroxylated and non-hydroxylated products; 3 enzymes in total were misclassified as cyclic, including 1 geraniol producer (Q308N0) and 2 ocimene producers (B6SCF4, Q93X23), suggesting a potential bias arising from training solely on hydroxylated linear enzymes. Overall, these results indicate that the model captures determinants associated with early carbocation formation and 1,6-ring closure, while highlighting limitations in describing more complex bicyclic reaction pathways.

Table S20: Sesquiterpene synthases selected from a previously curated dataset[103,104] to assess the generalisability of the active-site classification workflow. Enzymes producing linear or 1,6-cyclised sesquiterpenes were included when complete sequences, suitable AlphaFold2 models, and appropriate Fpocket-identified active sites were available. The table lists the UniProt accession, species, and product classification for the 66 included enzymes, 39 linear and 27 monocyclic undergoing 1,6-cyclisation.

UNIPROT ID	SPECIES	CLASSIFICATION
**A0A076GAU9**	*Populus trichocarpa*	1,6-cyclised
**A0A0A0RDZ8**	*Santalum album*	1,6-cyclised
**A0A0E0LRP1**	*Oryza punctata*	1,6-cyclised
**A0A1J0CQ86**	*Pseudotaxiphyllum elegans*	1,6-cyclised
**A0A1J0CQ92**	*Anomodon rostratus*	1,6-cyclised
**A0A1L7NYF8**	*Artemisia maritima*	1,6-cyclised
**A0A1L7NYG3**	*Artemisia kurramensis*	1,6-cyclised

**A0A650A7H3**	*Oryza glaberrima*	1,6-cyclised
**A0A650A7I8**	*Oryza sativa Japonica Group*	1,6-cyclised
**A0A650A7K4**	*Oryza rufipogon*	1,6-cyclised
**A0A650A7M1**	*Oryza rufipogon*	1,6-cyclised
**A0A650A7N6**	*Oryza sativa f. spontanea*	1,6-cyclised
**A0A650A872**	*Oryza sativa f. spontanea*	1,6-cyclised
**C5H3I7**	*Oryza sativa*	1,6-cyclised
**C5YHH7**	*Sorghum bicolor*	1,6-cyclised
**C5YHI2**	*Sorghum bicolor*	1,6-cyclised
**C5YN30**	*Sorghum bicolor*	1,6-cyclised
**E3W205**	*Santalum austrocaledonicum*	1,6-cyclised
**E3W206**	*Santalum spicatum*	1,6-cyclised
**J3MQP7**	*Oryza brachyantha*	1,6-cyclised
**J7LH11**	*Phyla dulcis*	1,6-cyclised
**M4HZ33**	*Artemisia annua*	1,6-cyclised
**O81086**	*Abies grandis*	1,6-cyclised
**Q5SBP4**	*Ocimum basilicum*	1,6-cyclised
**Q675L6**	*Picea abies*	1,6-cyclised
**Q6JD73**	*Zea mays*	1,6-cyclised
**Q9T0K1**	*Arabidopsis thaliana*	1,6-cyclised
**A0A075W2D6**	*Matricaria chamomilla*	Linear
**A0A076GBT1**	*Populus trichocarpa*	Linear
**A0A0C5KR55**	*Phyllostachys edulis*	Linear
**A0A109QXM3**	*Osmanthus fragrans*	Linear
**A0A1J0CQ93**	*Scapania nemorea*	Linear
**A0A1J0CQ94**	*Myriopteris rufa*	Linear
**A0A1S5TFB0**	*Tripterygium wilfordii*	Linear
**A0A1U9M7X9**	*Murraya koenigii*	Linear
**B1NA83**	*Antirrhinum majus*	Linear
**B2KSJ6**	*Cucumis melo*	Linear
**B9RXW0**	*Ricinus communis*	Linear
**C7SHN9**	*Actinidia deliciosa*	Linear
**D7U6G8**	*Vitis vinifera*	Linear
**D8RNZ9**	*Selaginella moellendorffii*	Linear
**E5GAF9**	*Vitis vinifera*	Linear
**E5GAH2**	*Vitis vinifera*	Linear
**E5GAH3**	*Vitis vinifera*	Linear
**E5GAH4**	*Vitis vinifera*	Linear

**E5GAH5**	*Vitis vinifera*	Linear
**E5GAH6**	*Vitis vinifera*	Linear
**E5GAH7**	*Vitis vinifera*	Linear
**F2XF99**	*Picea engelmannii*	Linear
**F8TWD0**	*Populus trichocarpa*	Linear
**F8TWD1**	*Populus trichocarpa*	Linear
**F8TWD2**	*Populus trichocarpa*	Linear
**G5CV39**	*Solanum lycopersicum*	Linear
**G5CV41**	*Solanum lycopersicum*	Linear
**L0I8P4**	*Malus domestica*	Linear
**O48935**	*Mentha piperita*	Linear
**P0CV94**	*Fragaria ananassa*	Linear
**P0CV95**	*Fragaria ananassa*	Linear
**Q29VN2**	*Zea mays*	Linear
**Q4QSN5**	*Pseudotsuga menziesii*	Linear
**Q66PX9**	*Cucumis sativus*	Linear
**Q675K8**	*Picea abies*	Linear
**Q84KL5**	*Pinus taeda*	Linear
**Q84LB2**	*Malus domestica*	Linear
**Q94JS8**	*Citrus junos*	Linear
**Q9FXY7**	*Artemisia annua*	Linear

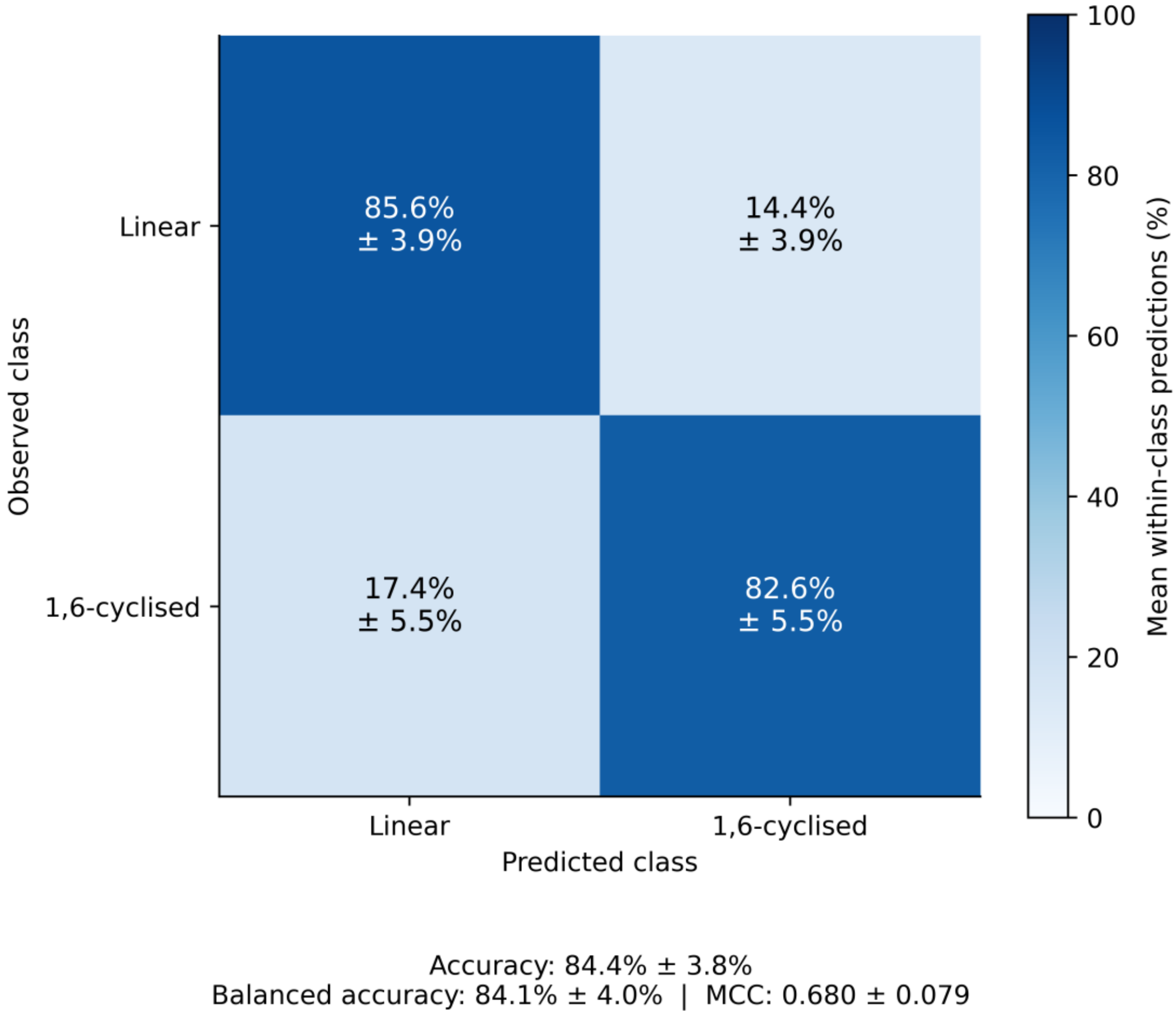

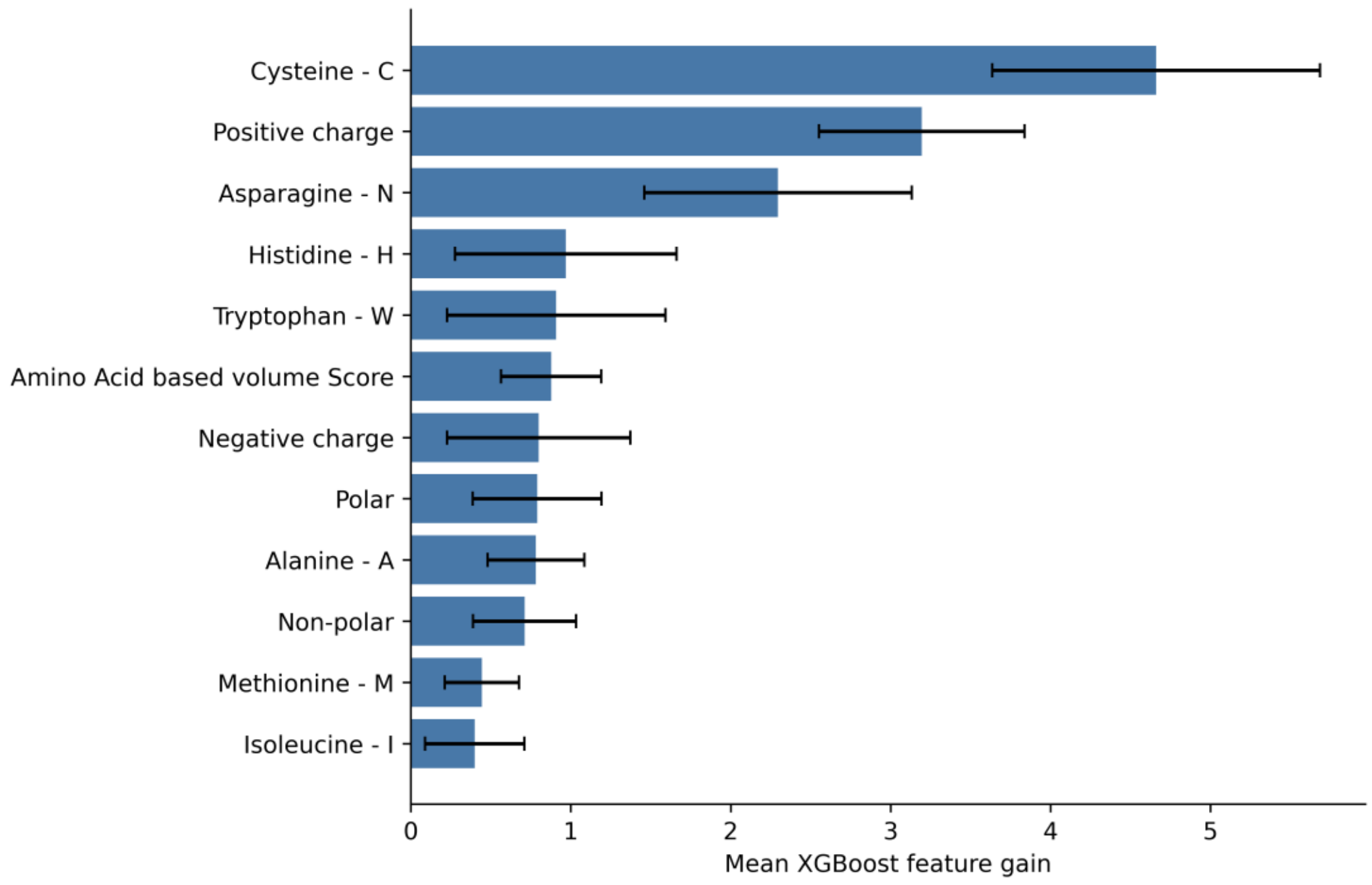

(continued on next page)

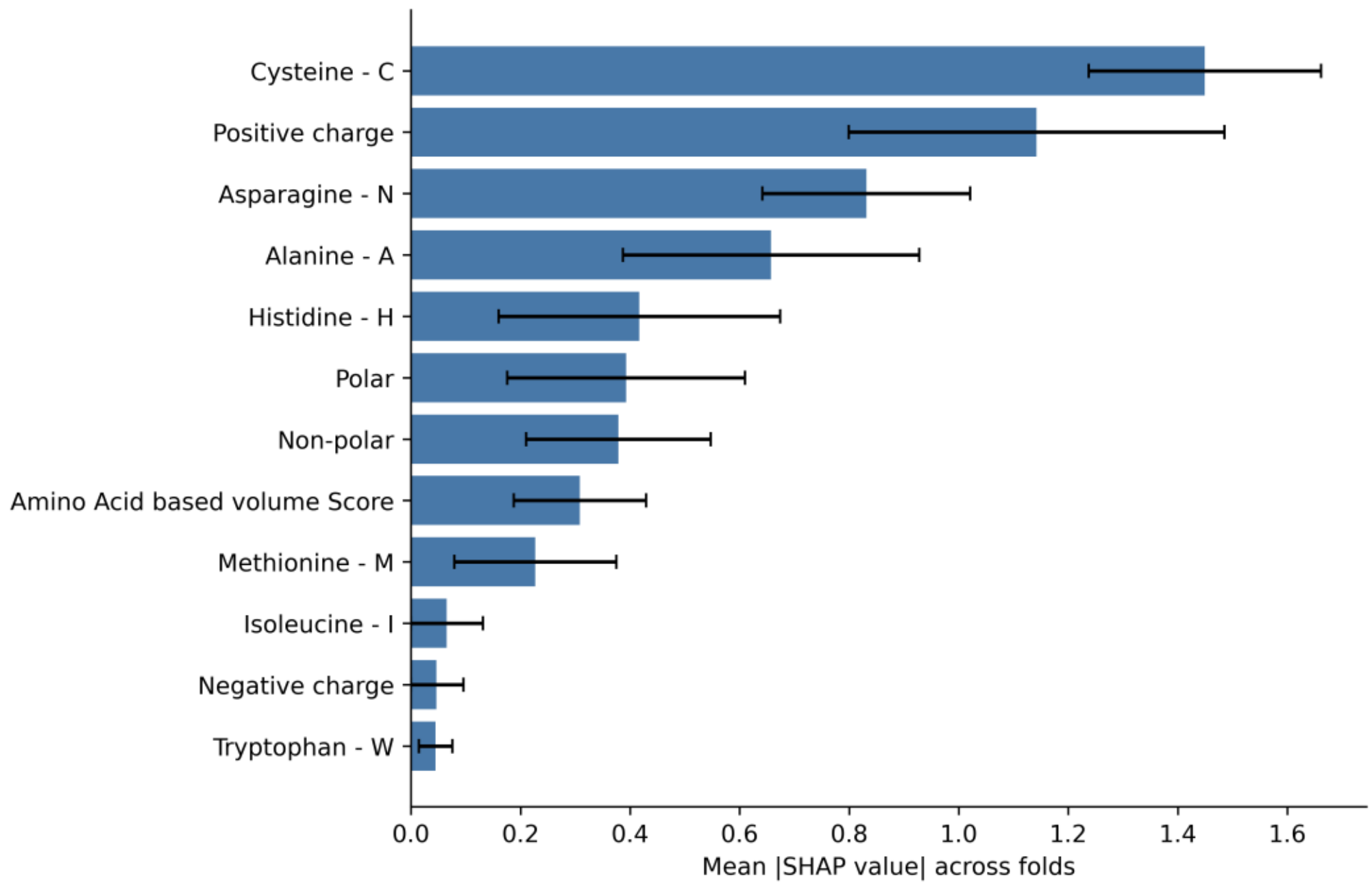

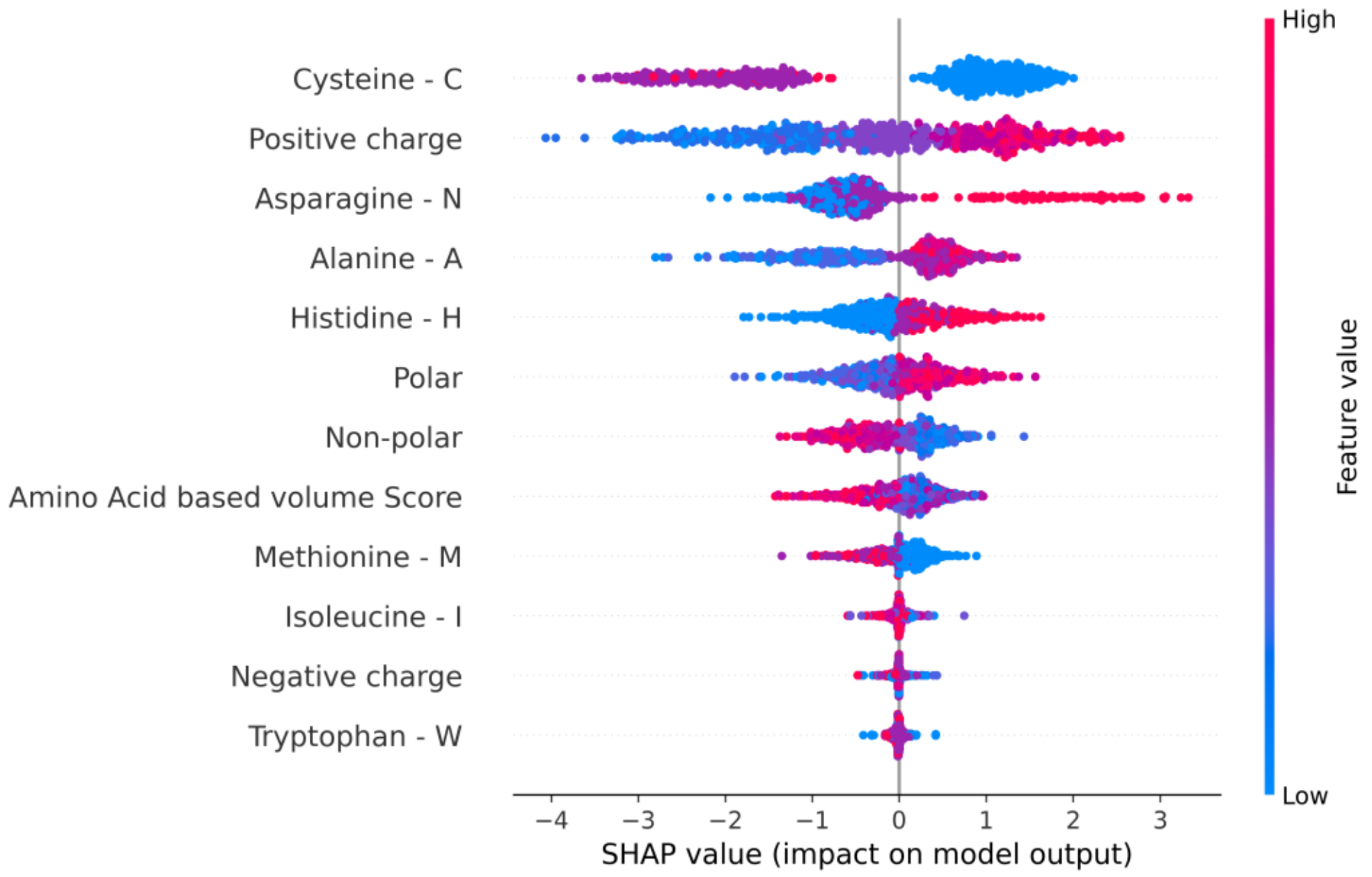

Figure S17: Preliminary classification of linear and 1,6-cyclised sesquiterpene synthases using the features and hyperparameters selected for monoterpene synthases, with class weights adjusted for the new dataset. No further feature selection or model optimisation was performed. Repeated stratified five-fold cross-validation across 66 enzymes gave a mean accuracy of 84.4% (SD 3.8%), balanced accuracy of 84.1% (SD 4.0%), and MCC of 0.680 (SD 0.079). For the linear class, mean precision was 87.7% (SD 3.7%), recall was 85.6% (SD 3.9%), specificity was 82.6% (SD 5.5%), and F1 score was 86.6% (SD 3.3%). For the 1,6-cyclised class, mean precision was 80.0% (SD 4.7%), recall was 82.6% (SD 5.5%), specificity was 85.6% (SD 3.9%), and F1 score was 81.2% (SD 4.6%). Shown are the repeated cross-validation confusion matrix (top), mean gain-based feature importance (upper middle), mean absolute SHAP values (lower middle), and SHAP beeswarm values (bottom).

Additional References:

(1) Aharoni, A.; Giri, A. P.; Verstappen, F. W. A.; Bertea, C. M.; Sevenier, R.; Sun, Z.; Jongsma, M. A.; Schwab, W.; Bouwmeester, H. J. Gain and Loss of Fruit Flavor Compounds Produced by Wild and Cultivated Strawberry Species. *Plant Cell* **2004**, *16* (11), 3110–3131. https://doi.org/10.1105/tpc.104.023895.

(2) Bohlmann, J.; Phillips, M.; Ramachandiran, V.; Katoh, S.; Croteau, R. cDNA Cloning, Characterization, and Functional Expression of Four New Monoterpene Synthase Members of the Tpsd Gene Family from Grand Fir (Abies Grandis). *Arch. Biochem. Biophys.* **1999**, *368* (2), 232–243. https://doi.org/10.1006/abbi.1999.1332.

(3) Byun-McKay, A.; Godard, K.-A.; Toudefallah, M.; Martin, D. M.; Alfaro, R.; King, J.; Bohlmann, J.; Plant, A. L. Wound-Induced Terpene Synthase Gene Expression in Sitka Spruce That Exhibit Resistance or Susceptibility to Attack by the White Pine Weevil. *Plant Physiol.* **2006**, *140* (3), 1009–1021. https://doi.org/10.1104/pp.105.071803.

(4) Chen, F.; Tholl, D.; D'Auria, J. C.; Farooq, A.; Pichersky, E.; Gershenzon, J. Biosynthesis and Emission of Terpenoid Volatiles from Arabidopsis Flowers. *Plant Cell* **2003**, *15* (2), 481–494. https://doi.org/10.1105/tpc.007989.

(5) Chen, Z.; Vining, K. J.; Qi, X.; Yu, X.; Zheng, Y.; Liu, Z.; Fang, H.; Li, L.; Bai, Y.; Liang, C.; Li, W.; Lange, B. M. Genome-Wide Analysis of Terpene Synthase Gene Family in Mentha Longifolia and Catalytic Activity Analysis of a Single Terpene Synthase. *Genes* **2021**, *12* (4), 518. https://doi.org/10.3390/genes12040518.

(6) Colby, S. M.; Alonso, W. R.; Katahira, E. J.; McGarvey, D. J.; Croteau, R. 4S-Limonene Synthase from the Oil Glands of Spearmint (Mentha Spicata). cDNA Isolation, Characterization, and Bacterial Expression of the Catalytically Active Monoterpene Cyclase. *J. Biol. Chem.* **1993**, *268* (31), 23016–23024. https://doi.org/10.1016/S0021-9258(19)49419-2.

(7) Crowell, A. L.; Williams, D. C.; Davis, E. M.; Wildung, M. R.; Croteau, R. Molecular Cloning and Characterization of a New Linalool Synthase. *Arch. Biochem. Biophys.* **2002**, *405* (1), 112–121. https://doi.org/10.1016/S0003-9861(02)00348-X.

(8) Danner, H.; Boeckler, G. A.; Irmisch, S.; Yuan, J. S.; Chen, F.; Gershenzon, J.; Unsicker, S. B.; Köllner, T. G. Four Terpene Synthases Produce Major Compounds of the Gypsy Moth Feeding-Induced Volatile Blend of *Populus Trichocarpa*. *Phytochemistry* **2011**, *72* (9), 897–908. https://doi.org/10.1016/j.phytochem.2011.03.014.

(9) Del Terra, L.; Lonzarich, V.; Asquini, E.; Navarini, L.; Graziosi, G.; Suggi Liverani, F.; Pallavicini, A. Functional Characterization of Three Coffea Arabica L. Monoterpene Synthases: Insights into the Enzymatic Machinery of Coffee Aroma. *Phytochemistry* **2013**, *89*, 6–14. https://doi.org/10.1016/j.phytochem.2013.01.005.

(10) Falara, V.; Akhtar, T. A.; Nguyen, T. T. H.; Spyropoulou, E. A.; Bleeker, P. M.; Schauvinhold, I.; Matsuba, Y.; Bonini, M. E.; Schilmiller, A. L.; Last, R. L.; Schuurink, R. C.; Pichersky, E. The Tomato Terpene Synthase Gene Family. *Plant Physiol.* **2011**, *157* (2), 770–789. https://doi.org/10.1104/pp.111.179648.

(11) Galata, M.; Sarker, L. S.; Mahmoud, S. S. Transcriptome Profiling, and Cloning and Characterization of the Main Monoterpene Synthases of *Coriandrum Sativum* L. *Phytochemistry* **2014**, *102*, 64–73. https://doi.org/10.1016/j.phytochem.2014.02.016.

(12) Günnewich, N.; Page, J. E.; Köllner, T. G.; Degenhardt, J.; Kutchan, T. M. Functional Expression and Characterization of Trichome-Specific (-)-Limonene Synthase and (+)-α-Pinene Synthase from Cannabis Sativa. *Nat. Prod. Commun.* **2007**, *2* (3), 1934578X0700200301. https://doi.org/10.1177/1934578X0700200301.

(13) Hurd, M. C.; Kwon, M.; Ro, D.-K. Functional Identification of a Lippia Dulcis Bornyl Diphosphate Synthase That Contains a Duplicated, Inhibitory Arginine-Rich Motif. *Biochem. Biophys. Res. Commun.* **2017**, *490* (3), 963–968. https://doi.org/10.1016/j.bbrc.2017.06.147.

(14) Iijima, Y.; Davidovich-Rikanati, R.; Fridman, E.; Gang, D. R.; Bar, E.; Lewinsohn, E.; Pichersky, E. The Biochemical and Molecular Basis for the Divergent Patterns in the

Biosynthesis of Terpenes and Phenylpropenes in the Peltate Glands of Three Cultivars of Basil. *Plant Physiol.* **2004**, *136* (3), 3724–3736. https://doi.org/10.1104/pp.104.051318.
(15) Jia, J.-W.; Crock, J.; Lu, S.; Croteau, R.; Chen, X.-Y. (3*R*)-Linalool Synthase from *Artemisia Annua* L.: cDNA Isolation, Characterization, and Wound Induction. *Arch. Biochem. Biophys.* **1999**, *372* (1), 143–149. https://doi.org/10.1006/abbi.1999.1466.
(16) Keeling, C. I.; Weisshaar, S.; Ralph, S. G.; Jancsik, S.; Hamberger, B.; Dullat, H. K.; Bohlmann, J. Transcriptome Mining, Functional Characterization, and Phylogeny of a Large Terpene Synthase Gene Family in Spruce (Piceaspp.). *BMC Plant Biol.* **2011**, *11* (1), 43. https://doi.org/10.1186/1471-2229-11-43.
(17) Landmann, C.; Fink, B.; Festner, M.; Dregus, M.; Engel, K.-H.; Schwab, W. Cloning and Functional Characterization of Three Terpene Synthases from Lavender (*Lavandula Angustifolia*). *Arch. Biochem. Biophys.* **2007**, *465* (2), 417–429. https://doi.org/10.1016/j.abb.2007.06.011.
(18) Lin, Y.-L.; Lee, Y.-R.; Huang, W.-K.; Chang, S.-T.; Chu, F.-H. Characterization of S-(+)-Linalool Synthase from Several Provenances of Cinnamomum Osmophloeum. *Tree Genet. Genomes* **2014**, *10* (1), 75–86. https://doi.org/10.1007/s11295-013-0665-1.
(19) Liu, G.; Yang, M.; Yang, X.; Ma, X.; Fu, J. Five TPSs Are Responsible for Volatile Terpenoid Biosynthesis in Albizia Julibrissin. *J. Plant Physiol.* **2021**, *258–259*, 153358. https://doi.org/10.1016/J.JPLPH.2020.153358.
(20) Lücker, J.; El Tamer, M. K.; Schwab, W.; Verstappen, F. W. A.; van der Plas, L. H. W.; Bouwmeester, H. J.; Verhoeven, H. A. Monoterpene Biosynthesis in Lemon (Citrus Limon). *Eur. J. Biochem.* **2002**, *269* (13), 3160–3171. https://doi.org/10.1046/j.1432-1033.2002.02985.x.
(21) Martin, D. M.; Fäldt, J.; Bohlmann, J. Functional Characterization of Nine Norway Spruce TPS Genes and Evolution of Gymnosperm Terpene Synthases of the TPS-d Subfamily. *Plant Physiol.* **2004**, *135* (4), 1908–1927. https://doi.org/10.1104/pp.104.042028.
(22) Maruyama, T.; Ito, M.; Kiuchi, F.; Honda, G. Molecular Cloning, Functional Expression and Characterization of d-Limonene Synthase from Schizonepeta Tenuifolia. *Biol. Pharm. Bull.* **2001**, *24* (4), 373–377. https://doi.org/10.1248/bpb.24.373.
(23) Masumoto, N.; Korin, M.; Ito, M. Geraniol and Linalool Synthases from Wild Species of Perilla. *Phytochemistry* **2010**, *71* (10), 1068–1075. https://doi.org/10.1016/j.phytochem.2010.04.006.
(24) Morehouse, B. R.; Kumar, R. P.; Matos, J. O.; Olsen, S. N.; Entova, S.; Oprian, D. D. Functional and Structural Characterization of a (+)-Limonene Synthase from Citrus Sinensis. *Biochemistry* **2017**, *56* (12), 1706–1715. https://doi.org/10.1021/acs.biochem.7b00143.
(25) Nagegowda, D. A.; Gutensohn, M.; Wilkerson, C. G.; Dudareva, N. Two Nearly Identical Terpene Synthases Catalyze the Formation of Nerolidol and Linalool in Snapdragon Flowers. *Plant J.* **2008**, *55* (2), 224–239. https://doi.org/10.1111/j.1365-313X.2008.03496.x.
(26) Nawade, B.; Yahyaa, M.; Reuveny, H.; Shaltiel-Harpaz, L.; Eisenbach, O.; Faigenboim, A.; Bar-Yaakov, I.; Holland, D.; Ibdah, M. Profiling of Volatile Terpenes from Almond (*Prunus Dulcis*) Young Fruits and Characterization of Seven Terpene Synthase Genes. *Plant Sci.* **2019**, *287*, 110187. https://doi.org/10.1016/j.plantsci.2019.110187.
(27) Rajaonarivony, J. I. M.; Gershenzon, J.; Croteau, R. Characterization and Mechanism of (4*S*)-Limonene Synthase, A Monoterpene Cyclase from the Glandular Trichomes of Peppermint (*Mentha x Piperita*). *Arch. Biochem. Biophys.* **1992**, *296* (1), 49–57. https://doi.org/10.1016/0003-9861(92)90543-6.
(28) Shimada, T.; Endo, T.; Fujii, H.; Omura, M. Isolation and Characterization of a New *d*-Limonene Synthase Gene with a Different Expression Pattern in *Citrus Unshiu* Marc. *Sci. Hortic.* **2005**, *105* (4), 507–512. https://doi.org/10.1016/j.scienta.2005.02.009.
(29) Sugiura, M.; Ito, S.; Saito, Y.; Niwa, Y.; Koltunw, A. M.; Sugimoto, O.; Sakai, H. Molecular Cloning and Characterization of a Linalool Synthase from Lemon Myrtle. *Biosci. Biotechnol. Biochem.* **2011**, *75* (7), 1245–1248. https://doi.org/10.1271/bbb.100922.

(30) Yuan, J. S.; Köllner, T. G.; Wiggins, G.; Grant, J.; Degenhardt, J.; Chen, F. Molecular and Genomic Basis of Volatile-Mediated Indirect Defense against Insects in Rice. *Plant J. Cell Mol. Biol.* **2008**, *55* (3), 491–503. https://doi.org/10.1111/j.1365-313X.2008.03524.x.
(31) Yuba, A.; Yazaki, K.; Tabata, M.; Honda, G.; Croteau, R. cDNA Cloning, Characterization, and Functional Expression of 4*S*-(−)-Limonene Synthase from*Perilla Frutescens*. *Arch. Biochem. Biophys.* **1996**, *332* (2), 280–287. https://doi.org/10.1006/abbi.1996.0343.
(32) Yue, Y.; Yu, R.; Fan, Y. Characterization of Two Monoterpene Synthases Involved in Floral Scent Formation in Hedychium Coronarium. *Planta* **2014**, *240* (4), 745–762. https://doi.org/10.1007/s00425-014-2127-x.
(33) Karuppiah, V.; Ranaghan, K. E.; Leferink, N. G. H.; Johannissen, L. O.; Shanmugam, M.; Cheallaigh, A. N.; Bennett, N. J.; Kearsey, L. J.; Takano, E.; Gardiner, J. M.; Kamp, M. W. V. D.; Hay, S.; Mulholland, A. J.; Leys, D.; Scrutton, N. S. Structural Basis of Catalysis in the Bacterial Monoterpene Synthases Linalool Synthase and 1,8-Cineole Synthase. *ACS Catal.* **2017**, *7* (9), 6268–6282. https://doi.org/10.1021/ACSCATAL.7B01924.
(34) Dudareva, N.; Cseke, L.; Blanc, V. M.; Pichersky, E. Evolution of Floral Scent in Clarkia: Novel Patterns of S-Linalool Synthase Gene Expression in the C. Breweri Flower. *Plant Cell* **1996**, *8* (7), 1137–1148. https://doi.org/10.1105/tpc.8.7.1137.
(35) Huang, K.-F.; Wen, C.-H.; Lee, Y.-R.; Chu, F.-H. Cloning and Characterization of Terpene Synthase Genes from Taiwan Cherry. *Tree Genet. Genomes* **2019**, *15* (4), 51. https://doi.org/10.1007/s11295-019-1355-4.
(36) Huang, K.-F.; Lee, Y.-R.; Tseng, Y.-H.; Wang, S.-Y.; Chu, F.-H. Cloning and Functional Characterization of a Monoterpene Synthase Gene from Eleutherococcus Trifoliatus. *Holzforschung* **2015**, *69* (2), 163–171. https://doi.org/10.1515/hf-2014-0078.
(37) Jongedijk, E.; Cankar, K.; Ranzijn, J.; van der Krol, S.; Bouwmeester, H.; Beekwilder, J. Capturing of the Monoterpene Olefin Limonene Produced in Saccharomyces Cerevisiae. *Yeast Chichester Engl.* **2015**, *32* (1), 159–171. https://doi.org/10.1002/yea.3038.
(38) Hattan, J.; Shindo, K.; Ito, T.; Shibuya, Y.; Watanabe, A.; Tagaki, C.; Ohno, F.; Sasaki, T.; Ishii, J.; Kondo, A.; Misawa, N. Identification of a Novel Hedycaryol Synthase Gene Isolated from Camellia Brevistyla Flowers and Floral Scent of Camellia Cultivars. *Planta* **2016**, *243* (4), 959–972. https://doi.org/10.1007/s00425-015-2454-6.
(39) Yang, Z.; Li, Y.; Gao, F.; Jin, W.; Li, S.; Kimani, S.; Yang, S.; Bao, T.; Gao, X.; Wang, L. MYB21 Interacts with MYC2 to Control the Expression of Terpene Synthase Genes in Flowers of Freesia Hybrida and Arabidopsis Thaliana. *J. Exp. Bot.* **2020**, *71* (14), 4140–4158. https://doi.org/10.1093/jxb/eraa184.
(40) Magnard, J.-L.; Bony, A. R.; Bettini, F.; Campanaro, A.; Blerot, B.; Baudino, S.; Jullien, F. Linalool and Linalool Nerolidol Synthases in Roses, Several Genes for Little Scent. *Plant Physiol. Biochem. PPB* **2018**, *127*, 74–87. https://doi.org/10.1016/j.plaphy.2018.03.009.
(41) Adal, A. M.; Sarker, L. S.; Malli, R. P. N.; Liang, P.; Mahmoud, S. S. RNA-Seq in the Discovery of a Sparsely Expressed Scent-Determining Monoterpene Synthase in Lavender (Lavandula). *Planta* **2019**, *249* (1), 271–290. https://doi.org/10.1007/s00425-018-2935-5.
(42) Hattan, J.; Shindo, K.; Sasaki, T.; Misawa, N. Isolation and Functional Characterization of New Terpene Synthase Genes from Traditional Edible Plants. *J. Oleo Sci.* **2018**, *67* (10), 1235–1246. https://doi.org/10.5650/jos.ess18163.
(43) Liu, M.; Liu, X.; Hu, J.; Xue, Y.; Zhao, X. Genetic Diversity of Limonene Synthase Genes in Rongan Kumquat (Fortunella Crassifolia). *Funct. Plant Biol.* **2020**, *47* (5), 425–439. https://doi.org/10.1071/FP19051.
(44) Zager, J. J.; Lange, I.; Srividya, N.; Smith, A.; Lange, B. M. Gene Networks Underlying Cannabinoid and Terpenoid Accumulation in Cannabis. *Plant Physiol.* **2019**, *180* (4), 1877–1897. https://doi.org/10.1104/pp.18.01506.
(45) Zhou, Y.; Deng, R.; Xu, X.; Yang, Z. Enzyme Catalytic Efficiencies and Relative Gene Expression Levels of (R)-Linalool Synthase and (S)-Linalool Synthase Determine the

Proportion of Linalool Enantiomers in Camellia Sinensis Var. Sinensis. *J. Agric. Food Chem.* **2020**, *68* (37), 10109–10117. https://doi.org/10.1021/acs.jafc.0c04381.
(46) Nawade, B.; Shaltiel-Harpaz, L.; Yahyaa, M.; Kabaha, A.; Kedoshim, R.; Bosamia, T. C.; Ibdah, M. Characterization of Terpene Synthase Genes Potentially Involved in Black Fig Fly *(Silba Adipata*) Interactions with *Ficus Carica*. *Plant Sci.* **2020**, *298*, 110549. https://doi.org/10.1016/j.plantsci.2020.110549.
(47) Yang, P.; Zhao, H.-Y.; Wei, J.-S.; Zhao, Y.-Y.; Lin, X.-J.; Su, J.; Li, F.-P.; Li, M.; Ma, D.-M.; Tan, X.-K.; Liang, H.-L.; Sun, Y.-W.; Zhan, R.-T.; He, G.-Z.; Zhou, X.-F.; Yang, J.-F. Chromosome-Level Genome Assembly and Functional Characterization of Terpene Synthases Provide Insights into the Volatile Terpenoid Biosynthesis of Wurfbainia Villosa. *Plant J.* **2022**, *112* (3), 630–645. https://doi.org/10.1111/tpj.15968.
(48) Chen, R.; Hu, J.; Pan, Y.; Song, H.; Xian, X. Isolation and Expression Analysis of OfLIS Gene in Osmanthus Fragrans. **2020**, *24* (6).
(49) Chen, X.; Yauk, Y.-K.; Nieuwenhuizen, N. J.; Matich, A. J.; Wang, M. Y.; Perez, R. L.; Atkinson, R. G.; Beuning, L. L. Characterisation of an (S)-Linalool Synthase from Kiwifruit (Actinidia Arguta) That Catalyses the First Committed Step in the Production of Floral Lilac Compounds. *Funct. Plant Biol.* **2010**, *37* (3), 232–243. https://doi.org/10.1071/FP09179.
(50) Feng, L.; Chen, C.; Li, T.; Wang, M.; Tao, J.; Zhao, D.; Sheng, L. Flowery Odor Formation Revealed by Differential Expression of Monoterpene Biosynthetic Genes and Monoterpene Accumulation in Rose (*Rosa Rugosa* Thunb.). *Plant Physiol. Biochem.* **2014**, *75*, 80–88. https://doi.org/10.1016/j.plaphy.2013.12.006.
(51) Zhu, B.-Q.; Cai, J.; Wang, Z.-Q.; Xu, X.-Q.; Duan, C.-Q.; Pan, Q.-H. Identification of a Plastid-Localized Bifunctional Nerolidol/Linalool Synthase in Relation to Linalool Biosynthesis in Young Grape Berries. *Int. J. Mol. Sci.* **2014**, *15* (12), 21992–22010. https://doi.org/10.3390/ijms151221992.
(52) Ito, M.; Kiuchi, F.; Yang, L. L.; Honda, G. Perilla Citriodora from Taiwan and Its Phytochemical Characteristics. *Biol. Pharm. Bull.* **2000**, *23* (3), 359–362. https://doi.org/10.1248/bpb.23.359.
(53) Lu, X.; Bai, J.; Tian, Z.; Li, C.; Ahmed, N.; Liu, X.; Cheng, J.; Lu, L.; Cai, J.; Jiang, H.; Wang, W. Cyclization Mechanism of Monoterpenes Catalyzed by Monoterpene Synthases in Dipterocarpaceae. *Synth. Syst. Biotechnol.* **2024**, *9* (1), 11–18. https://doi.org/10.1016/j.synbio.2023.11.009.
(54) Letunic, I.; Bork, P. Interactive Tree of Life (iTOL) v6: Recent Updates to the Phylogenetic Tree Display and Annotation Tool. *Nucleic Acids Res.* **2024**, *52* (W1), W78–W82. https://doi.org/10.1093/nar/gkae268.
(55) Le Guilloux, V.; Schmidtke, P.; Tuffery, P. Fpocket: An Open Source Platform for Ligand Pocket Detection. *BMC Bioinformatics* **2009**, *10* (1), 168. https://doi.org/10.1186/1471-2105-10-168.
(56) Livingstone, C. D.; Barton, G. J. Protein Sequence Alignments: A Strategy for the Hierarchical Analysis of Residue Conservation. *Comput. Appl. Biosci. CABIOS* **1993**, *9* (6), 745–756. https://doi.org/10.1093/BIOINFORMATICS/9.6.745.
(57) Saldaño, T.; Escobedo, N.; Marchetti, J.; Zea, D. J.; Mac Donagh, J.; Velez Rueda, A. J.; Gonik, E.; García Melani, A.; Novomisky Nechcoff, J.; Salas, M. N.; Peters, T.; Demitroff, N.; Fernandez Alberti, S.; Palopoli, N.; Fornasari, M. S.; Parisi, G. Impact of Protein Conformational Diversity on AlphaFold Predictions. *Bioinformatics* **2022**, *38* (10), 2742–2748. https://doi.org/10.1093/bioinformatics/btac202.
(58) Chen, T.; Guestrin, C. XGBoost: A Scalable Tree Boosting System. In *Proceedings of the 22nd ACM SIGKDD International Conference on Knowledge Discovery and Data Mining*; KDD '16; Association for Computing Machinery: New York, NY, USA, 2016; pp 785–794. https://doi.org/10.1145/2939672.2939785.
(59) Chen, C.; Zhang, Q.; Yu, B.; Yu, Z.; Lawrence, P. J.; Ma, Q.; Zhang, Y. Improving Protein-Protein Interactions Prediction Accuracy Using XGBoost Feature Selection and Stacked

Ensemble Classifier. *Comput. Biol. Med.* **2020**, *123*, 103899. https://doi.org/10.1016/j.compbiomed.2020.103899.
(60) Zhong, J.; Sun, Y.; Peng, W.; Xie, M.; Yang, J.; Tang, X. XGBFEMF: An XGBoost-Based Framework for Essential Protein Prediction. *IEEE Trans. NanoBioscience* **2018**, *17* (3), 243–250. https://doi.org/10.1109/TNB.2018.2842219.
(61) Sena, J.; Johannissen, L. O.; Blaker, J. J.; Hay, S. A Machine Learning Model for the Prediction of Water Contact Angles on Solid Polymers. *J. Phys. Chem. B* **2025**, *129* (10), 2739–2745. https://doi.org/10.1021/acs.jpcb.4c06608.
(62) Liang, W.; Zheng, S.; Shu, Y.; Huang, J. Bayesian Optimization-Assisted Engineering of Formate Dehydrogenase Encapsulation in Multivariate Zeolitic Imidazolate Framework. *Chem. Mater.* **2025**, *37* (1), 429–440. https://doi.org/10.1021/acs.chemmater.4c02816.
(63) Yang, K.; Liu, L.; Wen, Y. The Impact of Bayesian Optimization on Feature Selection. *Sci. Rep.* **2024**, *14* (1), 3948. https://doi.org/10.1038/s41598-024-54515-w.
(64) Luntz, A.; Brailovsky, V. On Estimation of Characters Obtained in Statistical Procedure of Recognition. *Tech. Kibern.* **1969**.
(65) Lachenbruch, P. A.; Mickey, M. R. Estimation of Error Rates in Discriminant Analysis. *Technometrics* **1968**, *10* (1), 1–11. https://doi.org/10.2307/1266219.
(66) Raschka, S. Model Evaluation, Model Selection, and Algorithm Selection in Machine Learning. arXiv November 11, 2020. https://doi.org/10.48550/arXiv.1811.12808.
(67) Swan, A. L.; Mobasheri, A.; Allaway, D.; Liddell, S.; Bacardit, J. Application of Machine Learning to Proteomics Data: Classification and Biomarker Identification in Postgenomics Biology. *OMICS J. Integr. Biol.* **2013**, *17* (12), 595–610. https://doi.org/10.1089/omi.2013.0017.
(68) Goldman, S.; Das, R.; Yang, K. K.; Coley, C. W. Machine Learning Modeling of Family Wide Enzyme-Substrate Specificity Screens. *PLoS Comput. Biol.* **2022**, *18* (2), e1009853. https://doi.org/10.1371/journal.pcbi.1009853.
(69) De Ferrari, L.; Mitchell, J. B. From Sequence to Enzyme Mechanism Using Multi-Label Machine Learning. *BMC Bioinformatics* **2014**, *15* (1), 150. https://doi.org/10.1186/1471-2105-15-150.
(70) Rajaonarivony, J. I. M.; Gershenzon, J.; Miyazaki, J.; Croteau, R. Evidence for an Essential Histidine Residue in 4*S*-Limonene Synthase and Other Terpene Cyclases. *Arch. Biochem. Biophys.* **1992**, *299* (1), 77–82. https://doi.org/10.1016/0003-9861(92)90246-S.
(71) Srividya, N.; Davis, E. M.; Croteau, R. B.; Lange, B. M. Functional Analysis of (4S)-Limonene Synthase Mutants Reveals Determinants of Catalytic Outcome in a Model Monoterpene Synthase. *Proc. Natl. Acad. Sci. U. S. A.* **2015**, *112* (11), 3332–3337. https://doi.org/10.1073/PNAS.1501203112/-/DCSUPPLEMENTAL.
(72) Xu, J.; Ai, Y.; Wang, J.; Xu, J.; Zhang, Y.; Yang, D. Converting S-Limonene Synthase to Pinene or Phellandrene Synthases Reveals the Plasticity of the Active Site. *Phytochemistry* **2017**, *137*, 34–41. https://doi.org/10.1016/J.PHYTOCHEM.2017.02.017.
(73) Hyatt, D. C.; Croteau, R. Mutational Analysis of a Monoterpene Synthase Reaction: Altered Catalysis through Directed Mutagenesis of (−)-Pinene Synthase from *Abies Grandis*. *Arch. Biochem. Biophys.* **2005**, *439* (2), 222–233. https://doi.org/10.1016/j.abb.2005.05.017.
(74) Christianson, D. W. Structural and Chemical Biology of Terpenoid Cyclases. *Chem. Rev.* **2017**, *117* (17), 11570. https://doi.org/10.1021/ACS.CHEMREV.7B00287.
(75) Whitehead, J. N.; Leferink, N. G. H.; Johannissen, L. O.; Hay, S.; Scrutton, N. S. Decoding Catalysis by Terpene Synthases. *ACS Catal.* **2023**, *13* (19), 12774–12802. https://doi.org/10.1021/acscatal.3c03047.
(76) Xu, J.; Xu, J.; Ai, Y.; Farid, R. A.; Tong, L.; Yang, D. Mutational Analysis and Dynamic Simulation of S-Limonene Synthase Reveal the Importance of Y573: Insight into the Cyclization Mechanism in Monoterpene Synthases. *Arch. Biochem. Biophys.* **2018**, *638*, 27–34. https://doi.org/10.1016/j.abb.2017.12.007.

(77) Major, D. T. Electrostatic Control of Chemistry in Terpene Cyclases. *ACS Catal.* **2017**, *7* (8), 5461–5465. https://doi.org/10.1021/acscatal.7b01328.
(78) Leferink, N. G. H.; Ranaghan, K. E.; Battye, J.; Johannissen, L. O.; Hay, S.; Kamp, M. W. van der; Mulholland, A. J.; Scrutton, N. S. Taming the Reactivity of Monoterpene Synthases To Guide Regioselective Product Hydroxylation. *ChemBioChem* **2020**, *21* (7), 985–990. https://doi.org/10.1002/CBIC.201900672.
(79) Leferink, N. G. H.; Escorcia, A. M.; Ouwersloot, B. R.; Johanissen, L. O.; Hay, S.; Kamp, M. W. van der; Scrutton, N. S. Molecular Determinants of Carbocation Cyclisation in Bacterial Monoterpene Synthases. *ChemBioChem* **2022**, *23* (5), e202100688. https://doi.org/10.1002/CBIC.202100688.
(80) Leferink, N. G. H.; Ranaghan, K. E.; Karuppiah, V.; Currin, A.; Kamp, M. W. V. D.; Mulholland, A. J.; Scrutton, N. S. Experiment and Simulation Reveal How Mutations in Functional Plasticity Regions Guide Plant Monoterpene Synthase Product Outcome. *ACS Catal.* **2019**, *8* (5), 3780–3791. https://doi.org/10.1021/ACSCATAL.8B00692.
(81) Kampranis, S. C.; Ioannidis, D.; Purvis, A.; Mahrez, W.; Ninga, E.; Katerelos, N. A.; Anssour, S.; Dunwell, J. M.; Degenhardt, J.; Makris, A. M.; Goodenough, P. W.; Johnsona, C. B. Rational Conversion of Substrate and Product Specificity in a Salvia Monoterpene Synthase: Structural Insights into the Evolution of Terpene Synthase Function. *Plant Cell* **2007**, *19* (6), 1994–2005. https://doi.org/10.1105/TPC.106.047779.
(82) Liu, D.; Li, N.; Yu, F.; Du, Y.; Song, H.; Yao, W. Mechanistic Insights into the Bornyl Diphosphate Synthase from Lavandula Angustifolia. *Curr. Issues Mol. Biol.* **2025**, *47* (7), 517. https://doi.org/10.3390/cimb47070517.
(83) Schiff, W. H.; Oprian, D. D. Mutational Analysis of (+)-Limonene Synthase. *Biochemistry* **2023**, *62* (16), 2472–2479. https://doi.org/10.1021/acs.biochem.3c00217.
(84) Engst, M.; Brokeš, M.; Čalounová, T.; Tajovská, A.; Perković, M.; Samusevich, R.; Chatpatanasiri, R.; Pluskal, T. MARTS-DB: A Database of Mechanisms And Reactions of Terpene Synthases. *BMC Bioinformatics* **2025**, *27* (1), 10. https://doi.org/10.1186/s12859-025-06341-8.
(85) Lin, Z.; Akin, H.; Rao, R.; Hie, B.; Zhu, Z.; Lu, W.; Smetanin, N.; Verkuil, R.; Kabeli, O.; Shmueli, Y.; dos Santos Costa, A.; Fazel-Zarandi, M.; Sercu, T.; Candido, S.; Rives, A. Evolutionary-Scale Prediction of Atomic-Level Protein Structure with a Language Model. *Science* **2023**, *379* (6637), 1123–1130. https://doi.org/10.1126/science.ade2574.
(86) Camacho, C.; Coulouris, G.; Avagyan, V.; Ma, N.; Papadopoulos, J.; Bealer, K.; Madden, T. L. BLAST+: Architecture and Applications. *BMC Bioinformatics* **2009**, *10* (1), 421. https://doi.org/10.1186/1471-2105-10-421.
(87) Eddy, S. R. Accelerated Profile HMM Searches. *PLOS Comput. Biol.* **2011**, *7* (10), e1002195. https://doi.org/10.1371/journal.pcbi.1002195.
(88) van Kempen, M.; Kim, S. S.; Tumescheit, C.; Mirdita, M.; Lee, J.; Gilchrist, C. L. M.; Söding, J.; Steinegger, M. Fast and Accurate Protein Structure Search with Foldseek. *Nat. Biotechnol.* **2024**, *42* (2), 243–246. https://doi.org/10.1038/s41587-023-01773-0.
(89) Yang, T.; Li, J.; Wang, H.-X.; Zeng, Y. A Geraniol-Synthase Gene from *Cinnamomum Tenuipilum*. *Phytochemistry* **2005**, *66* (3), 285–293. https://doi.org/10.1016/j.phytochem.2004.12.004.
(90) Burkhardt, I.; de Rond, T.; Chen, P. Y.-T.; Moore, B. S. Ancient Plant-like Terpene Biosynthesis in Corals. *Nat. Chem. Biol.* **2022**, *18* (6), 664–669. https://doi.org/10.1038/s41589-022-01026-2.
(91) Phillips, M. A.; Wildung, M. R.; Williams, D. C.; Hyatt, D. C.; Croteau, R. cDNA Isolation, Functional Expression, and Characterization of (+)-α-Pinene Synthase and (−)-α-Pinene Synthase from Loblolly Pine (*Pinus Taeda*): Stereocontrol in Pinene Biosynthesis. *Arch. Biochem. Biophys.* **2003**, *411* (2), 267–276. https://doi.org/10.1016/S0003-9861(02)00746-4.

(92) Chu, F.-H.; Kuo, P.-M.; Chen, Y.-R.; Wang, S.-Y. Cloning and Characterization of α-Pinene Synthase from Chamaecyparis Formosensis Matsum. **2009**, *63* (1), 69–74. https://doi.org/10.1515/HF.2009.019.
(93) Lu, S.; Xu, R.; Jia, J.-W.; Pang, J.; Matsuda, S. P. T.; Chen, X.-Y. Cloning and Functional Characterization of a β-Pinene Synthase from Artemisia Annua That Shows a Circadian Pattern of Expression. *Plant Physiol.* **2002**, *130* (1), 477–486. https://doi.org/10.1104/pp.006544.
(94) Liu, S.; Han, B. Differential Expression Pattern of an Acidic 9/13-Lipoxygenase in Flower Opening and Senescence and in Leaf Response to Phloem Feeders in the Tea Plant. *BMC Plant Biol.* **2010**, *10* (1), 228. https://doi.org/10.1186/1471-2229-10-228.
(95) Rudolph, K.; Parthier, C.; Egerer-Sieber, C.; Geiger, D.; Muller, Y. A.; Kreis, W.; Müller-Uri, F. Expression, Crystallization and Structure Elucidation of γ-Terpinene Synthase from Thymus Vulgaris. *Acta Crystallogr. Sect. F Struct. Biol. Commun.* **2016**, *72* (Pt 1), 16–23. https://doi.org/10.1107/S2053230X15023043.
(96) Ito, M.; Honda, G. Geraniol Synthases from Perilla and Their Taxonomical Significance. *Phytochemistry* **2007**, *68* (4), 446–453. https://doi.org/10.1016/j.phytochem.2006.11.006.
(97) Fischbach, R. J.; Zimmer, W.; Schnitzler, J.-P. Isolation and Functional Analysis of a cDNA Encoding a Myrcene Synthase from Holm Oak (Quercus Ilex L.). *Eur. J. Biochem.* **2001**, *268* (21), 5633–5638. https://doi.org/10.1046/j.1432-1033.2001.02519.x.
(98) Chang, Y.-T.; Chu, F.-H. Molecular Cloning and Characterization of Monoterpene Synthases from Litsea Cubeba (Lour.) Persoon. *Tree Genet. Genomes* **2011**, *7* (4), 835–844. https://doi.org/10.1007/s11295-011-0377-3.
(99) Bohlmann, J.; Steele, C. L.; Croteau, R. Monoterpene Synthases from Grand Fir (Abies Grandis): cDNA ISOLATION, CHARACTERIZATION, AND FUNCTIONAL EXPRESSION OF MYRCENE SYNTHASE, (−)-(4S)-LIMONENE SYNTHASE, AND (−)-(1S,5S)-PINENE SYNTHASE *. *J. Biol. Chem.* **1997**, *272* (35), 21784–21792. https://doi.org/10.1074/jbc.272.35.21784.
(100) Wang, G.; Tian, L.; Aziz, N.; Broun, P.; Dai, X.; He, J.; King, A.; Zhao, P. X.; Dixon, R. A. Terpene Biosynthesis in Glandular Trichomes of Hop. *Plant Physiol.* **2008**, *148* (3), 1254–1266. https://doi.org/10.1104/pp.108.125187.
(101) Booth, J. K.; Page, J. E.; Bohlmann, J. Terpene Synthases from Cannabis Sativa. *PLOS ONE* **2017**, *12* (3), e0173911. https://doi.org/10.1371/journal.pone.0173911.
(102) Hall, D. E.; Yuen, M. M. S.; Jancsik, S.; Quesada, A. L.; Dullat, H. K.; Li, M.; Henderson, H.; Arango-Velez, A.; Liao, N. Y.; Docking, R. T.; Chan, S. K.; Cooke, J. E.; Breuil, C.; Jones, S. J.; Keeling, C. I.; Bohlmann, J. Transcriptome Resources and Functional Characterization of Monoterpene Synthases for Two Host Species of the Mountain Pine Beetle, Lodgepole Pine (Pinus Contorta) and Jack Pine (Pinus Banksiana). *BMC Plant Biol.* **2013**, *13* (1), 80. https://doi.org/10.1186/1471-2229-13-80.
(103) Durairaj, J.; Di Girolamo, A.; Bouwmeester, H. J.; de Ridder, D.; Beekwilder, J.; van Dijk, A. DJ. An Analysis of Characterized Plant Sesquiterpene Synthases. *Phytochemistry* **2019**, *158*, 157–165. https://doi.org/10.1016/j.phytochem.2018.10.020.
(104) Durairaj, J.; Melillo, E.; Bouwmeester, H. J.; Beekwilder, J.; Ridder, D. de; Dijk, A. D. J. van. Integrating Structure-Based Machine Learning and Co-Evolution to Investigate Specificity in Plant Sesquiterpene Synthases. *PLOS Comput. Biol.* **2021**, *17* (3), e1008197. https://doi.org/10.1371/journal.pcbi.1008197.
